# Design, synthesis and biological evaluation of 9-aryl-5*H*-pyrido[4,3-*b*]indole derivatives as potential tubulin polymerization inhibitors

**DOI:** 10.3389/fchem.2022.1004835

**Published:** 2022-09-15

**Authors:** Lingyu Shi, Shanbo Yang, Jing Chang, Yujing Zhang, Wenjing Liu, Jun Zeng, Jingsen Meng, Renshuai Zhang, Chao Wang, Dongming Xing

**Affiliations:** ^1^ Cancer Institute, The Affiliated Hospital of Qingdao University and School of Basic Medicine, Qingdao University, Qingdao, China; ^2^ Qingdao Cancer Institute, Qingdao, China; ^3^ The Affiliated Cardiovascular Hospital of Qingdao University, Qingdao University, Qingdao, China; ^4^ School of Life Sciences, Tsinghua University, Beijing, China

**Keywords:** tubulin, pyrido[4,3-b]indole, antitumor activity, molecular docking, tubulin polymerization inhibitors

## Abstract

A series of new 9-aryl-5*H*-pyrido[4,3-*b*]indole derivatives as tubulin polymerization inhibitors were designed, synthesized, and evaluated for antitumor activity. All newly prepared compounds were tested for their anti-proliferative activity *in vitro* against three different cancer cells (SGC-7901, HeLa, and MCF-7). Among the designed compounds, compound **7k** displayed the strongest anti-proliferative activity against HeLa cells with IC_50_ values of 8.7 ± 1.3 μM. In addition, **7k** could inhibit the polymerization of tubulin and disrupt the microtubule network of cells. Further mechanism studies revealed that **7k** arrested cell cycle at the G2/M phase and induced apoptosis in a dose-dependent manner. Molecular docking analysis confirmed that **7k** may bind to colchicine binding sites on microtubules. Our study aims to provide a new strategy for the development of antitumor drugs targeting tubulin.

## 1 Introduction

**FIGURE 1:**
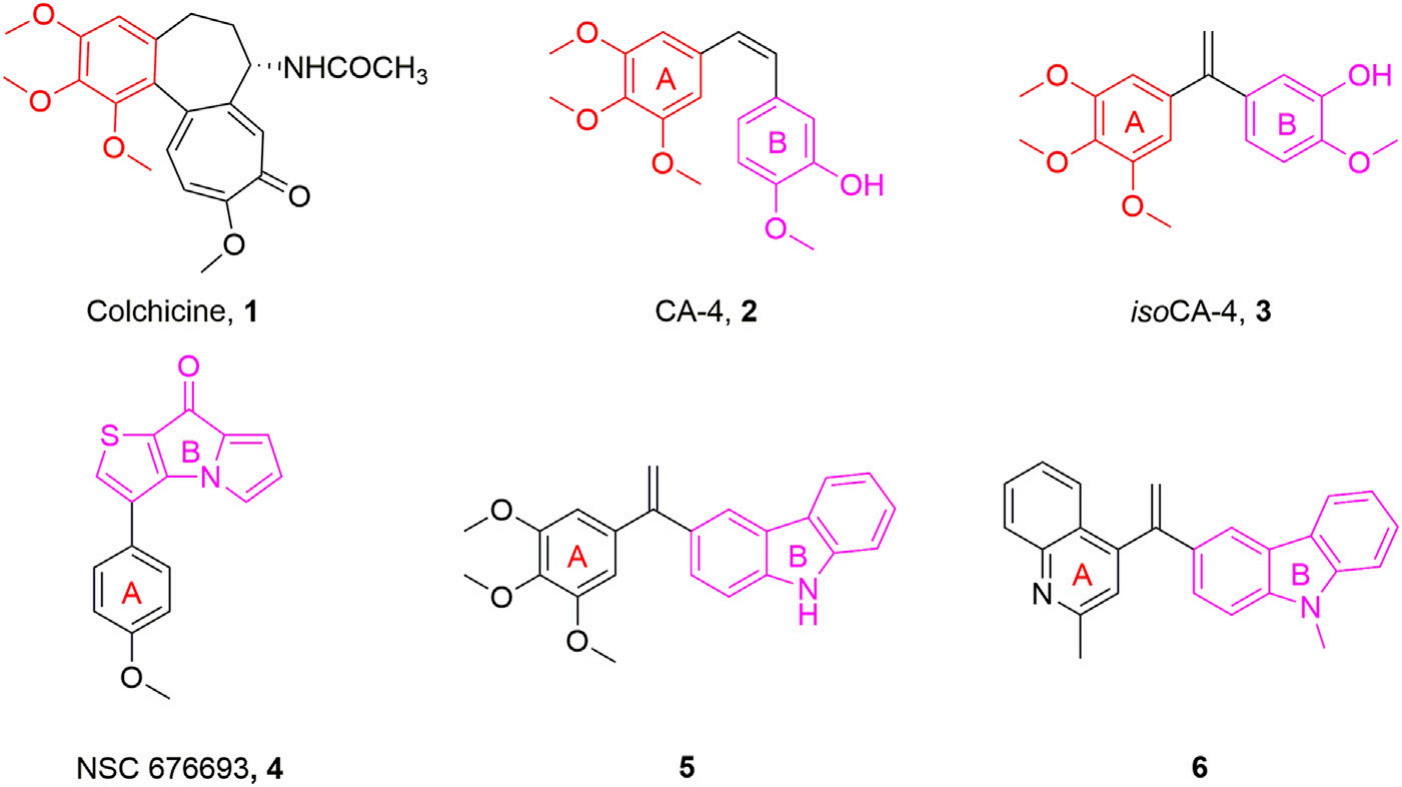
Chemical structures of some typical tubulin polymerization inhibitors.

Microtubules, crucial elements of the cytoskeleton, are highly dynamic frameworks formed by *α*- and *β*-tubulin and play a part in a range of physiological processes including cell mitosis, shape maintenance, intracellular material transport, and signal transmission (Akhmanova and Steinmetz, 2015; Wang et al., 2022). Disruption of the dynamic balance of tubulin will interfere with the normal function of microtubules, lead to mitotic catastrophe and ultimately induce apoptosis (Liu et al., 2021b). Due to their important functions in cell division, microtubules have been considered a popular target for the development of anticancer drugs (Jordan and Wilson, 2004). Microtubule-targeting agents were found to bind to at least six different sites, among which inhibitors binding to the colchicine binding site have been always attracting considerable attention in anticancer therapy due to their advantages such as simple structure, broad therapeutic index, and significant ability to overcome clinically relevant multidrug resistance (Cermak et al., 2020; Wang et al., 2021b). Colchicine binding site inhibitors (CBSIs) exert their biological activities by inhibiting the important process of tubulin assembly, and consequently suppressing microtubule formation. It was reported that the colchicine binding site inhibitors have seven main pharmacophoric features, inculding one hydrogen bond donor, three hydrogen bond acceptors, one planar group, and two hydrophobic centers (Eissa et al., 2021; Hagras et al., 2021). In recent decades, a great deal of tubulin polymerization inhibitors with diverse backbones targeting the colchicine binding site, such as colchicine (**1**, Figure 1), combretastatin A-4 (CA-4, **2**, Figure 1), and *iso*combretastatin A-4 (*iso*CA-4, **3**, Figure 1) and NSC 676693 (**4**, Figure 1) have been investigated (Lisowski et al., 2004; Lu et al., 2012; Hamze et al., 2020).

NSC 676693 is a novel antimitotic compound based on the arylthienopyrrolizinone molecular skeleton. It has strong anticancer activity in human cancer cells with IC_50_ in the nanomolar range and it interacts with tubulin in the micromolar range. Since its discovery, NSC 676693 has been regarded as a promising lead compound for tubulin polymerization inhibitors. This interesting pharmacological profile, resulting from inhibition of tubulin polymerization, encouraged us to start structural modification of NSC 676693 which led to the development of more active antitumor drugs (Lisowski et al., 2004).

Carbazole fused heterocycle has recently drawn increasing interest as a privileged skeleton for the discovery of antitumor agents. Most interestingly, its derivatives, presented as compounds **5** and **6** (Figure 1), exerted outstanding antitumor potencies and anti-tubulin activities in the low nanomolar range (Bzeih et al., 2016; Naret et al., 2019). Based on the above findings, a stable fragment carbazole derivative was chosen to replace the B-ring of NSC 676693 through a bioisosterism strategy.

**FIGURE 2:**
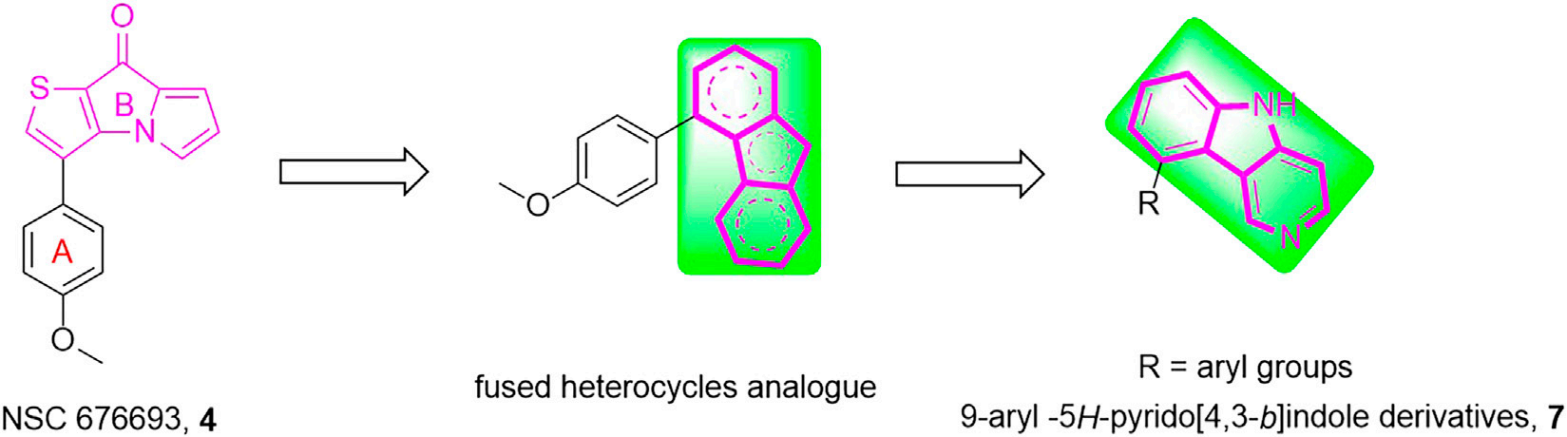
The rational design of target compounds.

Herein, a series of 9-aryl-5*H*-pyrido [4,3-*b*]indole derivatives (**7**, Figure 2) were designed and synthesized as anti-tubulin agents. To explore the structure-activity relationship (SAR) of NSC 676693 analogues, various substituents have been introduced in different positions of the A-ring. The preliminary tests of bioactivity *in vitro*, including antiproliferative activity, tubulin polymerization, immunofluorescence staining, cell cycle analysis, and apoptosis assay were performed to explore the preliminarily SAR and illuminate the pharmacologic mechanism. Additionally, molecular modeling was carried out to investigate the possible binding mode of target compounds.

## 2 Result and discussion

### 2.1 Chemistry

**SCHEME 1:**
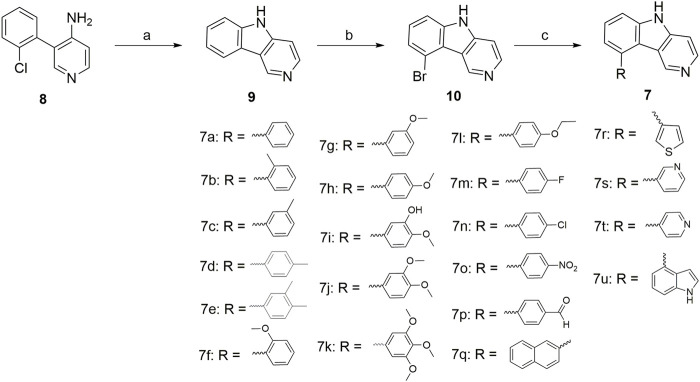
Reagents and conditions: (a) potassium tert-butoxide, dry DMSO, 130°C, 24 h; (b) *N*-bromosuccinimide (NBS), DMF, r.t., 12 h; (c) Substituted phenylboronic acid, Pd(PPh_3_)_4_, K_2_CO_3_, 1,4-dioxane/H_2_O = 3/1, N_2_ atmosphere, 126°C, 25 min, M.W.

The chemical synthesis route of 9-aryl-5*H*-pyrido[4,3-*b*]indole derivatives (**7a-7u**) was displayed in Scheme 1. Commercially available 3-(2-chlorophenyl)pyridin-4-amine (**8**) was used as starting material to react with potassium tert-butoxide to produce the 5*H*-pyrido[4,3-*b*]indole (**9**) (Akitake et al., 2021). Compound **9** further reacted with *N*-bromosuccinimide (NBS) in the presence of DMSO to give 9-bromo-5*H*-pyrido[4,3-*b*]indole (**10**) at room temperature (JEON et al., 2019). Finally, the target compounds **7a-7u** were generated by the Suzuki crosscoupling reaction between compound **10** and the corresponding phenylboronic acid (Li et al., 2020; Yang et al., 2020).

### 2.2 Biological evaluation

#### 2.2.1 Anti-proliferative activity

All 9-aryl-5*H*-pyrido[4,3-*b*]indole derivatives (**7a-7u**) were evaluated *in vitro* by MTT assay for anti-proliferative activities against a group of human cancer cell lines (gastric adenocarcinoma SGC-7901 cells, cervical carcinoma HeLa cells, and breast cancer MCF-7 cells), using CA-4 as the positive control.

Most of the compounds exhibited moderate anti-proliferative activity against the three tested cell lines with IC_50_ values in the micromolar range, as summarized in Table 1. In general, introducing aryl groups such as naphthalene (**7q**), thiophene (**7r**), pyridine (**7s** and **7t**), and indole (**7u**) into A-ring was not desirable. Moreover, **7a** with unsubstituted A-ring showed moderate activity, and the introduction of electron withdrawing groups on the A-ring, such as fluorine (**7m**), chlorine (**7n**), nitro (**7o**), and formyl (**7p**), led to a sharp decrease in the inhibitory activity. When electron donating groups, such as methyl (**7b**), methoxy (**7f**), 3-hydroxy-4-methoxy (**7i**), 3,4-dimethoxy (**7j**), and trimethoxy (**7k**), were introduced to the A-ring, resulted in maintenance or increase in antiproliferative activity. Among the compounds we designed, **7k** exhibited the most potent anticancer activity against HeLa cells, which was weaker cytotoxic than CA-4.

**TABLE 1:** Anti-proliferative activity of all target compounds.

Compounds	(IC50 ± SD, μM)^a^		
	HeLa	SGC-7901	MCF-7
**7a**	17.2 ± 3.1	15.6 ± 2.2	20.8 ± 3.1
**7b**	17.6 ± 1.7	18.3 ± 1.9	20.3 ± 2.9
**7c**	30.8 ± 3.5	>40	>40
**7d**	22.2 ± 3.2	19.6 ± 2.2	23.9 ± 1.9
**7e**	19.8 ± 3.0	28.9 ± 3.9	>40
**7f**	16.6 ± 1.6	17.9 ± 2.9	17.1 ± 4.1
**7g**	32.8 ± 3.2	28.0 ± 1.7	>40
**7h**	31.0 ± 4.1	33.2 ± 3.7	37.4 ± 4.8
**7i**	11.7 ± 2.2	15.3 ± 2.1	14.6 ± 3.6
**7j**	16.7 ± 3.3	17.4 ± 3.9	20.8 ± 3.5
**7k**	8.7 ± 1.3	9.3 ± 1.5	12.3 ± 2.3
**7l**	31.0 ± 4.3	>40	>40
**7m**	33.7 ± 2.6	35.1 ± 3.0	31.8 ± 1.9
**7n**	39.7 ± 1.3	>40	33.9 ± 2.6
**7o**	34.8 ± 2.2	37.6 ± 3.4	>40
**7p**	>40	35.1 ± 1.5	>40
**7q**	38.6 ± 2.7	>40	36.5 ± 1.8
**7r**	33.2 ± 4.0	>40	37.7 ± 2.9
**7s**	36.5 ± 2.9	38.2 ± 1.3	>40
**7t**	>40	>40	>40
**7u**	31.8 ± 3.0	36.2 ± 2.4	36.6 ± 3.2
**CA-4** ^b^	0.088 ± 0.009	0.11 ± 0.008	0.13 ± 0.012

a IC_50_: the half maximal inhibitory concentration.

b Used as positive controls.

#### 2.2.2 Effect on tubulin polymerization

**FIGURE 3:**
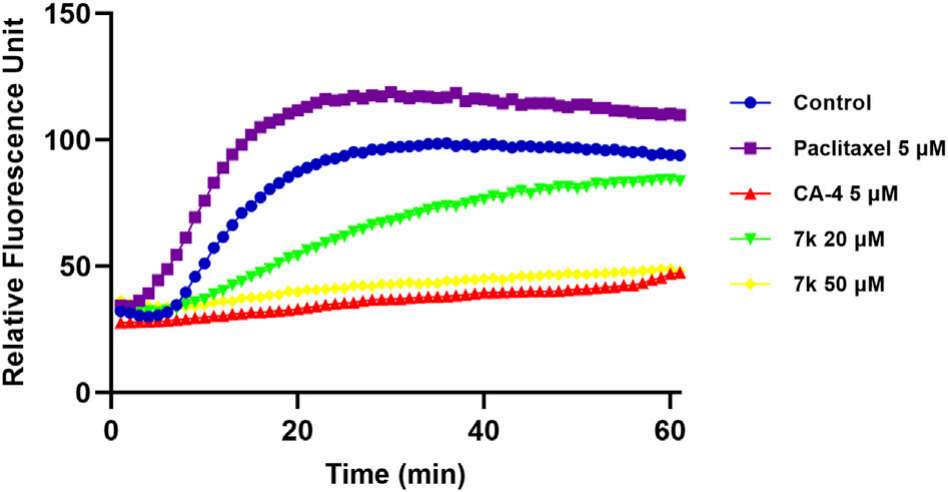
Effect of **7k** on tubulin polymerization. Tubulin had been pre-incubated for 1 min with **7k** at 20 μM, 50 μM, CA-4 at 5 μM, Paclitaxel at 5 μM, and vehicle DMSO at room temperature before GTP was added to start the tubulin polymerization reactions. The reaction was monitored continuously by measuring the absorbance at 340 nm every 1 min for 60 min at 37°C.

To verify the effect of target compounds on tubulin, the most potent compound **7k** was selected to assay its effect on tubulin polymerization, Paclitaxel and CA-4 were selected as negative and positive controls. The results were shown in Figure 3, **7k** and CA-4 exhibited remarkable inhibition against tubulin polymerization. In contrast, paclitaxel promoted tubulin polymerization. Therefore, the results suggested that **7k** was a tubulin polymerization inhibitor and interfered with tubulin polymerization in a dose-dependent manner.

#### 2.2.3 Immunofluorescence staining analysis

**FIGURE 4:**
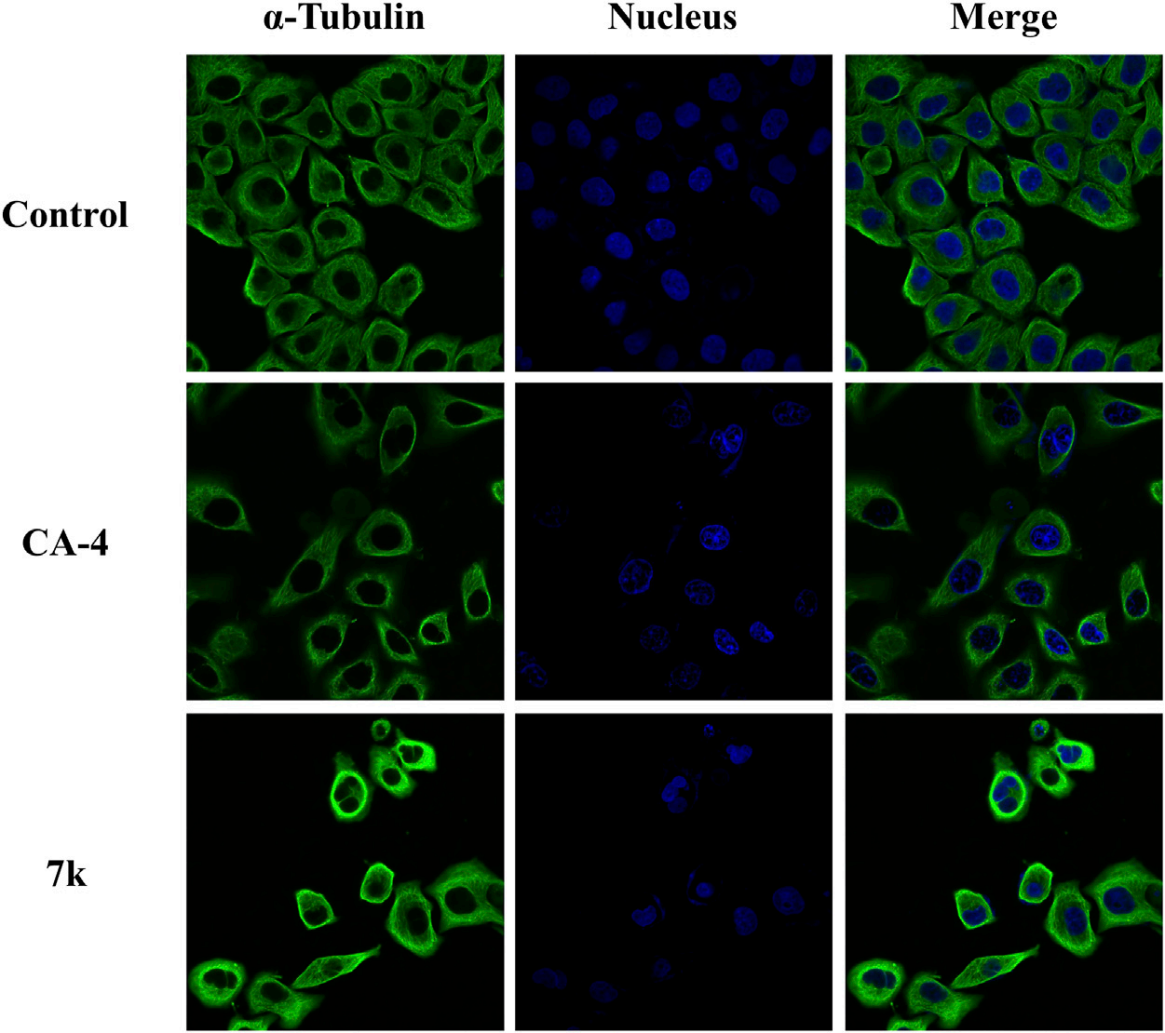
Effect of **7k** and CA-4 on the microtubule network of HeLa cells. After treatment with control (vehicle DMSO), CA-4 at 1-fold IC_50_ concentrations, and **7k** at 2-fold IC_50_ concentrations for 24 h, cells were stained with FITC-conjugated anti-*α*-tubulin antibody and DAPI. Microtubules and unassembled tubulin are shown in green, and the nucleus is shown in blue.

To examine the impact of compound **7k** on tumor cell microtubules, an immune stain study was carried out *via* confocal immunofluorescent microscopy. HeLa cells were incubated with **7k** at 2-fold IC_50_ concentrations and CA-4 at 1-fold IC_50_ concentrations for 24 h, respectively. As illustrated in Figure 4, HeLa cells without drug treatment displayed normal arrangement and organization. After treatment with indicated concentrations of **7k** or CA-4, microtubules showed irregular arrangement, became short, and the microtubule network showed a disruption. The results further confirmed that **7k** could act on tubulin to inhibit microtubule assembly and disrupt the cytoskeleton.

#### 2.2.4 Analysis of cell cycle

**FIGURE 5:**
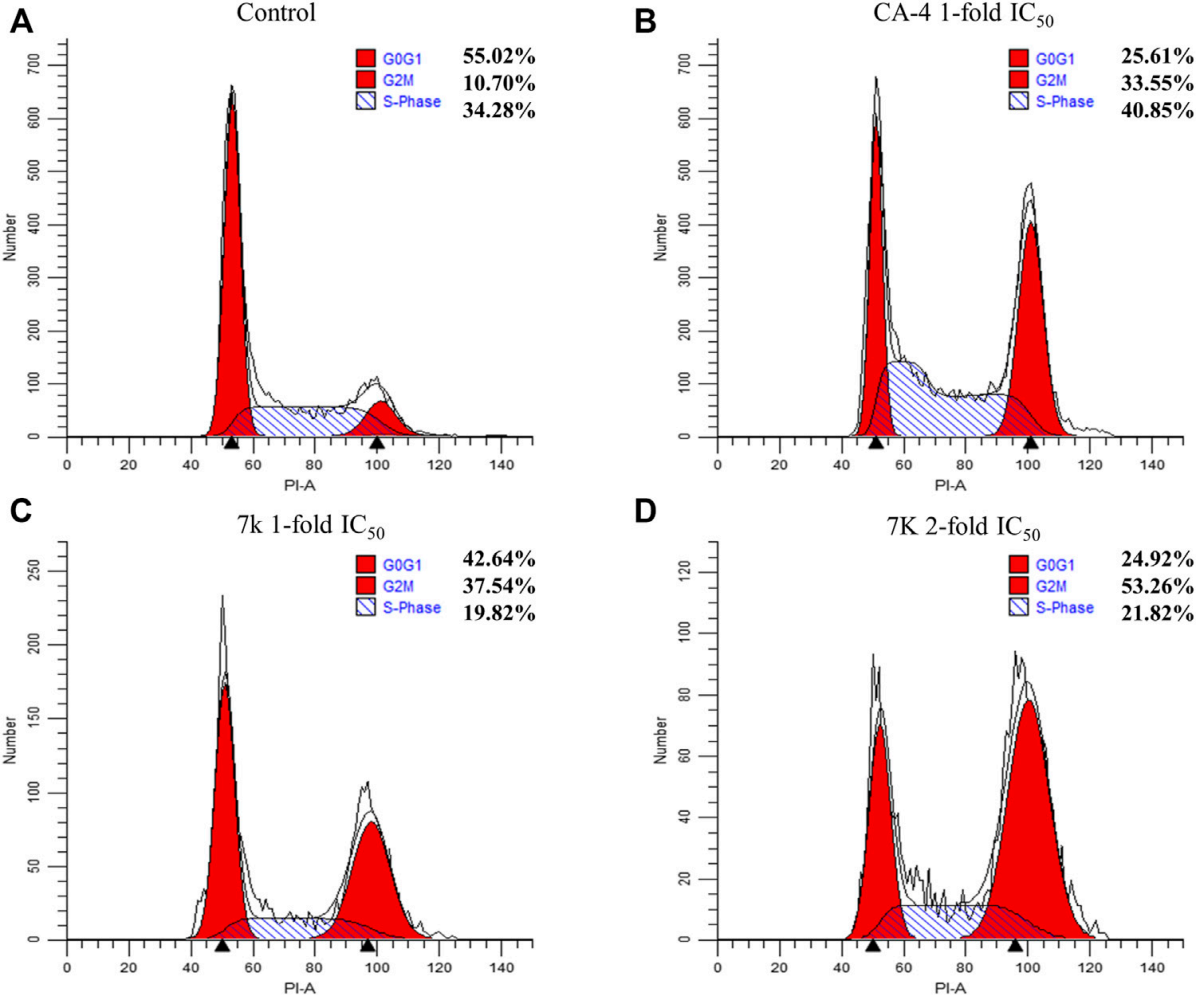
Effect of **7k** and CA-4 on HeLa cell cycle progress. Flow cytometry analysis of HeLa stained by propidium iodide and treated with different concentrations of **7k** for 24 h. **(A)** Control; **(B)** CA-4, 1-fold IC_50_; **(C) 7k**, 1-fold IC_50_; **(D) 7k**, 2-fold IC_50_.

To investigate whether compound **7k** could arrest cell cycle distribution, the cell cycle arrest assay was performed by flow cytometry. HeLa cells were treated with different concentrations of **7k** (1 and 2-fold IC_50_) and CA-4 (1-fold IC_50_) for 24 h. As presented in Figure 5, after treatment with specified concentrations of **7k**, the cell accumulation in the G2/M phase was 37% and 53% compared with 10% in the control group. The percentage of cells treated with CA-4 in the G2/M phase was 33%. The results indicated that analogue **7k** caused cell arrest at G2/M phase, which was a representative characteristic of tubulin polymerization inhibitors.

#### 2.2.5 Induction of cell apoptosis

**FIGURE 6:**
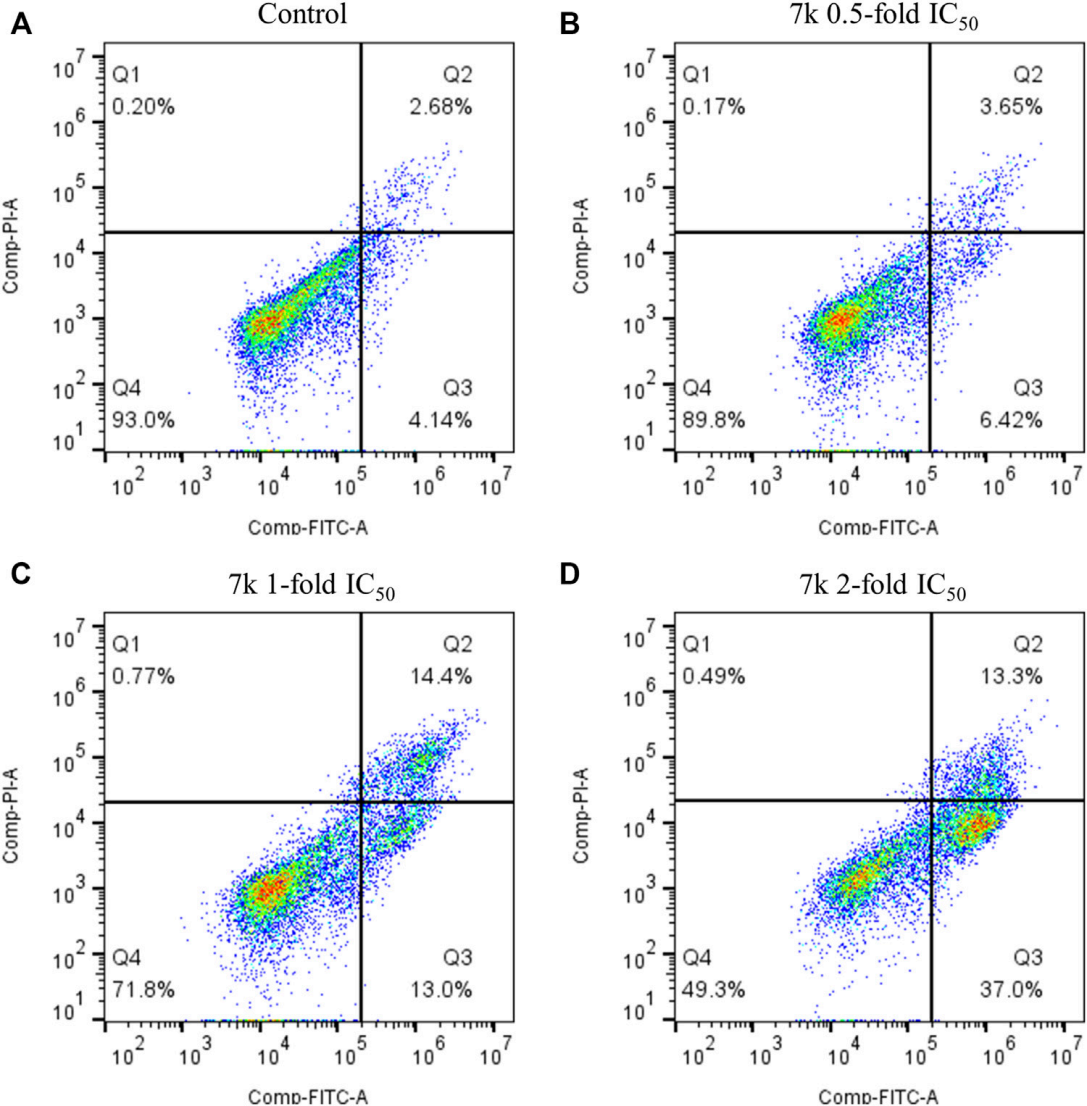
Effect of **7k** on HeLa cell apoptosis. Cells were harvested and stained with Annexin-V/PI for analysis after treatment with different concentrations of compound **7k** for 48 h. **(A)** Control; **(B) 7k**, 0.5-fold IC_50_; **(C) 7k**, 1-fold IC_50_; **(D) 7k**, 2-fold IC_50_.

To explore whether **7k** could induce apoptosis, we performed Annexin V-FITC/PI double staining assay. In this work, HeLa cells were grown with different concentrations of **7k** (0.5, 1, and 2-fold IC_50_) for 48 h. As demonstrated in Figure 6, percentages of total apoptotic cells from 7% (control) increased to 10, 27, and 50%, respectively. Hence, the results revealed that **7k** could indeed induce cell apoptosis in a dose-dependent manner.

#### 2.2.6 Molecular docking

**FIGURE 7:**
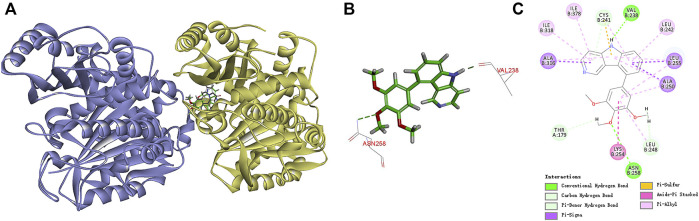
**(A)** Predicted modes for compound **7k** (green) and NSC 676693 (grey) binding in the colchicine binding site of tubulin (PDB: 5LYJ); **(B,C)** Docking conformation of compound **7k** in the colchicine binding site of tubulin.

To understand the possible binding mode of these newly synthesized compounds with the colchicine binding site on tubulin, a molecular modeling study of the most potent compound **7k** was carried out using Discovery Studio 3.0 software package. Docking studies revealed that compound **7k** occupied the colchicine binding site of *α*, *β*-tubulin and was mostly buried in the *β* subunit (Figure 7A). For **7k**, a hydrogen bond was formed between the oxygen atom of the methoxyl group and the residue of Asn*β*258. Additionally, the nitrogen atom of the 5*H*-pyrido[4,3-*b*]indole formed another hydrogen bond with the residue of Val*β*238. The results manifested that compound **7k** may bind to the colchicine binding site on tubulin.

## 3 Conclusion

In summary, a series of new 9-aryl-5*H*-pyrido[4,3-*b*]indole derivatives as tubulin polymerization inhibitors were designed and synthesized. Most of the tested compounds showed moderate antiproliferative activity. Among them, **7k** with 3,4,5-trimethoxyphenyl as the A-ring and 5*H*-pyrido[4,3-*b*]indole as the B-ring has the strongest activity against the HeLa cell line with IC_50_ values of 8.7 ± 1.3 μM and the SAR of tested compounds has been research in depth. From *in vitro* tubulin polymerization assay and immunofluorescence assay, **7k** could effectively inhibit the polymerization of tubulin and destroy the microtubule skeleton, which confirms that compound **7k** is a novel tubulin inhibitor. Further mechanism studies have shown that **7k** could effectively arrest cells in the G2/M phase, interfere with the mitotic process of tumor cells, and eventually cause apoptosis. Additionally, molecular docking study suggested that **7k** had high binding affinities for the colchicine binding pocket of tubulin. Our work reveals that 5*H*-pyrido[4,3-*b*]indole core may be used as the leading unit to develop novel tubulin polymerization inhibitors as potential anticancer agents.

## 4 Experimental section

### 4.1 Chemistry

#### 4.1.1 General material and method

All the reagents and solvents were obtained commercially and used without further purification. The microwave reactions were carried on the Discovery SP (CEM, Corporation, NC, United States). The progression of the reaction was monitored by TLC under UV light (wavelength: 254 nm and 365 nm). ^1^H (500 MHz) and ^13^C NMR (125 MHz) spectra were recorded with an Agilent ProPulse-500 (Agilent, Santa Clara, CA, United States) with DMSO-*d*_6_ as solvent at room temperature. Mass spectrometry (MS) was detected on an Agilent 1100-sl mass spectrometer equipped with an electrospray ionization source (Agilent, Santa Clara, CA, United States).

#### 4.1.2 General synthetic procedures for 5H-pyrido[4,3-*b*]indole (9)

To a round-bottom flask equipped with a magnetic stir bar, 3-(2-chlorophenyl)pyridin-4-amine **8**) (0.20 mmol), potassium tert-butoxide (1.0 mmol), and dry DMSO (5.0 ml) were added. The flask was evacuated and backfilled with nitrogen. The mixture was stirred at 130°C in an oil bath for 24 h. After the reaction was completed, the mixture was extracted with EtOAc (15 ml × 3). The organic layer was washed with brine and dried over Na_2_SO_4_. The filtrate was concentrated in vacuo and purified by silica gel column chromatography to give **9**.

#### 4.1.3 General synthetic procedures for 9-bromo-5H-pyrido[4,3-*b*]indole (10)

8.99 mmol of *N*-bromosuccinimide (NBS) was added in small portions to 7.49 mmol of the 5*H*-pyrido [4,3-*b*]indole (**9**) solution in 20 ml of DMF (20 ml) at room temperature. The reaction mixture was stirred at room temperature for 12 h and diluted with 20 ml of H_2_O. The aqueous layer was extracted therefrom by using EtOAc (30 ml × 3) and evaporated in vacuum. The residue was purified by column chromatography (5% MeOH in CH_2_Cl_2_) to generate **10**.

#### 4.1.4 General synthetic procedures for 9-aryl-5H-pyrido[4,3-*b*]indole derivatives (7)

A mixture of **10** (0.10 mmol), Pd(PPh_3_)_4_ (0.01 mmol), and K_2_CO_3_ (0.12 mmol), and substituted phenylboronic acid (0.11 mmol) in 1,4-dioxane/H_2_O (5 ml, 3:1) was degassed and purged with N_2_ for about three times. After stirring at irradiated in a microwave reactor for 25 min at 130°C (indicated by TLC) under N_2_ atmosphere, H_2_O (50 ml) was added to the reaction mixture and extracted with ethyl acetate (80 ml×3). The combined organics were washed with brine (10 ml × 3), dried over anhydrous Na_2_SO_4_, filtered, and concentrated under vacuum to give a residue, which was purified by column 300 chromatography using a mixture of petroleum ether and ethyl acetate (3:1) as an eluent to provide the target compounds **7**.

##### 4.1.4.1 9-Phenyl-5H-pyrido[4,3-b]indole (7a)

White solid; yield: 67%; ^1^H NMR (500 MHz, DMSO-*d*_6_) δ 13.12 (s, 1H), 8.48 (s, 1H), 7.81 (d, *J* = 6.2 Hz, 2H), 7.73 (d, *J* = 7.6 Hz, 2H), 7.67 (d, *J* = 7.4 Hz, 1H), 7.60 (t, *J* = 7.3 Hz, 2H), 7.56 (d, *J* = 8.5 Hz, 1H), 7.25 (d, *J* = 6.6 Hz, 2H); ^13^C NMR (125 MHz, DMSO-*d*_6_) δ 146.17, 141.49, 140.21, 138.65, 137.94, 137.66, 129.52 (2C), 128.97 (2C), 128.86, 128.80, 122.94, 119.52, 118.54, 112.03, 108.12. MS (ESI) m/z 245.0 [M + H]^+^, 267.0 [M + Na]^+^.

##### 4.1.4.2 9-(O-tolyl)-5H-pyrido[4,3-b]indole (7b)

White solid; yield: 52%; ^1^H NMR (500 MHz, DMSO-*d*_6_) δ 12.10 (s, 1H), 8.34 (s, 1H), 7.88 (s, 1H), 7.58 (td, *J* = 17.3, 8.2 Hz, 3H), 7.44 (d, *J* = 4.0 Hz, 2H), 7.36 (dt, *J* = 8.7, 4.2 Hz, 1H), 7.28 (d, *J* = 7.4 Hz, 1H), 7.09 (d, *J* = 7.0 Hz, 1H), 1.99 (s, 3H); ^13^C NMR (125 MHz, DMSO-*d*_6_) δ 144.65, 143.28, 141.63, 140.42, 140.25, 136.70, 135.76, 130.66, 129.38, 128.60, 127.56, 126.73, 121.41, 119.50, 111.11, 109.99, 107.22, 19.75. MS (ESI) m/z 259.0 [M + H]^+^.

##### 4.1.4.3 9-(M-tolyl)-5H-pyrido[4,3-b]indole (7c)

White solid; yield: 82%; ^1^H NMR (500 MHz, DMSO-d_6_) δ 12.36 (s, 1H), 8.49 (s, 3H), 7.63 (d, J = 8.1 Hz, 1H), 7.57 (t, J = 7.7 Hz, 1H), 7.46 (t, J = 7.4 Hz, 1H), 7.44-7.37 (m, 2H), 7.34 (d, J = 7.3 Hz, 1H), 7.16 (d, J = 7.2 Hz, 1H), 2.41 (s, 3H); ^13^C NMR (125 MHz, DMSO-d_6_) δ 144.91, 141.99, 140.88, 140.72, 138.58, 137.78, 129.61, 129.19, 129.11, 127.83, 126.75, 126.12, 122.03, 118.96, 118.80, 111.39, 109.99, 21.55. MS (ESI) m/z 259.1 [M + H]^+^, 281.0 [M + Na]^+^.

##### 4.1.4.4 9-(P-tolyl)-5H-pyrido [4,3-b]indole (7d)

White solid; yield: 86%; ^1^H NMR (500 MHz, DMSO-*d*_6_) δ 12.08 (s, 1H), 8.45 (d, *J* = 106.2 Hz, 2H), 7.57 (d, *J* = 8.0 Hz, 1H), 7.54-7.45 (m, 4H), 7.38 (d, *J* = 7.8 Hz, 2H), 7.09 (d, *J* = 7.2 Hz, 1H), 2.43 (s, 3H); ^13^C NMR (125 MHz, DMSO-*d*_6_) δ 144.42, 143.98, 143.00, 140.54, 138.18, 137.66, 137.50, 129.78 (2C), 128.99 (2C), 127.23, 123.50, 121.58, 118.90, 110.96, 107.02, 21.34. MS (ESI) m/z 259.0 [M + H]^+^, 281.0 [M + Na]^+^.

##### 4.1.4.5 9-(3,4-Dimethylphenyl)-5H-pyrido[4,3-b]indole (7e)

White solid; yield: 52%; ^1^H NMR (500 MHz, DMSO-*d*_6_) δ 12.51 (s, 1H), 8.57 (s, 1H), 8.41 (d, *J* = 4.4 Hz, 1H), 7.64 (dd, *J* = 10.6, 6.8 Hz, 2H), 7.58 (t, *J* = 7.7 Hz, 1H), 7.39 (s, 1H), 7.34 (s, 2H), 7.16 (d, *J* = 7.1 Hz, 1H), 2.34 (s, 3H), 2.32 (s, 3H); ^13^C NMR (125 MHz, DMSO-*d*_6_) δ 145.31, 141.27, 141.01, 140.38, 138.11, 137.89, 137.27, 136.59, 130.35, 130.04, 128.03, 126.34, 122.24, 119.66, 118.71, 111.30, 107.49, 19.89, 19.69. MS (ESI) m/z 273.1 [M + H]^+^.

##### 4.1.4.6 9-(2-Methoxyphenyl)-5H-pyrido[4,3-b]indole (7f)

White solid; yield: 53%; ^1^H NMR (500 MHz, DMSO-*d*_6_) δ 12.47 (s, 1H), 8.40 (s, 1H), 8.15 (s, 1H), 7.63 (d, *J* = 8.0 Hz, 2H), 7.60-7.55 (m, 1H), 7.53 (t, *J* = 7.2 Hz, 1H), 7.35 (dd, *J* = 7.4, 1.4 Hz, 1H), 7.25 (d, *J* = 8.3 Hz, 1H), 7.19-7.09 (m, 2H), 3.61 (s, 3H); ^13^C NMR (125 MHz, DMSO-*d*_6_) δ 156.82, 145.09, 141.12, 141.04, 140.47, 140.13, 134.15, 131.04, 130.30, 129.16, 127.80, 122.80, 121.33, 119.83, 111.94, 111.37, 107.42, 55.72. MS (ESI) m/z 275.0 [M + H]^+^, 297.0 [M + Na]^+^.

##### 4.1.4.7 9-(3-Methoxyphenyl)-5*H*-pyrido[4,3-*b*]indole (7g)

White solid; yield: 93%; 1H NMR (500 MHz, DMSO-d6) δ 12.57 (s, 1H), 8.50 (d, J = 79.3 Hz, 2H), 7.65 (d, J = 7.9 Hz, 2H), 7.59 (td, J = 7.6, 2.7 Hz, 1H), 7.49 (td, J = 7.8, 2.9 Hz, 1H), 7.19 (t, J = 6.9 Hz, 2H), 7.15 (s, 1H), 7.09 (d, J = 8.2 Hz, 1H), 3.81 (s, 3H); 13C NMR (125 MHz, DMSO-*d*_6_) δ 160.03, 145.29, 142.05, 141.46, 140.99, 140.60, 137.54, 130.48, 128.00, 122.17, 121.25, 118.67, 114.43, 114.20, 111.65, 109.99, 107.56, 55.67. MS (ESI) m/z 275.0 [M + H]^+^.

##### 4.1.4.8 9-(4-Methoxyphenyl)-5H-pyrido[4,3-b]indole (7h)

White solid; yield: 93%; 1H NMR (500 MHz, DMSO-d6) δ 12.14 (s, 1H), 8.47 (d, J = 118.4 Hz, 2H), 7.59-7.46 (m, 5H), 7.13 (d, J = 8.6 Hz, 2H), 7.10 (d, J = 7.1 Hz, 1H), 3.86 (s, 3H); 13C NMR (125 MHz, DMSO-*d*_6_) δ 159.52, 144.55, 143.66, 142.67, 140.61, 137.31, 133.22, 130.29 (2C), 127.33, 121.74, 119.65, 118.94, 114.65 (2C), 110.83, 107.01, 55.68. MS (ESI) m/z 275.1 [M + H]^+^.

##### 4.1.4.9 2-Methoxy-5-(5H-pyrido[4,3-b]indol-9-yl)phenol (7i)

White solid; yield: 97%; 1H NMR (500 MHz, DMSO-d6) δ 12.27 (s, 1H), 9.33 (s, 1H), 8.66 (s, 1H), 8.36 (s, 1H), 7.59-7.47 (m, 3H), 7.09 (dd, J = 7.6, 3.7 Hz, 2H), 7.06 (d, J = 2.0 Hz, 1H), 6.98 (dd, J = 8.1, 2.0 Hz, 1H), 3.86 (s, 3H); 13C NMR (125 MHz, DMSO-*d*_6_) δ 148.01, 147.15, 144.73, 142.99, 142.28, 140.68, 137.69, 133.57, 127.44, 121.67, 119.74, 119.63, 118.82, 116.35, 112.95, 110.81, 107.08, 56.15. MS (ESI) m/z 291.0 [M + H]^+^, 313.0 [M + Na]^+^.

##### 4.1.4.10 9-(3,4-Dimethoxyphenyl)-5H-pyrido[4,3-b]indole (7j)

White solid; yield: 85%; ^1^H NMR (500 MHz, DMSO-d6) δ 12.97 (s, 1H), 8.70 (s, 1H), 8.47 (s, 1H), 7.76 (d, J = 5.8 Hz, 1H), 7.67 (d, J = 8.0 Hz, 1H), 7.64-7.58 (m, 1H), 7.24 (d, J = 7.2 Hz, 1H), 7.17 (dd, J = 20.3, 10.0 Hz, 3H), 3.85 (s, 3H), 3.78 (s, 3H); ^13^C NMR (125 MHz, DMSO-*d*_6_) δ 149.42, 149.25, 145.84, 141.39, 138.67, 137.91, 132.75, 128.51, 122.78, 121.18 (2C), 119.67, 118.66, 112.67, 112.61, 111.48, 107.87, 56.08 (2C). MS (ESI) m/z 305.0 [M + H]^+^, 327.0 [M + Na]^+^.

##### 4.1.4.11 9-(3,4,5-Trimethoxyphenyl)-5H-pyrido[4,3-b]indole (7k)

White solid; yield: 96%; ^1^H NMR (500 MHz, DMSO-*d*_6_) δ 12.37 (s, 1H), 8.69 (s, 1H), 8.39 (d, *J* = 5.5 Hz, 1H), 7.61 (d, *J* = 8.1 Hz, 1H), 7.59-7.50 (m, 2H), 7.21 (d, *J* = 7.3 Hz, 1H), 6.90 (s, 2H), 3.79 (s, 6H), 3.77 (s, 3H); ^13^C NMR (125 MHz, DMSO-*d*_6_) δ 153.56 (2C), 144.85, 142.91, 142.16, 140.74, 137.70, 137.62, 136.44, 127.45, 121.82, 119.52, 118.75, 111.29, 107.22, 106.47 (2C), 60.67, 56.47 (2C). MS (ESI) m/z 335.1 [M + H]^+^, 357.0 [M + Na]^+^.

##### 4.1.4.12 9-(4-Ethoxyphenyl)-5H-pyrido[4,3-b]indole (7l)

White solid; yield: 94%; ^1^H NMR (500 MHz, DMSO-*d*_6_) δ 12.09 (s, 1H), 8.47 (d, *J* = 122.7 Hz, 2H), 7.55 (d, *J* = 8.0 Hz, 1H), 7.54-7.46 (m, 4H), 7.10 (dd, *J* = 9.9, 8.0 Hz, 3H), 4.12 (q, *J* = 6.9 Hz, 2H), 1.38 (t, *J* = 6.9 Hz, 3H); ^13^C NMR (125 MHz, DMSO-*d*_6_) δ 158.78, 144.44, 143.92, 142.92, 140.56, 137.32, 133.14, 130.29 (2C), 127.23, 121.63, 119.60, 118.98, 115.05 (2C), 110.76, 106.91, 63.60, 15.18. MS (ESI) m/z 289.1 [M + H]^+^, 311.0 [M + Na]^+^.

##### 4.1.4.13 9-(4-Fluorophenyl)-5H-pyrido[4,3-b]indole (7m)

White solid; yield: 51%; ^1^H NMR (500 MHz, DMSO-*d*_6_) δ 12.12 (s, 1H), 8.44 (d, *J* = 71.4 Hz, 2H), 7.65 (t, *J* = 6.0 Hz, 2H), 7.63-7.58 (m, 1H), 7.58-7.47 (m, 2H), 7.41 (t, *J* = 8.5 Hz, 2H), 7.17-7.08 (m, 1H); ^13^C NMR (125 MHz, DMSO-*d*_6_) δ 162.43 (d, *J* = 244.6 Hz), 144.62, 143.65, 142.43, 140.59, 137.35, 136.41, 131.17 (d, *J* = 8.2 Hz, 2C), 127.42, 121.86, 119.46, 118.88, 116.16 (d, *J* = 21.4 Hz, 2C), 111.37, 107.14. MS (ESI) m/z 263.0 [M + H]^+^, 285.1 [M + Na]^+^.

##### 4.1.4.14 9-(4-Chlorophenyl)-5H-pyrido[4,3-b]indole (7n)

White solid; yield: 56%; ^1^H NMR (500 MHz, DMSO-*d*_6_) δ 12.29 (s, 1H), 8.46 (d, *J* = 76.3 Hz, 2H), 7.68-7.60 (m, 5H), 7.54 (t, *J* = 7.5 Hz, 2H), 7.14 (t, *J* = 6.4 Hz, 1H); ^13^C NMR (125 MHz, DMSO-*d*_6_) δ 144.70, 143.49, 142.23, 140.68, 139.80, 136.11, 133.23, 131.00 (2C), 129.33 (2C), 128.94, 127.50, 121.81, 118.64, 111.64, 107.22. MS (ESI) m/z 279.0 [M + H]^+^, 301.0 [M + Na]^+^.

##### 4.1.4.15 9-(4-Nitrophenyl)-5H-pyrido[4,3-b]indole (7o)

Yellow solid; yield: 53%; ^1^H NMR (500 MHz, DMSO-*d*_6_) δ 12.56 (s, 1H), 8.59 (s, 1H), 8.43 (d, *J* = 8.7 Hz, 3H), 7.95 (d, *J* = 8.6 Hz, 2H), 7.73 (d, *J* = 8.1 Hz, 1H), 7.64 (t, *J* = 7.7 Hz, 2H), 7.25 (d, *J* = 7.2 Hz, 1H); ^13^C NMR (125 MHz, DMSO-*d*_6_) δ 147.65, 147.60, 145.04, 142.86, 141.64, 140.90, 135.26, 130.57, 127.84 (2C), 124.59, 122.18 (2C), 118.33, 112.64, 109.99, 107.50. MS (ESI) m/z 290.0 [M + H]^+^.

##### 4.1.4.16 4-(5H-pyrido[4,3-b]indol-9-yl)benzaldehyde (7p)

Yellow solid; yield:52%; ^1^H NMR (500 MHz, DMSO-*d*_6_) δ 12.22 (s, 1H), 10.14 (s, 1H), 8.54 (s, 1H), 8.37 (s, 1H), 8.12 (d, *J* = 8.0 Hz, 2H), 7.88 (d, *J* = 7.8 Hz, 2H), 7.66 (d, *J* = 8.1 Hz, 1H), 7.58 (t, *J* = 7.7 Hz, 1H), 7.53 (s, 1H), 7.20 (d, *J* = 7.1 Hz, 1H); 13C NMR (125 MHz, DMSO-*d*_6_) δ 193.34, 147.07, 144.61, 144.01, 142.74, 140.64, 136.18, 136.08 (2C), 130.49 (2C), 130.02, 128.72, 127.43, 121.74, 118.47, 112.03, 107.17. MS (ESI) m/z 273.0 [M + H]^+^.

##### 4.1.4.17 9-(Naphthalen-2-yl)-5H-pyrido[4,3-b]indole (7q)

White solid; yield:63%; ^1^H NMR (500 MHz, DMSO-*d*_6_) δ 12.49 (s, 1H), 8.46 (d, *J* = 51.4 Hz, 2H), 7.70-7.61 (m, 4H), 7.62-7.58 (m, 2H), 7.61-7.55 (m, 3H), 7.53 (t, *J* = 7.1 Hz, 1H), 7.18 (d, *J* = 7.2 Hz, 1H); ^13^C NMR (125 MHz, DMSO-*d*_6_) δ 145.20, 141.81, 140.94 (2C), 140.80, 140.71, 137.68, 129.36 (2C), 129.24, 129.04 (2C), 128.56, 127.96, 122.19, 119.54, 119.48, 118.72, 111.53, 107.51, 107.48. MS (ESI) m/z 317.1 [M + Na]^+^.

##### 4.1.4.18 9-(Thiophen-3-yl)-5H-pyrido[4,3-b]indole (7r)

Yellow solid; yield: 92%; ^1^H NMR (500 MHz, DMSO-*d*_6_) δ 12.46 (s, 1H), 8.71 (s, 1H), 8.42 (s, 1H), 7.80 (dd, *J* = 4.4, 2.5 Hz, 2H), 7.62 (d, *J* = 7.5 Hz, 2H), 7.59-7.50 (m, 1H), 7.43 (dd, *J* = 4.2, 2.0 Hz, 1H), 7.22 (d, *J* = 7.2 Hz, 1H); ^13^C NMR (125 MHz, DMSO-*d*_6_) δ 145.06, 141.99, 141.18, 140.90, 132.53, 128.87, 127.76, 127.54, 124.23, 122.25, 119.59, 118.99, 111.54, 109.98, 107.39. MS (ESI) m/z 251.0 [M + H]^+^.

##### 4.1.4.19 9-(Pyridin-3-yl)-5H-pyrido[4,3-b]indole (7s)

White solid; yield: 76%; ^1^H NMR (500 MHz, DMSO-*d*_6_) δ 12.38 (s, 1H), 8.83 (s, 1H), 8.74 (d, *J* = 4.3 Hz, 1H), 8.42 (d, *J* = 36.6 Hz, 2H), 8.08 (d, *J* = 7.8 Hz, 1H), 7.67 (d, *J* = 8.1 Hz, 1H), 7.65-7.51 (m, 3H), 7.19 (d, *J* = 7.3 Hz, 1H); ^13^C NMR (125 MHz, DMSO-*d*_6_) δ 149.62, 149.52, 144.77, 143.48, 141.92, 140.71, 136.75, 136.60, 133.83, 127.61, 124.31, 122.19, 119.34, 118.90, 112.03, 107.31. MS (ESI) m/z 246.0 [M + H]^+^, 268.0 [M + Na]^+^.

##### 4.1.4.20 9-(Pyridin-4-yl)-5H-pyrido[4,3-b]indole (7t)

White solid; yield: 86%; ^1^H NMR (500 MHz, DMSO-*d*_6_) δ 12.25 (s, 1H), 8.77 (d, *J* = 5.4 Hz, 2H), 8.59 (s, 1H), 8.38 (s, 1H), 7.69-7.63 (m, 3H), 7.57 (t, *J* = 7.7 Hz, 1H), 7.53 (d, *J* = 5.5 Hz, 1H), 7.17 (d, *J* = 7.3 Hz, 1H); ^13^C NMR (125 MHz, DMSO-*d*_6_) δ 150.61 (2C), 148.73, 144.55, 144.36, 142.95, 140.61, 134.56, 127.38, 124.19 (2C), 121.55, 119.05, 118.18, 112.34, 107.18. MS (ESI) m/z 246.0 [M + H]^+^.

##### 4.1.4.21 9-(1H-indol-4-yl)-5H-pyrido[4,3-b]indole (7u)

White solid; yield: 93%; ^1^H NMR (500 MHz, DMSO-*d*_6_) δ 12.61 (s, 1H), 11.46 (s, 1H), 8.34 (d, *J* = 5.9 Hz, 1H), 7.93 (s, 1H), 7.68 (d, *J* = 7.9 Hz, 1H), 7.62 (t, *J* = 6.4 Hz, 2H), 7.58 (d, *J* = 8.1 Hz, 1H), 7.38-7.23 (m, 3H), 7.18 (d, *J* = 7.0 Hz, 1H), 5.92 (s, 1H); ^13^C NMR (125 MHz, DMSO-*d*_6_) δ 145.33, 141.01, 140.84, 140.80, 136.90, 136.50, 132.27, 127.92, 126.56, 126.42, 122.51, 121.65, 119.55, 119.53, 119.24, 111.95, 111.24, 107.34, 100.95. MS (ESI) m/z 284.0 [M + H]^+^, 306.1 [M + Na]^+^.

### 4.2 Biological evaluation

#### 4.2.1 Cell culture

Human gastric adenocarcinoma SGC-7901 cells, human cervical carcinoma HeLa cells, and human breast cancer MCF-7 cells were grown in DMEM medium supplemented with 10% fetal bovine serum (FBS), 1% penicillin-streptomycin solution. All cells were cultured at 37°C in a humidified atmosphere with 5% CO_2_.

#### 4.2.2 Anti-proliferative activity assay

The standard MTT assay was used to detect the anti-proliferative activity of all target compounds and CA-4, as described below (Tian et al., 2019). Firstly, depending on the growth rate of the cell line, the cells were inoculated in 96-well plates at 2000–5,000 per well. After incubation for 24 h, the culture medium was changed and the cells were exposed to various concentrations of the tested compounds for 72 h. Then, the medium containing the test compound was replaced with a fresh medium containing 5 mg/ml MTT, and plates were incubated in dark at 37°C for a further 4 h. Subsequently, the medium containing MTT was removed and 150 μL of DMSO was poured into each well to dissolve the resulting purple formazan crystals. The absorbance of the solution was measured at a wavelength of 490 nm by a Victor Nivo 3S microplate reader (PerkinElmer, United States). Finally, the growth inhibitory effects were represented as IC_50_ values which were calculated with GraphPad Prism 8.

#### 4.2.3 Tubulin polymerization assay

Tubulin polymerization assay for target compound **7k** was conducted in 96-well plates using the reagents described in the kit manufacturer (Cytoskeleton, Cat. #BK011P) (Wang et al., 2021a). The tubulin reaction mixture was composed of purified tubulin, 1 mM GTP, 20% glycerol, and PEM buffer (80 mM PIPES, 0.5 mM EGTA, 2 mM MgCl_2_). Firstly, 5 μL of the test compound was added to a 96-well plate and then warmed to 37°C for 1 min. 50 μL of the tubulin reaction mixture was added as specified to start the reaction. The fluorescence intensity was monitored for 60 min at 37°C using a Synergy Neo2 microplate reader (BioTek, United States).

#### 4.2.4 Immunofluorescence staining analysis

The effect of target compounds on the microtubule network was observed by immunofluorescence staining experiments (Wen et al., 2015). 3 × 10^5^ HeLa cells per well were seeded on slides and incubated overnight at 37°C in the bottom of a six-well plate. The cells were treated with **7k**, CA-4, and vehicle control (0.1% DMSO). After 24 h, the control and treated cells were fixed with pre-chilled 4% formaldehyde for 20 min, permeabilized with 0.5% Triton X-100 for 10 min, and washed three times with PBST. 3% bovine serum albumin (BSA) was used to block for 1 h and then removed. After this, *α*-tubulin antibody (1:100) (Santa Cruz, CA) was added to the slides and incubated for 3 h. Then, cells were washed three times with PBST to remove the unbound primary antibody. Next, the slides were incubated with FITC-conjugated secondary antibody for 1 h at 37°C, and the nuclei were stained with DAPI. Cells were washed with PBST, and mounting medium was added. Finally, the image results were presented by confocal microscopy (Nikon, Japan).

#### 4.2.5 Cell cycle distribution assay

The effect of target compounds on cell cycle phase distribution was analyzed by flow cytometry (Yang et al., 2020). HeLa cells were inoculated at 3 × 10^5^ cells/well in 6-well plates. After overnight adherence, cells were exposed to various concentrations of **7k**, CA-4, and vehicle control (0.1% DMSO) for 24 h. Subsequently, treated cells were collected by centrifugation and fixed with 75% ice-cold ethanol at 4°C overnight. The cells were then washed with PBS, incubated with 50 mg/ml of RNase at 37°C for 30 min, and stained with PI in the dark for 15 min at 4°C. Cell cycle distribution was finally analyzed with CytoFLEX (Beckman Coulter, United States) and the percentage of each phase of the cell cycle was calculated using ModfitLT 5.0 software.

#### 4.2.6 Cell apoptosis analysis

To investigate whether the target compound can induce apoptosis, an Annexin Van -FITC/PI experiment was carried out (Huo et al., 2021). HeLa cells were grown in 6-well plates (3 × 10^5^ cells/well) and incubated with various concentrations of **7k** or vehicle control (0.1% DMSO) for 48 h. Subsequently, cells were harvested by centrifugation, washed with PBS, and resuspended in binding buffer. Then, 10 μL of PI Staining Solution and 5 μL of Annexin V-FITC were added to the cell suspension for 15 min at room temperature in the dark. Finally, the samples were detected by a CytoFLEX (Beckman Coulter, United States) flow cytometer and the percentage of apoptotic cells was calculated using Flowjo 10.8 software.

#### 4.2.7 Molecular docking studies

The molecular docking was explored using the default settings of the Accelrys Discovery Studio 3.0 software package, as we previously reported. The crystal structure of tubulin in complex with colchicine (PDB: 5LYJ) was taken from the RCSB protein database (Liu et al., 2021a). According to the default settings of the CDOCKER protocol, compound **7k** was docked to the active site and its binding mode was explored.

## Data Availability

The original contributions presented in the study are included in the article/Supplementary Material, further inquiries can be directed to the corresponding authors.

## References

[B1] Akhmanova A. Steinmetz M. O. (2015). Control of microtubule organization and dynamics: Two ends in the limelight. Nat. Rev. Mol. Cell Biol. 16 (12), 711–726. 10.1038/nrm4084 26562752

[B2] Akitake M. Noda S. Miyoshi K. Sonoda M. Tanimori S. (2021). Access to gamma-carbolines: Synthesis of isocryptolepine. J. Org. Chem. 86 (24), 17727–17737. 10.1021/acs.joc.1c02026 34866396

[B3] Bzeih T. Naret T. Hachem A. Jaber N. Khalaf A. Bignon J. (2016). A general synthesis of arylindoles and (1-arylvinyl)carbazoles via a one-pot reaction from N-tosylhydrazones and 2-nitro-haloarenes and their potential application to colon cancer. Chem. Commun. 52 (88), 13027–13030. 10.1039/c6cc07666a 27752657

[B4] Cermak V. Dostal V. Jelinek M. Libusova L. Kovar J. Rosel D. (2020). Microtubule-targeting agents and their impact on cancer treatment. Eur. J. Cell Biol. 99 (4), 151075. 10.1016/j.ejcb.2020.151075 32414588

[B5] Eissa I. H. Dahab M. A. Ibrahim M. K. Alsaif N. A. Alanazi A. Z. Eissa S. I. (2021). Design and discovery of new antiproliferative 1 2 4-triazin-3(2H)-ones as tubulin polymerization inhibitors targeting colchicine binding site. Bioorg. Chem. 112, 104965. 10.1016/j.bioorg.2021.104965 34020238

[B6] Hagras M. El Deeb M. A. Elzahabi H. S. A. Elkaeed E. B. Mehany A. B. M. Eissa I. H. (2021). Discovery of new quinolines as potent colchicine binding site inhibitors: Design, synthesis, docking studies, and anti-proliferative evaluation. J. Enzyme Inhibition Med. Chem. 36 (1), 640–658. 10.1080/14756366.2021.1883598 PMC7889231 33588683

[B7] Hamze A. Alami M. Provot O. (2020). Developments of isoCombretastatin A-4 derivatives as highly cytotoxic agents. Eur. J. Med. Chem. 190, 112110. 10.1016/j.ejmech.2020.112110 32061961

[B8] Huo X. S. Jian X. E. Ou-Yang J. Chen L. Yang F. Lv D. X. (2021). Discovery of highly potent tubulin polymerization inhibitors: Design, synthesis, and structure-activity relationships of novel 2, 7-diaryl-[1, 2, 4]triazolo[1, 5-a]pyrimidines. Eur. J. Med. Chem. 220, 113449. 10.1016/j.ejmech.2021.113449 33895499

[B9] Jeon S. Hiroshi M. Yeonsook C. Myungsun S. Sooghang I. Wonchul L. (2019). Organic light-emitting device. Washington, DC U.S: Patent and Trademark Office.

[B10] Jordan M. A. Wilson L. (2004). Microtubules as a target for anticancer drugs. Nat. Rev. Cancer 4 (4), 253–265. 10.1038/nrc1317 15057285

[B11] Li G. Wang Y. X. Li L. Ren Y. C. Deng X. Liu J. (2020). Design, synthesis, and bioevaluation of pyrazolo[1, 5-a]pyrimidine derivatives as tubulin polymerization inhibitors targeting the colchicine binding site with potent anticancer activities. Eur. J. Med. Chem. 202, 112519. 10.1016/j.ejmech.2020.112519 32650183

[B12] Lisowski V. Leonce S. Kraus-Berthier L. Santos J. S. D. Pierre A. Atassi G. (2004). Design, synthesis, and evaluation of novel thienopyrrolizinones as antitubulin agents. J. Med. Chem. 47 (6), 1448–1464. 10.1021/jm030961z 14998333

[B13] Liu R. L. Huang M. X. Zhang S. Li L. Li M. Sun J. (2021a). Design, synthesis and bioevaluation of 6-aryl-1-(3, 4, 5-trimethoxyphenyl)-1H-benzo[d]imidazoles as tubulin polymerization inhibitors. Eur. J. Med. Chem. 226, 113826. 10.1016/j.ejmech.2021.113826 34571171

[B14] Liu R. L. Zhang S. Huang M. X. Guo Z. P. Li L. Li M. (2021b). Design, synthesis and bioevaluation of 2, 7-diaryl-pyrazolo[1, 5-a]pyrimidines as tubulin polymerization inhibitors. Bioorg. Chem. 115, 105220. 10.1016/j.bioorg.2021.105220 34352709

[B15] Lu Y. Chen J. J. Xiao M. Li W. Miller D. D. (2012). An overview of tubulin inhibitors that interact with the colchicine binding site. Pharm. Res. 29 (11), 2943–2971. 10.1007/s11095-012-0828-z 22814904 PMC3667160

[B16] Naret T. Khelifi I. Provot O. Bignon J. Levaique H. Dubois J. (2019). 1, 1-diheterocyclic ethylenes derived from quinaldine and carbazole as new tubulin-polymerization inhibitors: Synthesis, metabolism, and biological evaluation. J. Med. Chem. 62 (4), 1902–1916. 10.1021/acs.jmedchem.8b01386 30525602

[B17] Tian C. Chen X. Z. Zhang Z. L. Wang X. W. Liu J. Y. (2019). Design and synthesis of (2-(phenylamino)thieno[3, 2-d]pyrimidin-4-yl)(3, 4, 5-trimethoxyphenyl)methanone analogues as potent anti-tubulin polymerization agents. Eur. J. Med. Chem. 183, 111679. 10.1016/j.ejmech.2019.111679 31541870

[B18] Wang C. Wang Z. Y. Gao M. H. Li Y. L. Zhang Y. J. Bao K. (2021a). Design, synthesis and anticancer activity of 5-aryl-4-(4-arylpiperazine-1-carbonyl)-1, 2, 3-thiadiazoles as microtubule-destabilizing agents. Bioorg. Chem. 106, 104199. 10.1016/j.bioorg.2020.104199 33317837

[B19] Wang C. Zhang Y. J. Wang Z. Y. Li Y. L. Guan Q. Xing D. M. (2022). Design, synthesis, and biological evaluation of biotinylated colchicine derivatives as potential antitumor agents. J. Enzyme Inhibition Med. Chem. 37 (1), 417–426. 10.1080/14756366.2021.2013832 PMC8725855 34915785

[B20] Wang C. Zhang Y. J. Wu Y. D. Xing D. M. (2021b). Developments of CRBN-based PROTACs as potential therapeutic agents. Eur. J. Med. Chem. 225, 113749. 10.1016/j.ejmech.2021.113749 34411892

[B21] Wen Z. Y. Xu J. W. Wang Z. W. Qi H. Xu Q. L. Bai Z. S. (2015). 3-(3, 4, 5-Trimethoxyphenylselenyl)-1H-indoles and their selenoxides as combretastatin A-4 analogs: Microwave-assisted synthesis and biological evaluation. Eur. J. Med. Chem. 90, 184–194. 10.1016/j.ejmech.2014.11.024 25461319

[B22] Yang F. Jian X. E. Diao P. C. Huo X. S. You W. W. Zhao P. L. (2020). Synthesis, and biological evaluation of 3, 6-diaryl-[1, 2, 4]triazolo[4, 3-a] pyridine analogues as new potent tubulin polymerization inhibitors. Eur. J. Med. Chem. 204, 112625. 10.1016/j.ejmech.2020.112625 32717486

