# Supplementary material for: Design, synthesis and biological evaluation of 9-aryl-5H-pyrido[4,3-b]indole derivatives as potential tubulin polymerization inhibitors
Source: Front Chem. 2022 Sep 15;10:1004835. doi: 10.3389/fchem.2022.1004835 (PMC9520531; doi:10.3389/fchem.2022.1004835)
Supplement: Supplementary file 1 [file DataSheet1.pdf]

Design, synthesis and biological evaluation of 9-aryl-5*H*-pyrido[4,3-*b*]indole derivatives as potential tubulin polymerization inhibitors

Lingyu Shi[1,2], Shanbo Yang[1,2], Jing Chang[1,2], Yujing Zhang[3], Wenjing Liu[1,2], Jun Zeng[1,2], Jingsen Meng[1,2], Renshuai Zhang[1,2], Chao Wang[1,2*], Dongming Xing[1,2,4*]

[1]Cancer Institute, The Affiliated Hospital of Qingdao University and School of Basic Medicine, Qingdao University, Qingdao, China
[2]Qingdao Cancer Institute, Qingdao, China
[3]The Affiliated Cardiovascular Hospital of Qingdao University, Qingdao University, Qingdao, China
[4]School of Life Sciences, Tsinghua University, Beijing, China

*** Correspondence:**
Chao Wang
wangchao20086925@126.com
Dongming Xing
xdm_tsinghua@163.com

Contents: MS, ^{1}H-NMR, and ^{13}C-NMR spectrum of all target compounds.

*9-Phenyl-5H-pyrido[4,3-b]indole (**7a**)*

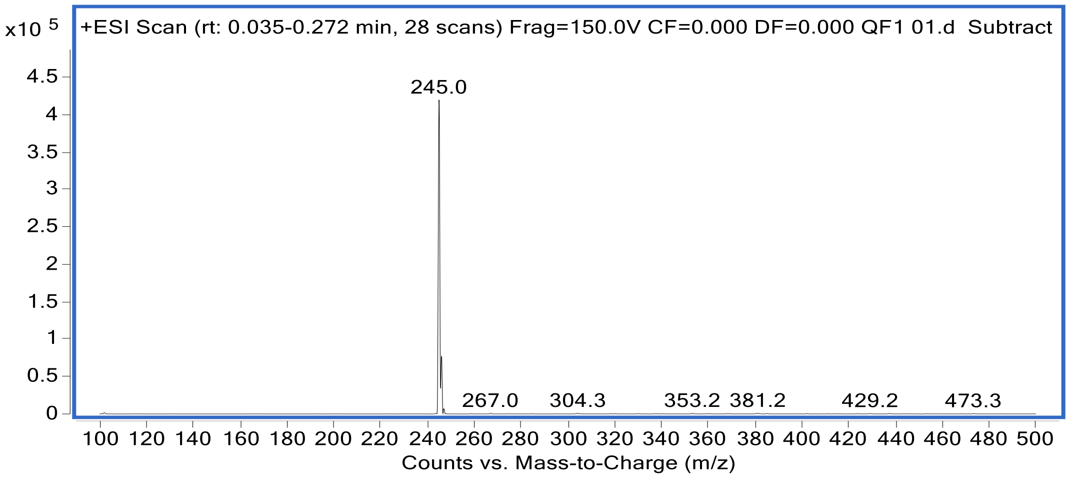

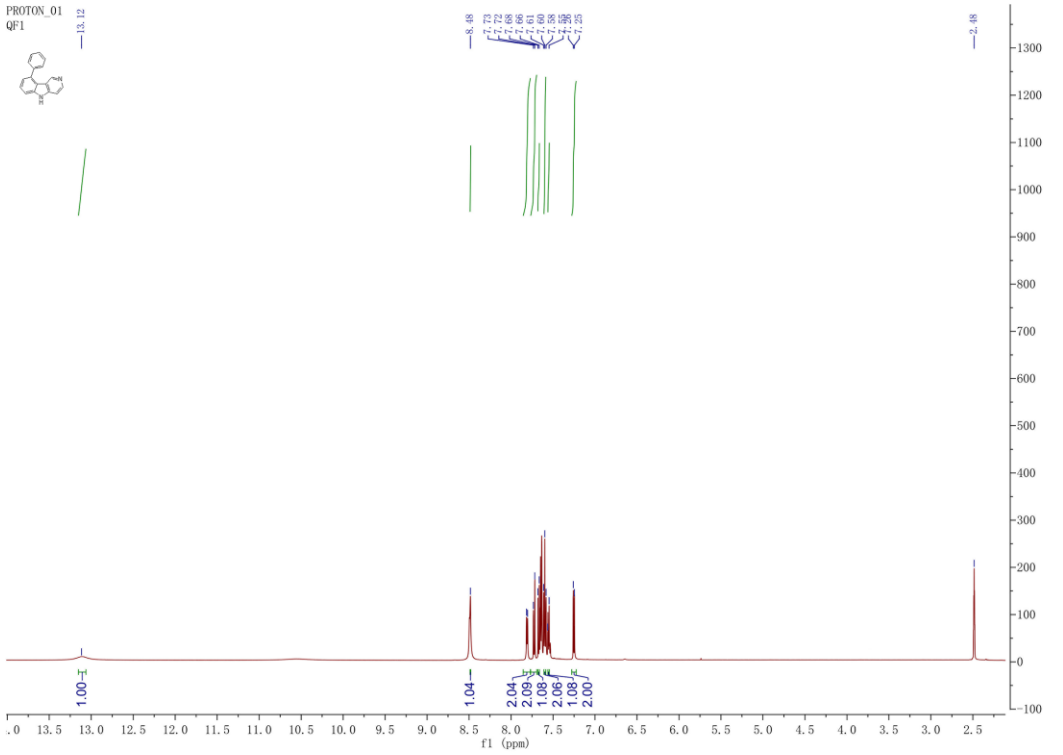

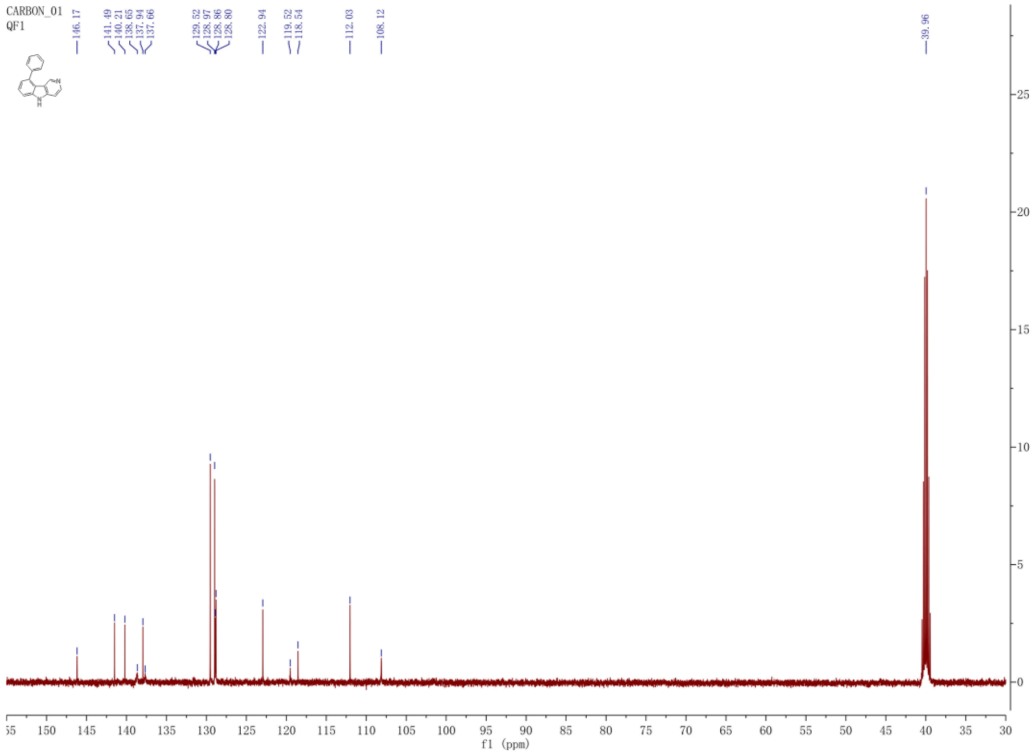

*9-(O-tolyl)-5H-pyrido[4,3-b]indole (**7b**)*

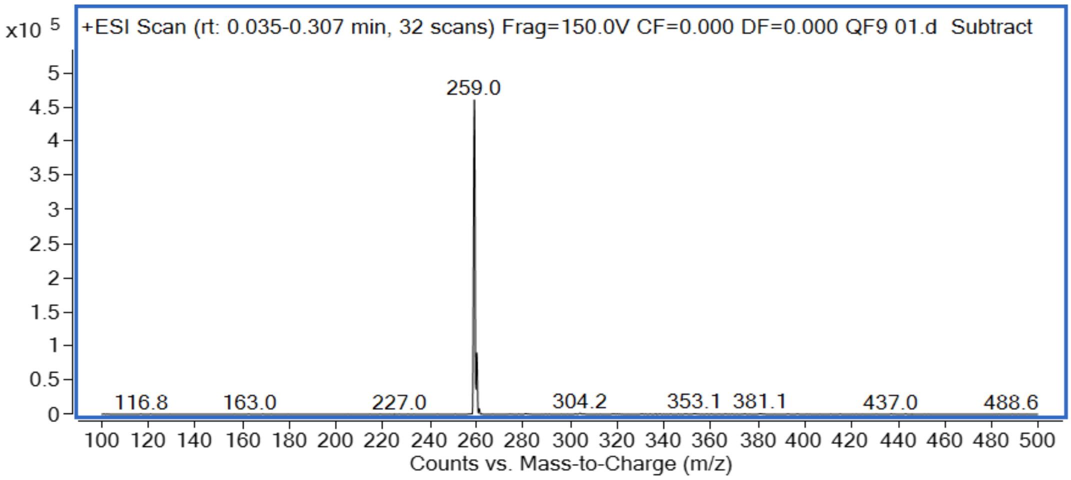

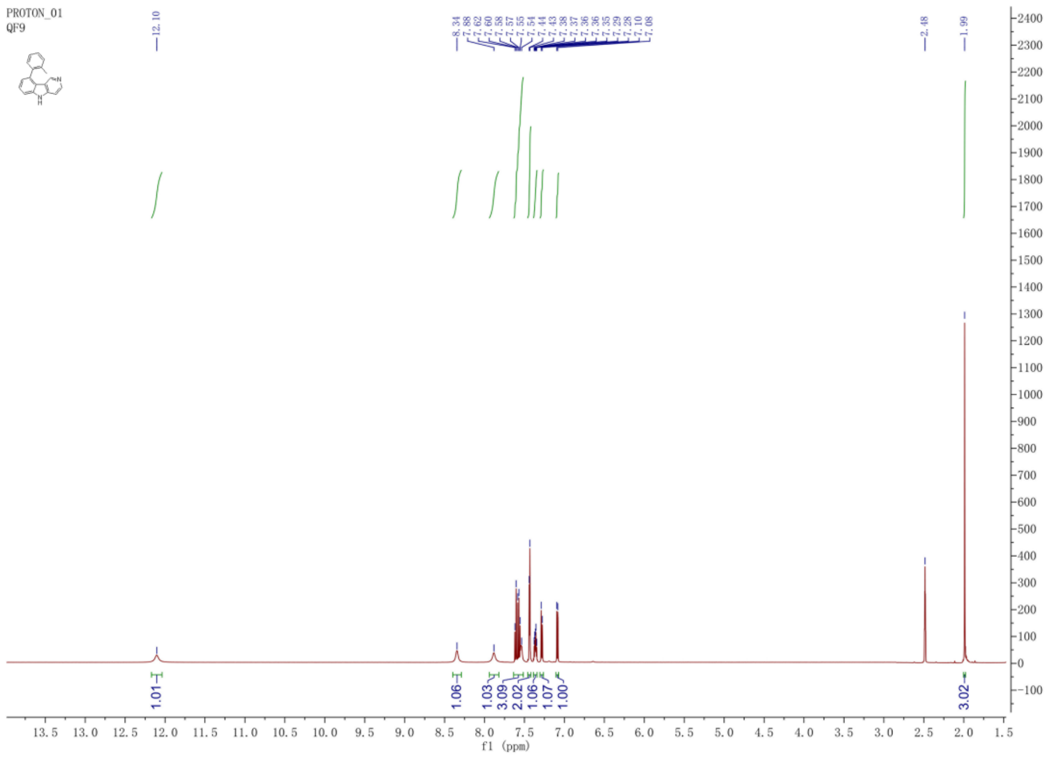
PROTON_01
QF9
12.10
8.34 7.88 7.62 7.60 7.58 7.57 7.55 7.54 7.44 7.43 7.38 7.37 7.36 7.36 7.35 7.29 7.28 7.10 7.08
2.48
1.99
1.01
1.06
1.03 3.09 2.02 1.06 1.07 1.00
3.02
f1 (ppm)

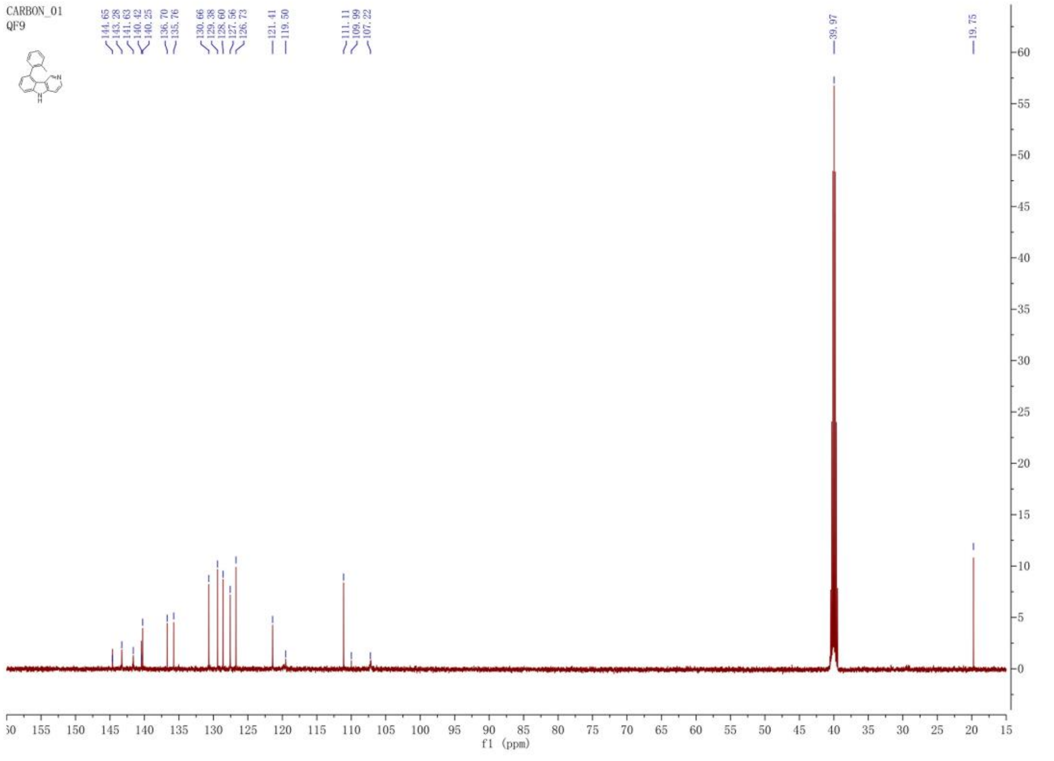
CARBON_01
QF9
144.65 143.28 141.63 140.42 140.25 136.70 135.76 130.66 129.38 128.60 127.56 126.73 121.41 119.50
111.11 109.99 107.22
39.97
19.75
f1 (ppm)

*9-(M-tolyl)-5H-pyrido[4,3-b]indole (**7c**)*

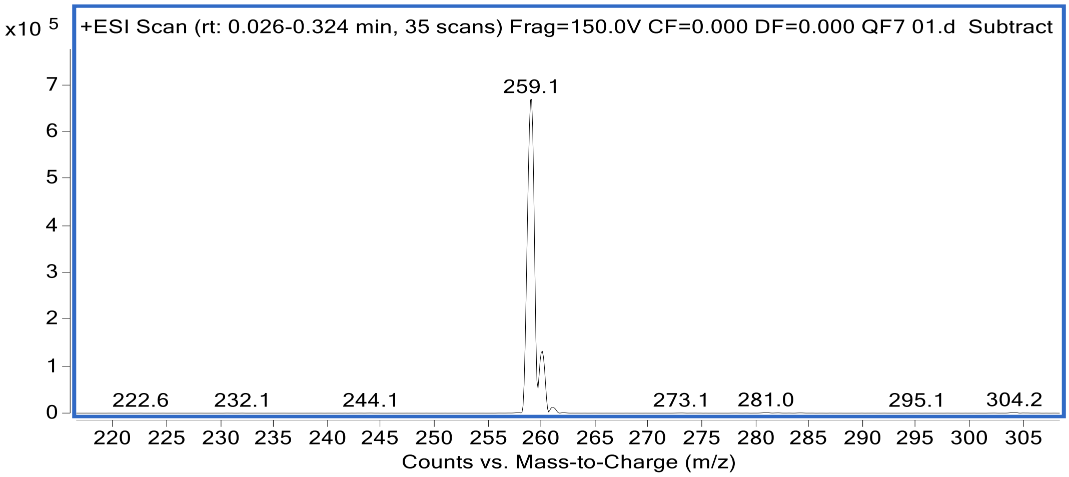

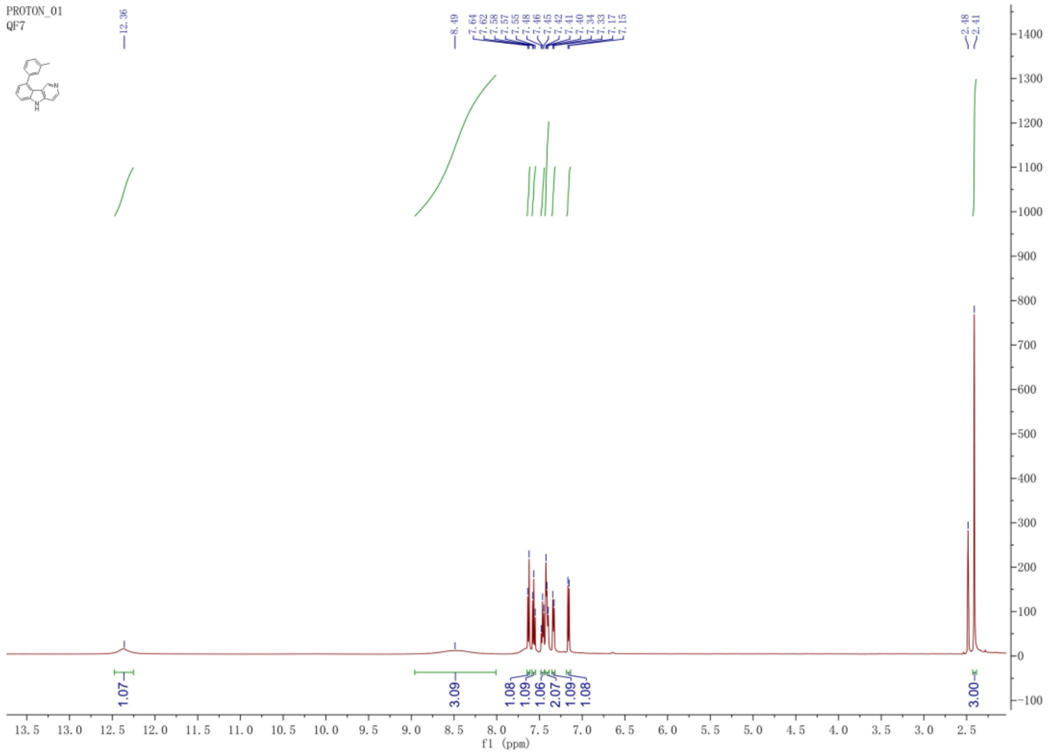

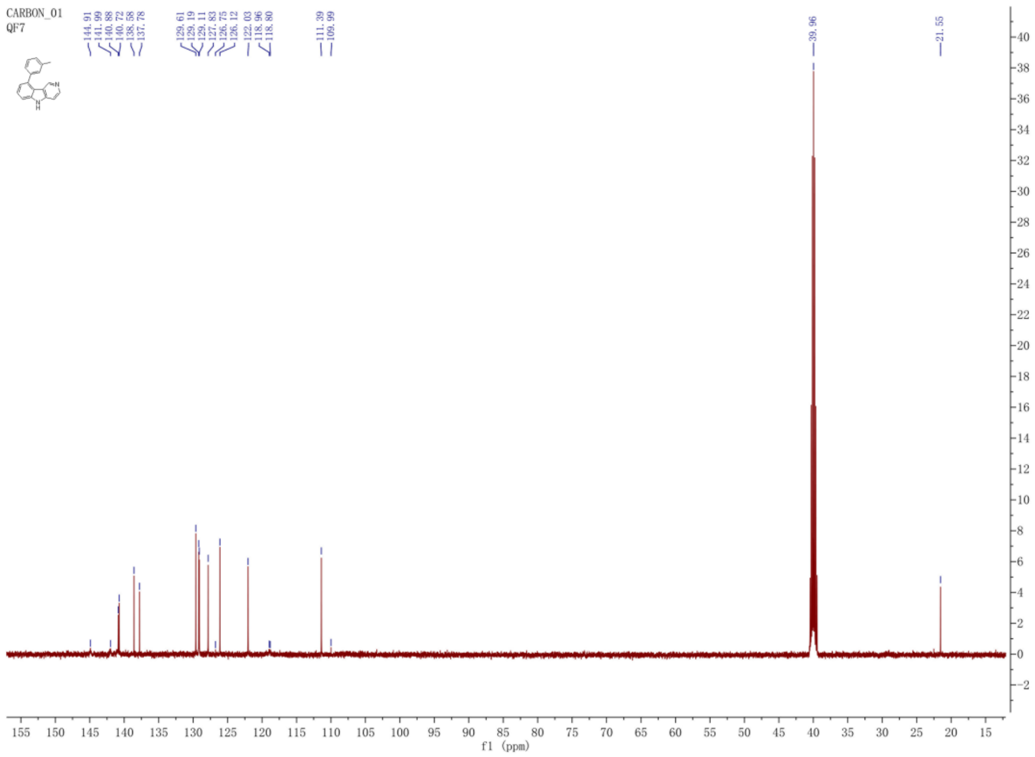

*9-(P-tolyl)-5H-pyrido[4,3-b]indole (**7d**)*

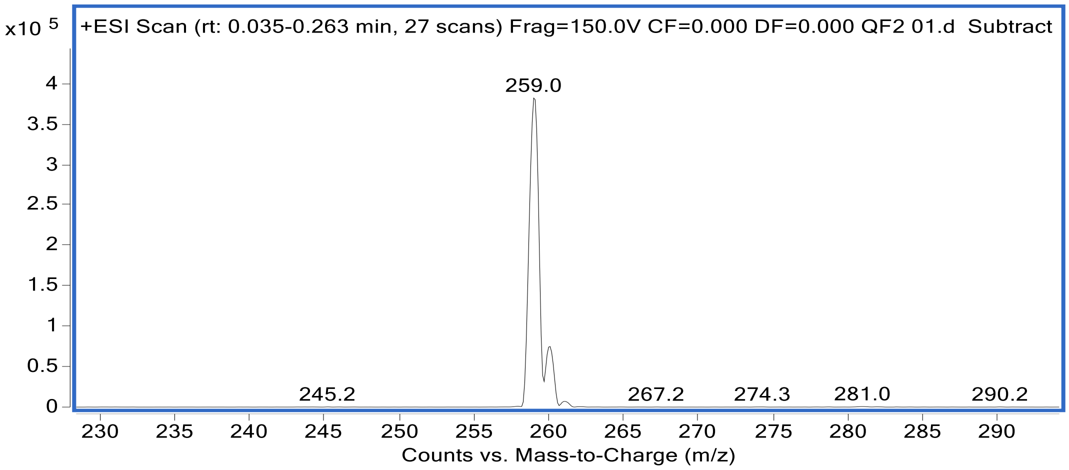

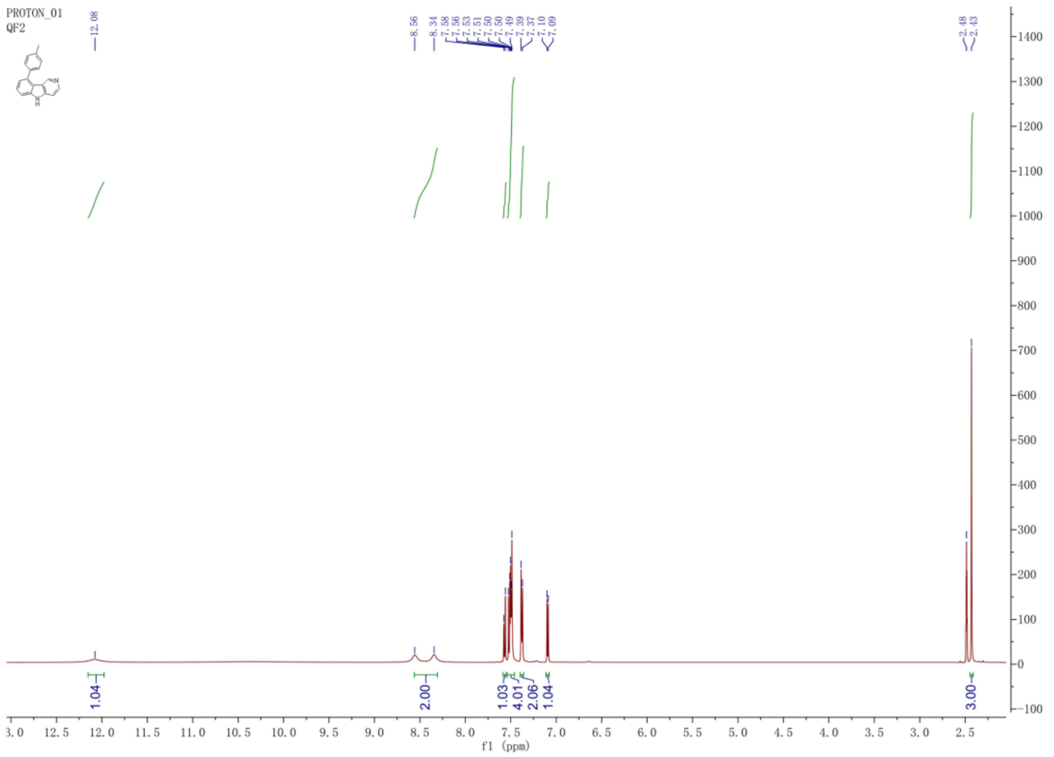
PROTON_01
QF2
12.08
8.56
8.34
7.58
7.56
7.53
7.51
7.50
7.49
7.39
7.37
7.10
7.09
2.48
2.43
1.04
2.00
1.03
4.01
2.06
1.04
3.00
f1 (ppm)

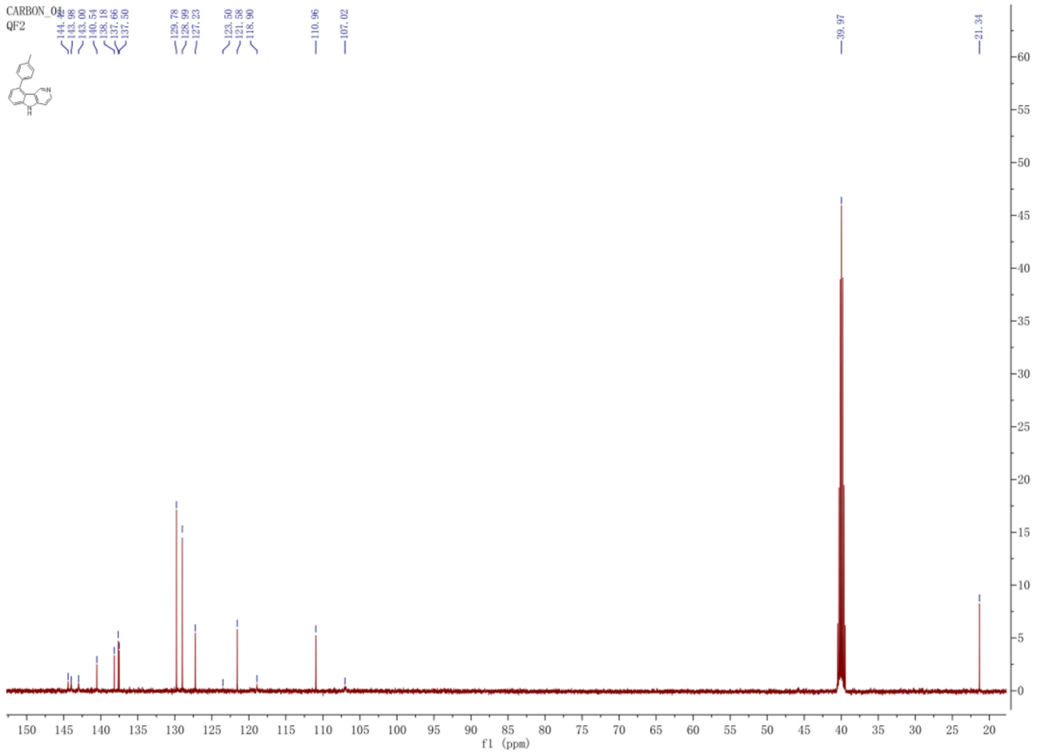
CARBON_01
QF2
144.44
143.98
143.00
140.54
138.18
137.66
137.50
129.78
128.99
127.23
123.50
121.58
118.90
110.96
107.02
39.97
21.34
f1 (ppm)

*9-(3,4-Dimethylphenyl)-5H-pyrido[4,3-b]indole (**7e**)*

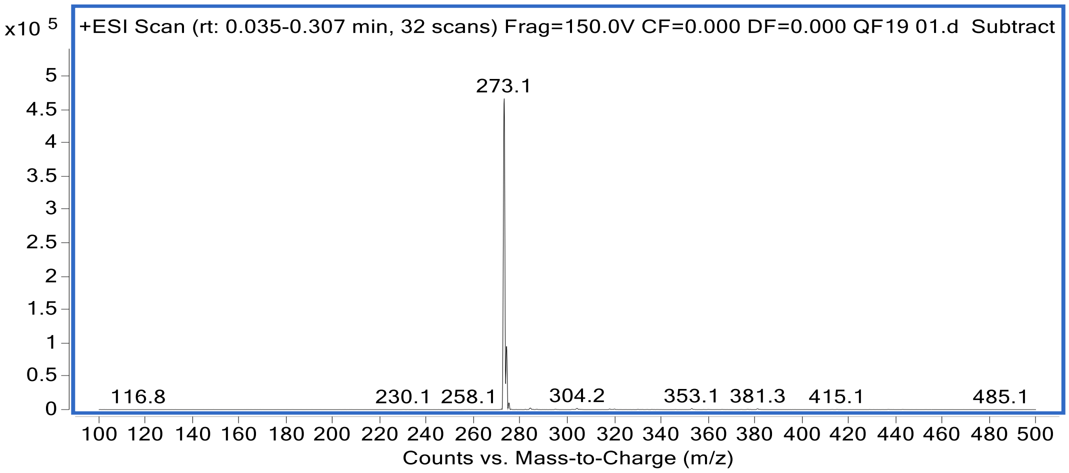

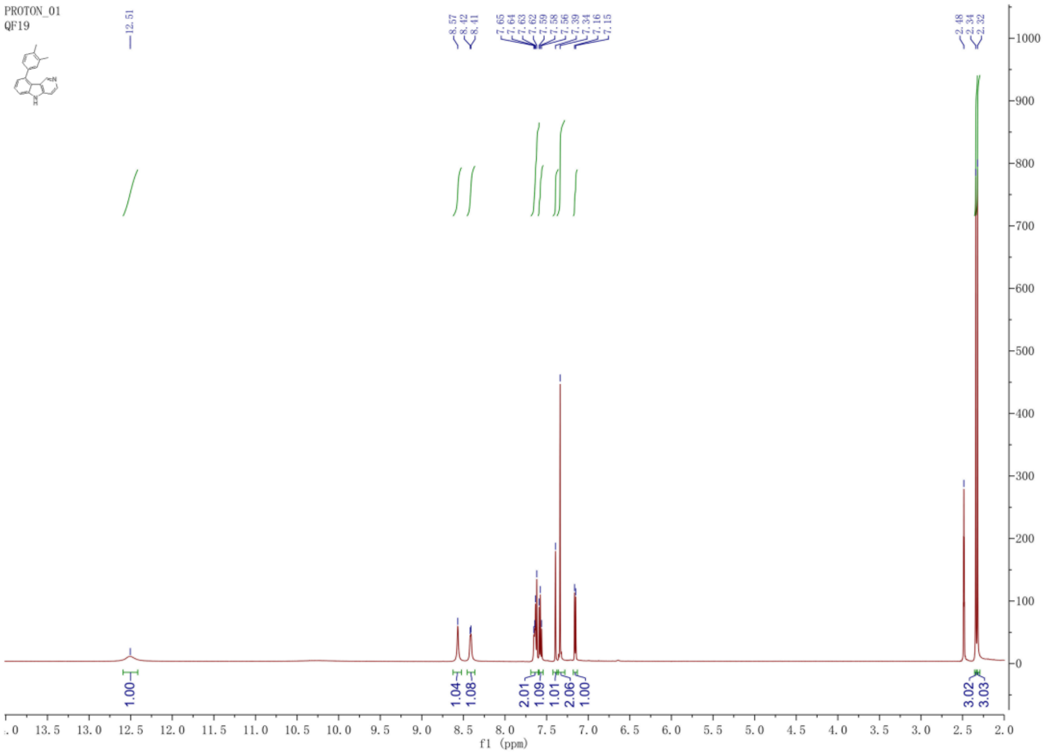

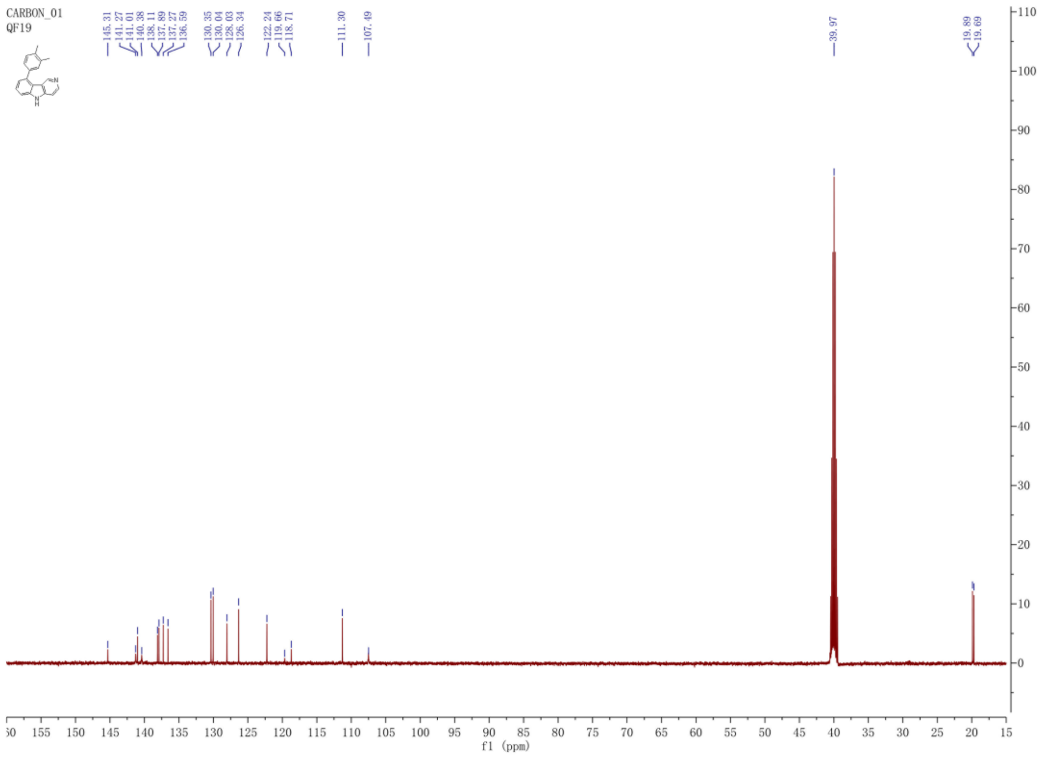

*9-(2-Methoxyphenyl)-5H-pyrido[4,3-b]indole (**7f**)*

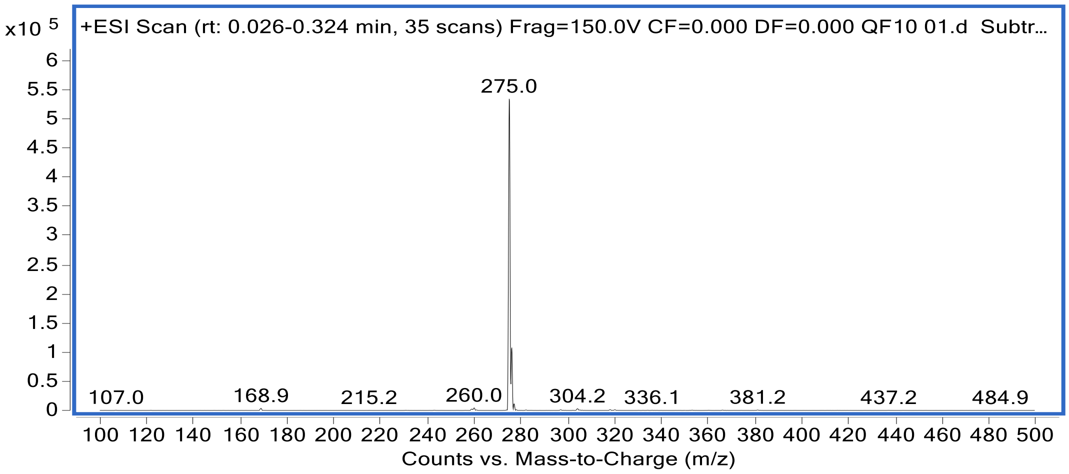

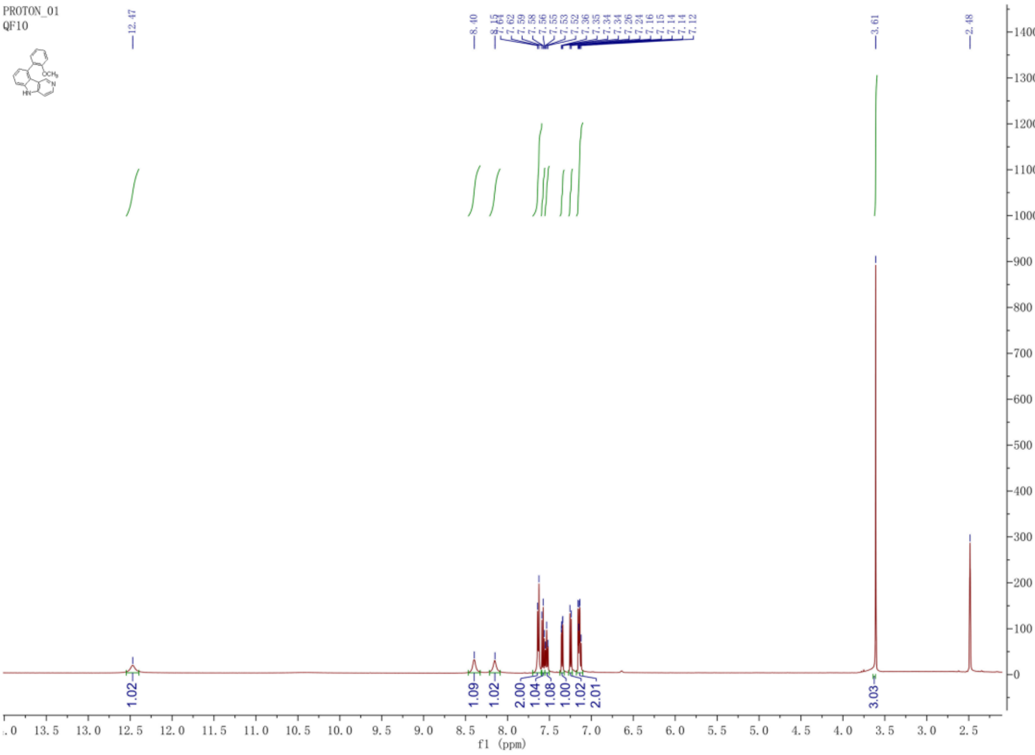
PROTON_01
QF10
OCH3
HN
N
12.47
8.40
8.15
7.64
7.62
7.59
7.58
7.56
7.55
7.53
7.52
7.36
7.35
7.34
7.26
7.24
7.16
7.15
7.14
7.14
7.12
3.61
2.48
1.02
1.09
1.02
2.00
1.04
1.08
1.00
1.02
2.01
3.03
f1 (ppm)

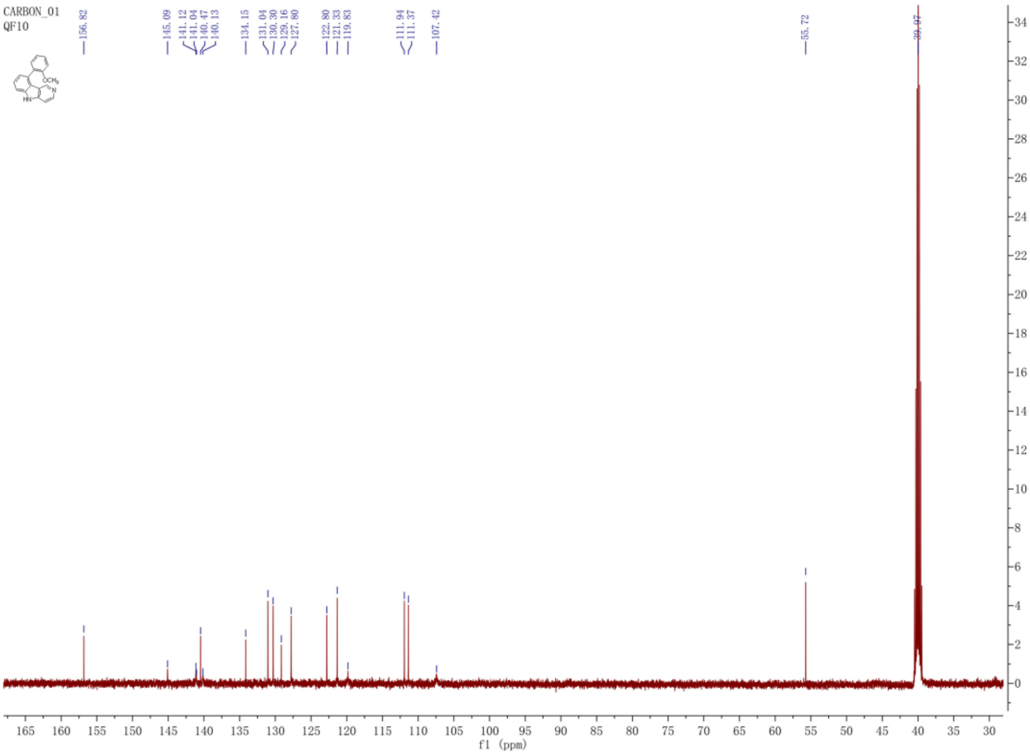
CARBON_01
QF10
OCH3
HN
N
156.82
145.09
141.12
141.04
140.47
140.13
134.15
131.04
130.30
129.16
127.80
122.80
121.33
119.83
111.94
111.37
107.42
55.72
39.97
f1 (ppm)

*9-(3-Methoxyphenyl)-5H-pyrido[4,3-b]indole (**7g**)*

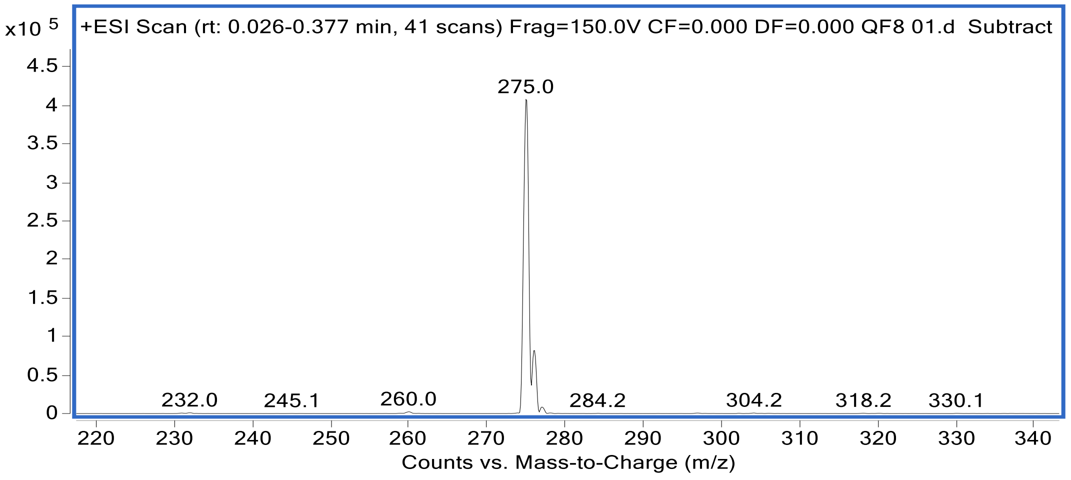

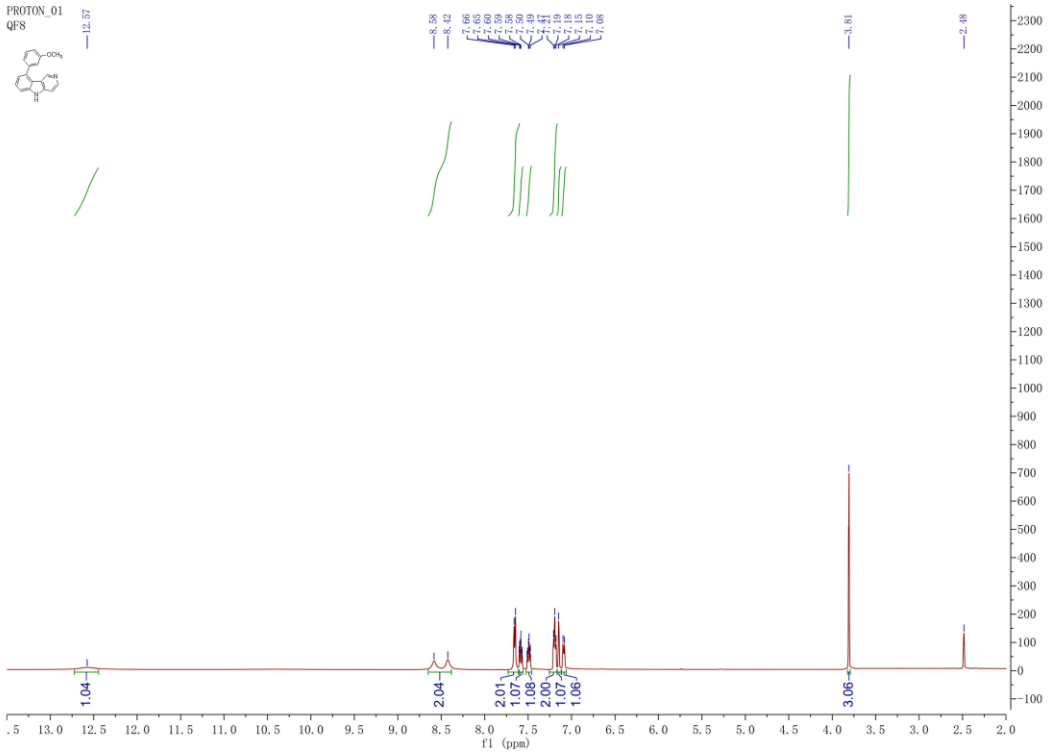

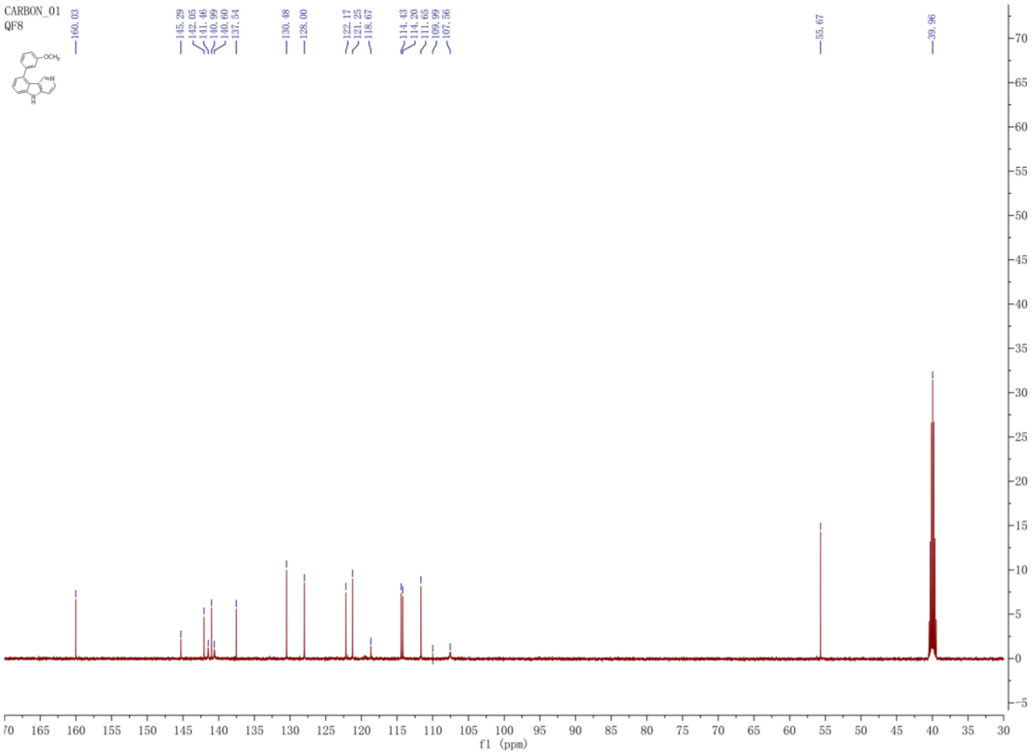

9-(4-Methoxyphenyl)-5H-pyrido[4,3-b]indole (**7h**)

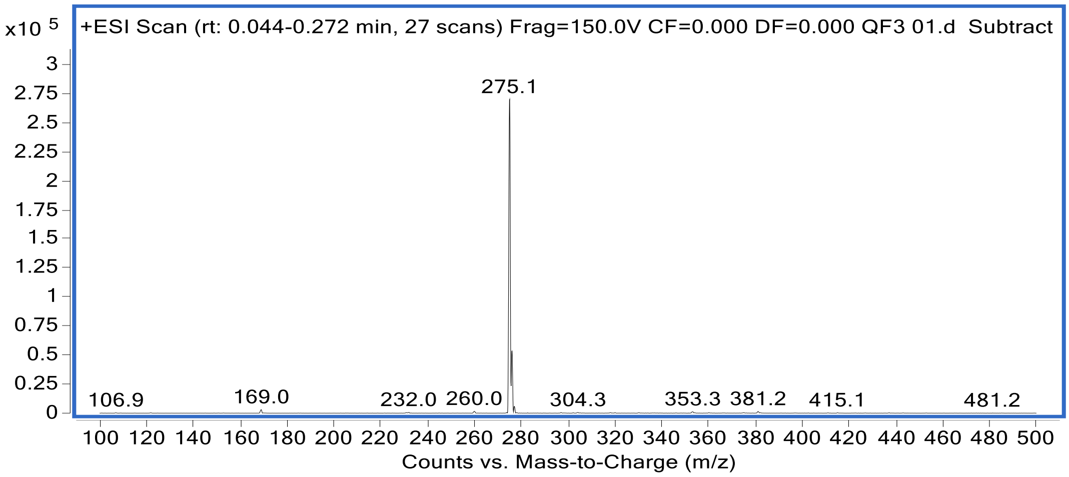

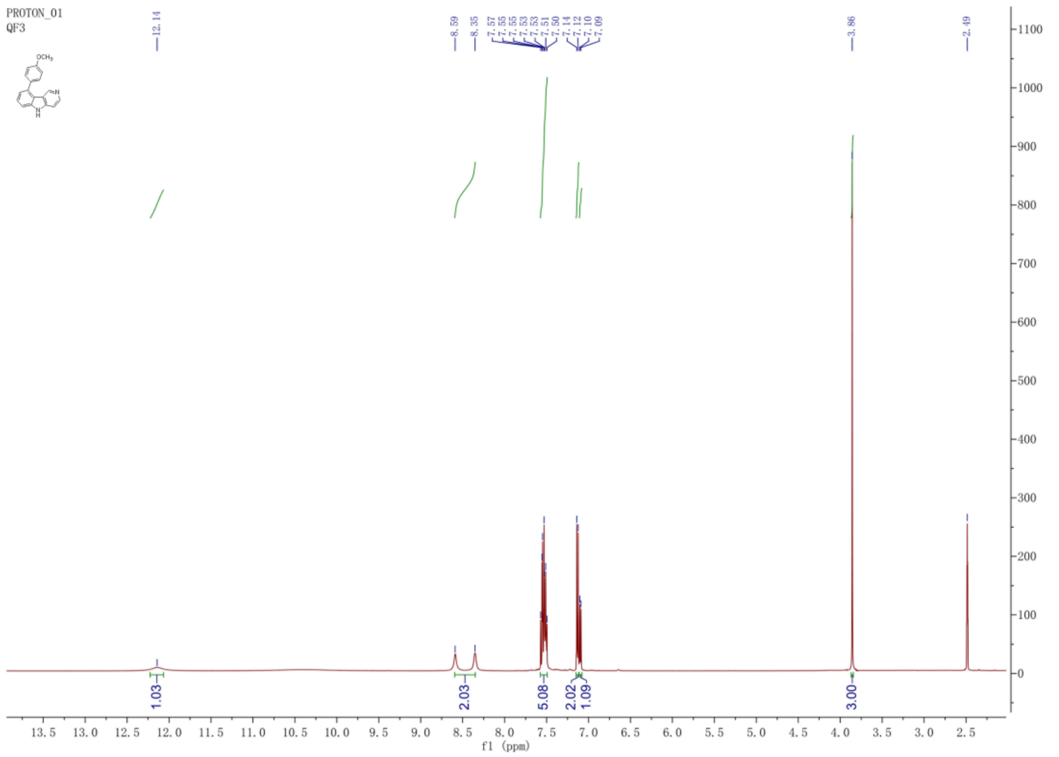

PROTON_01
QF3
OCH3
N
H
12.14
8.59
8.35
7.57
7.55
7.55
7.53
7.53
7.51
7.50
7.14
7.12
7.10
7.09
3.86
2.49
1.03
2.03
5.08
2.02
1.09
3.00
f1 (ppm)

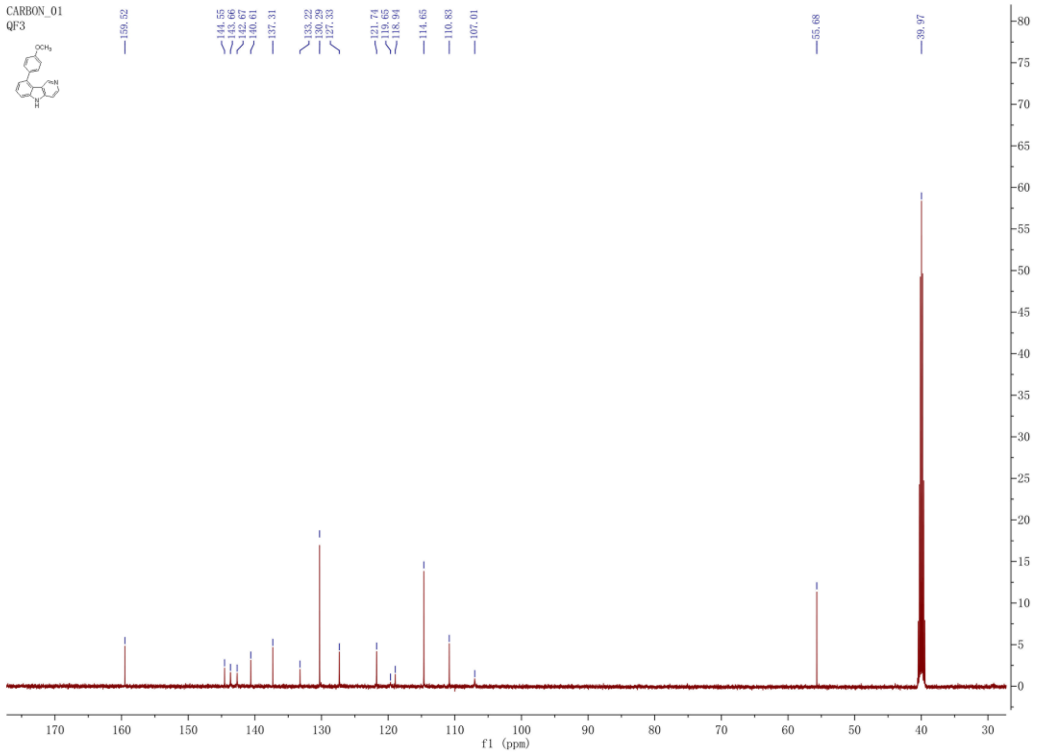

CARBON_01
QF3
OCH3
N
H
159.52
144.55
143.66
142.67
140.61
137.31
133.22
130.29
127.33
121.74
119.65
118.94
114.65
110.83
107.01
55.68
39.97
f1 (ppm)

*2-Methoxy-5-(5H-pyrido[4,3-b]indol-9-yl)phenol (**7i**)*

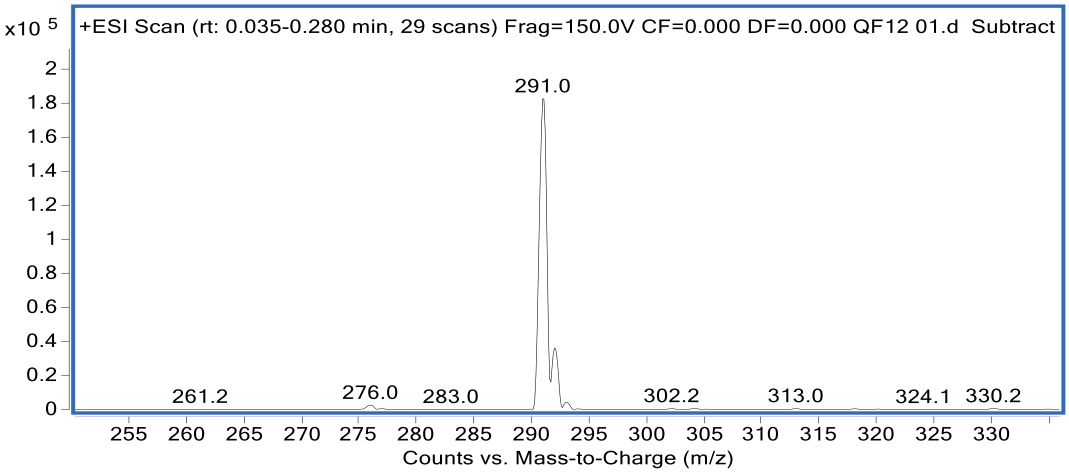

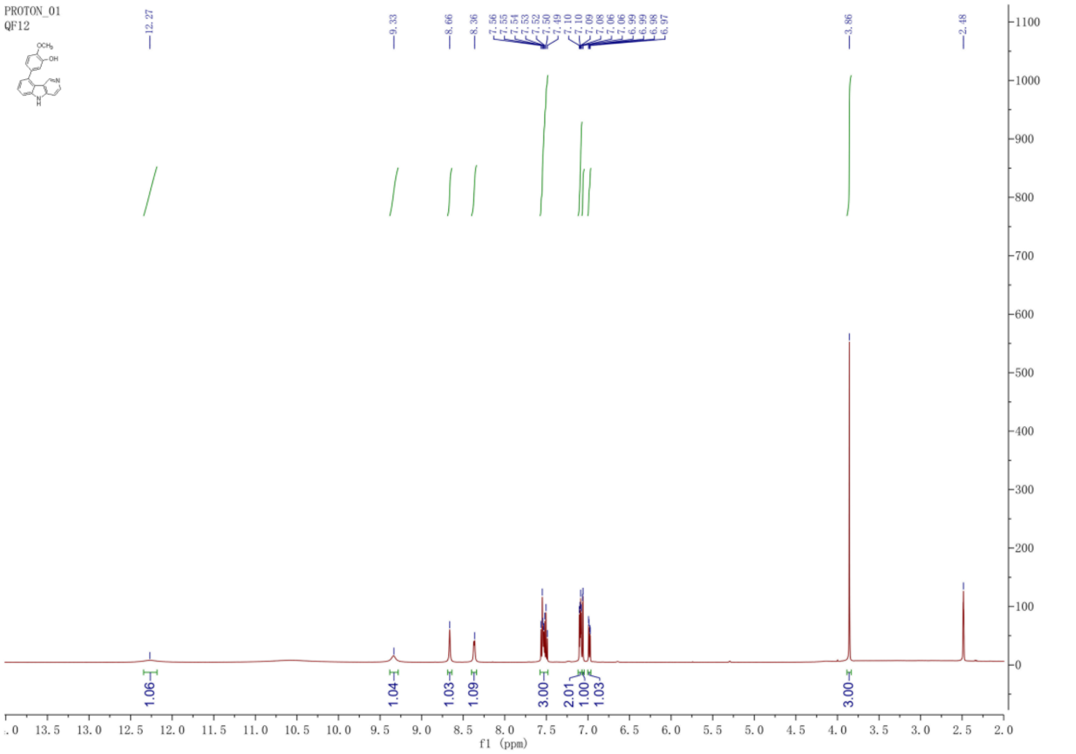

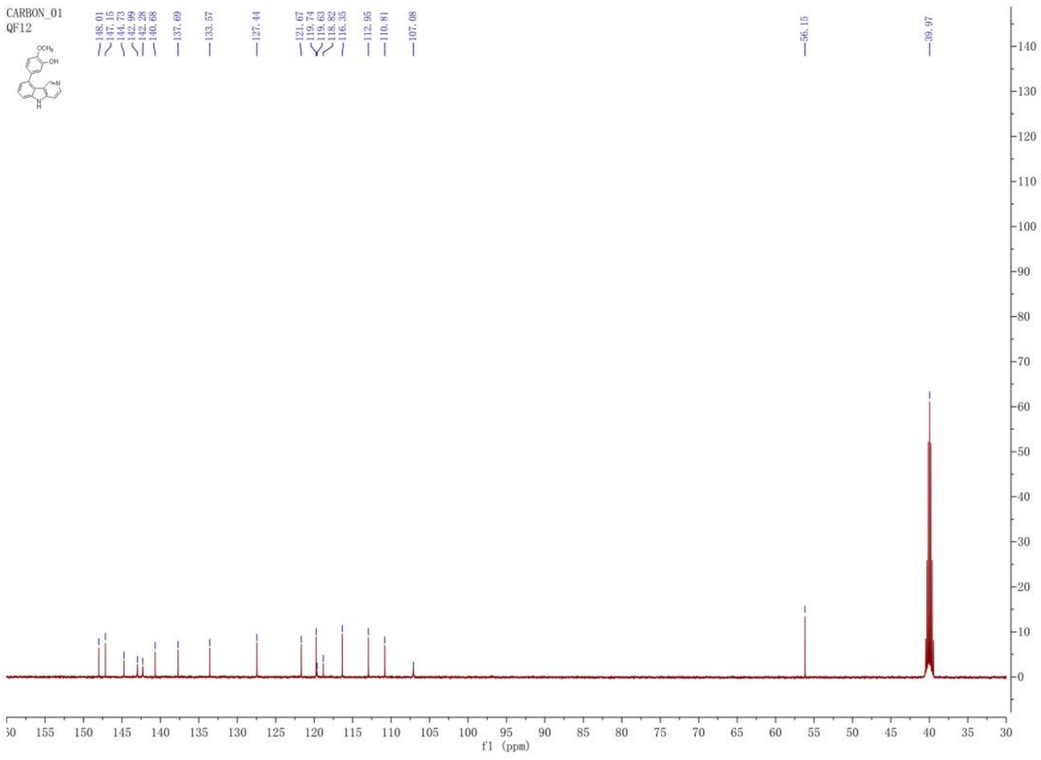

9-(3,4-Dimethoxyphenyl)-5H-pyrido[4,3-b]indole (**7j**)

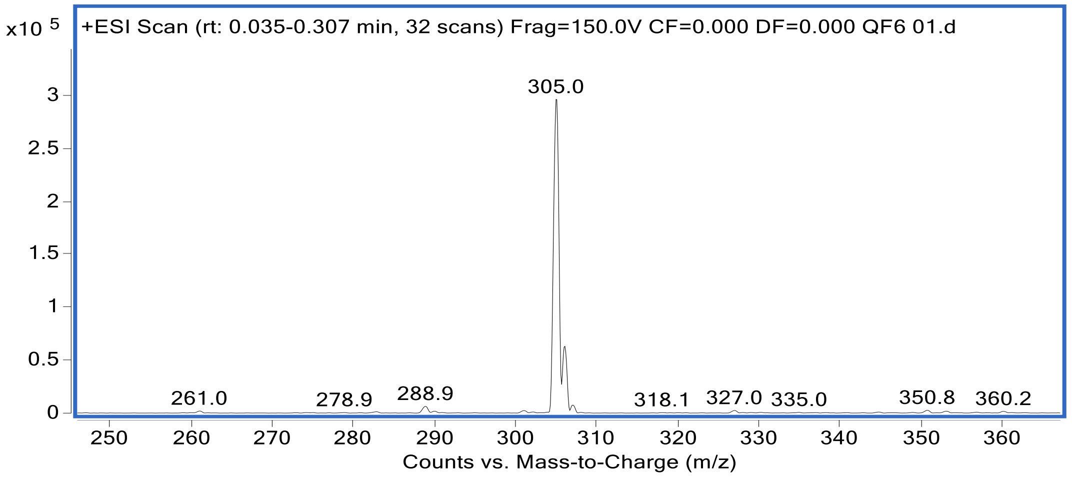

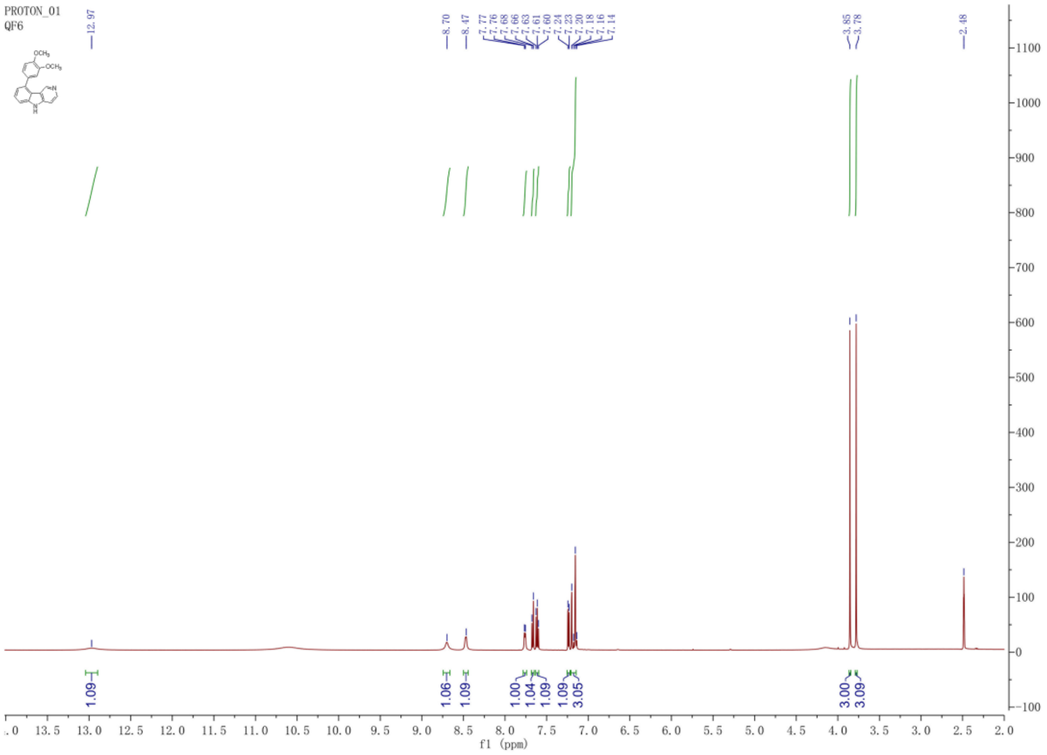
PROTON_01
QF6
12.97
8.70
8.47
7.77
7.76
7.68
7.66
7.63
7.61
7.60
7.24
7.23
7.20
7.18
7.16
7.14
3.85
3.78
2.48
1.09
1.06
1.09
1.00
1.04
1.09
1.09
3.05
3.00
3.09
f1 (ppm)

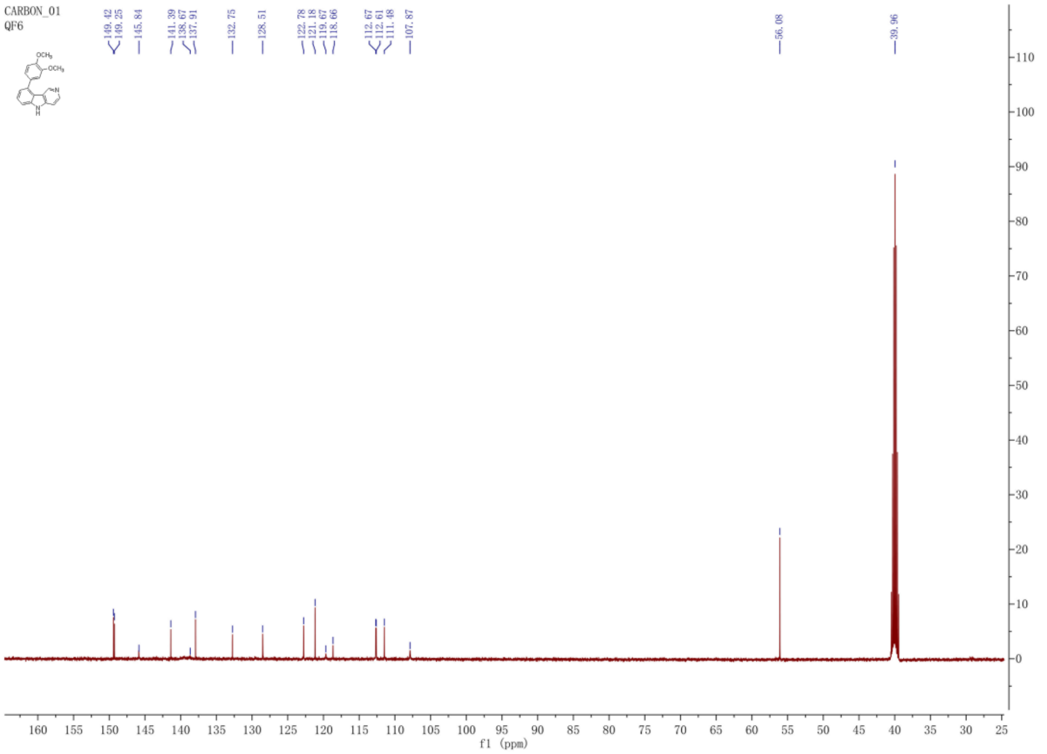
CARBON_01
QF6
149.42
149.25
145.84
141.39
138.67
137.91
132.75
128.51
122.78
121.18
119.67
118.66
112.67
112.61
111.48
107.87
56.08
39.96
f1 (ppm)

*9-(3,4,5-Trimethoxyphenyl)-5H-pyrido[4,3-b]indole (**7k**)*

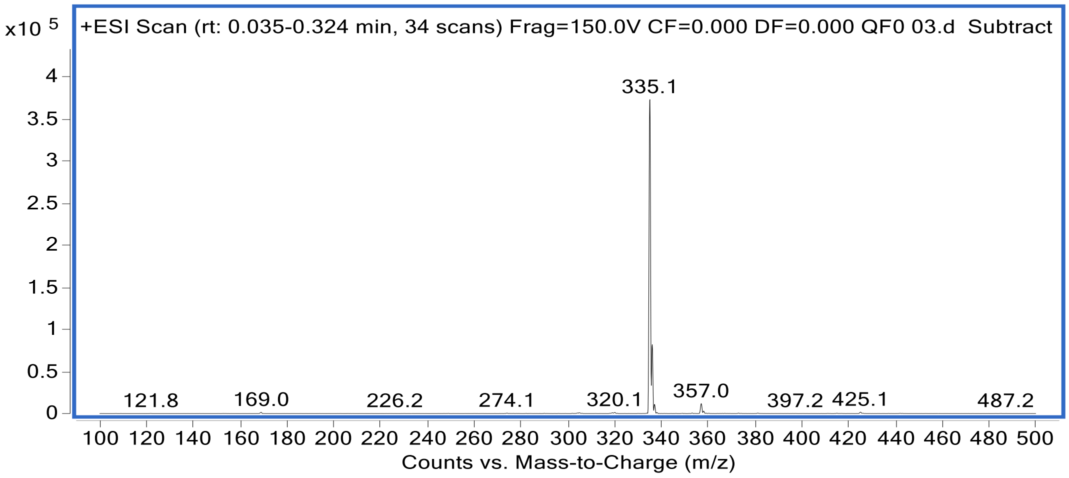

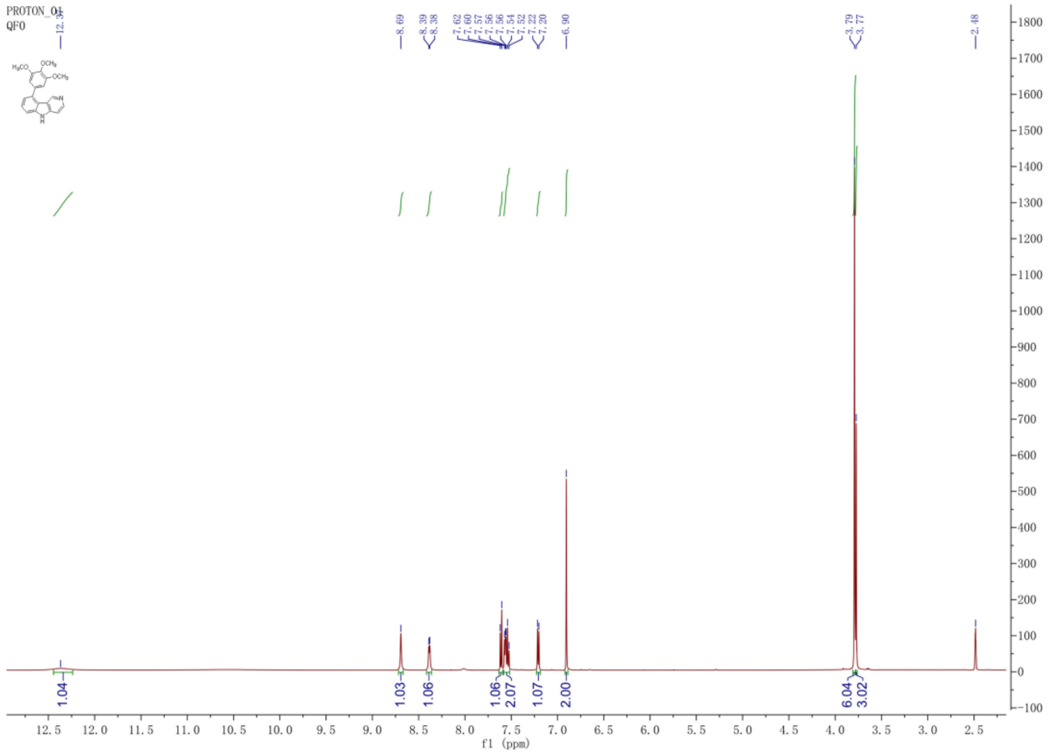

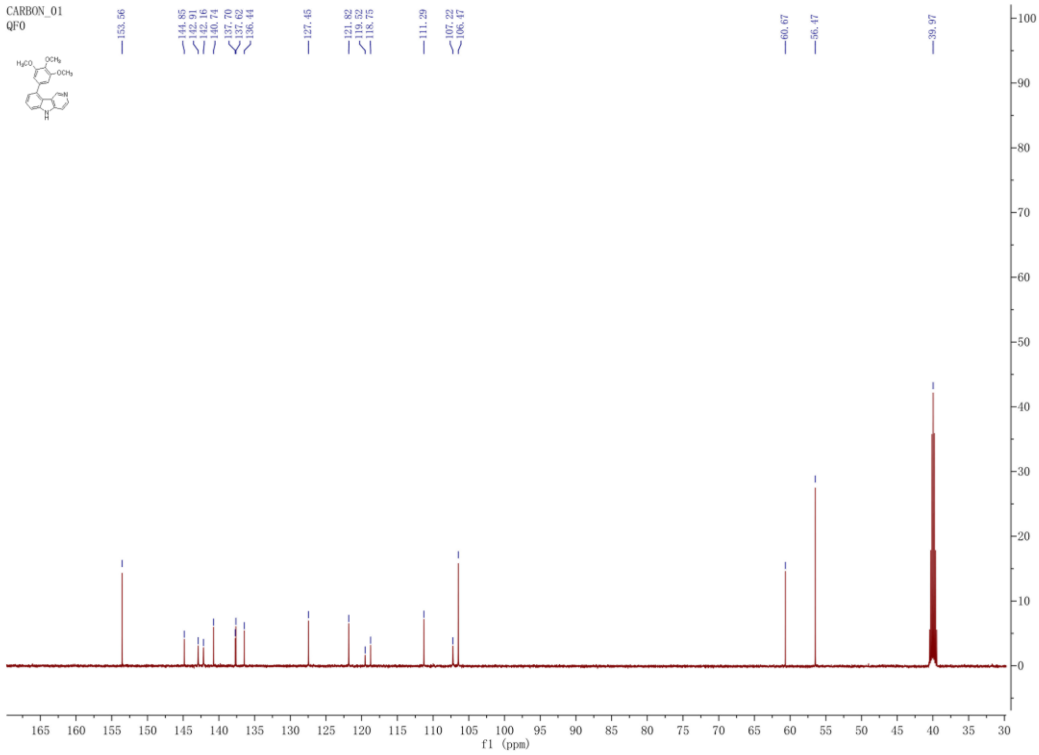

*9-(4-Ethoxyphenyl)-5H-pyrido[4,3-b]indole (**7l**)*

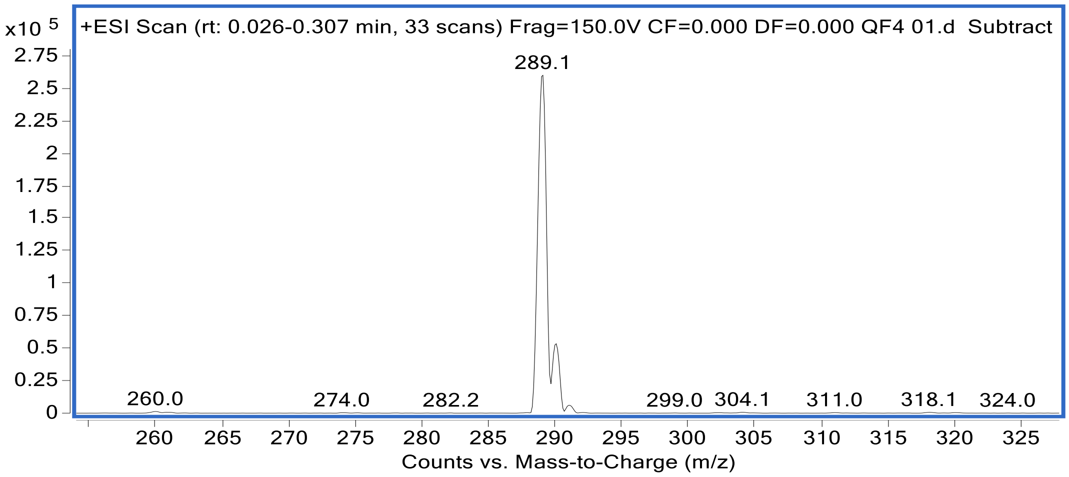

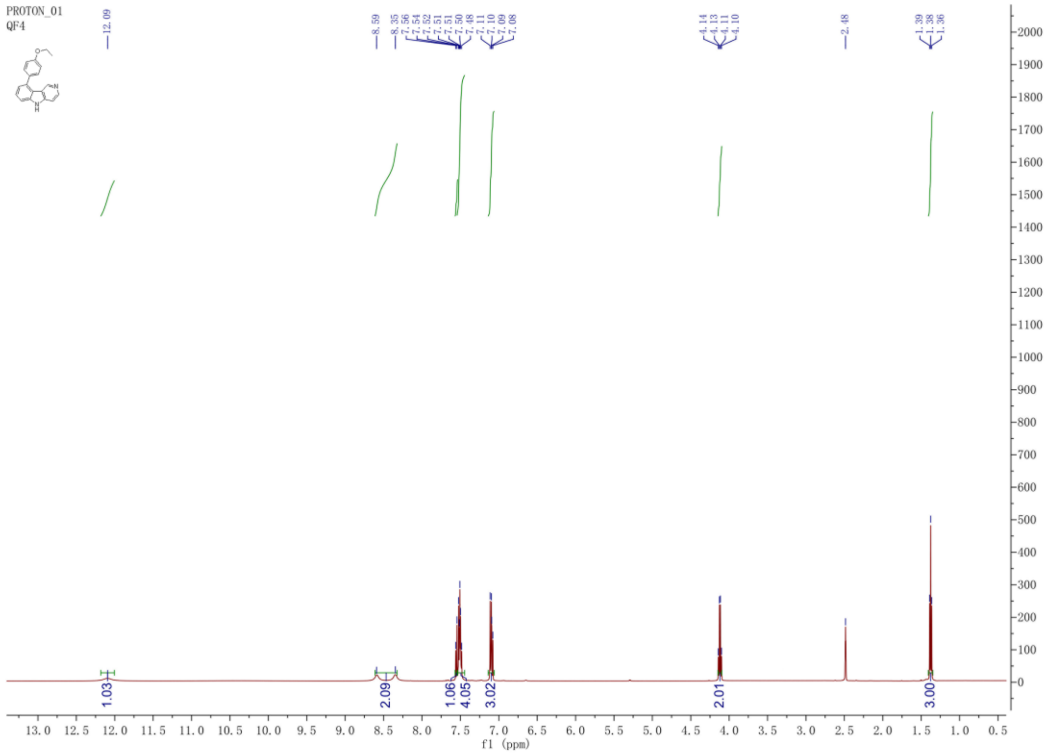
PROTON_01
QF4
12.09
8.59 8.35 7.56 7.54 7.52 7.51 7.51 7.50 7.48 7.11 7.10 7.09 7.08
4.14 4.13 4.11 4.10
2.48
1.39 1.38 1.36
1.03 2.09 1.06 4.05 3.02 2.01 3.00
f1 (ppm)

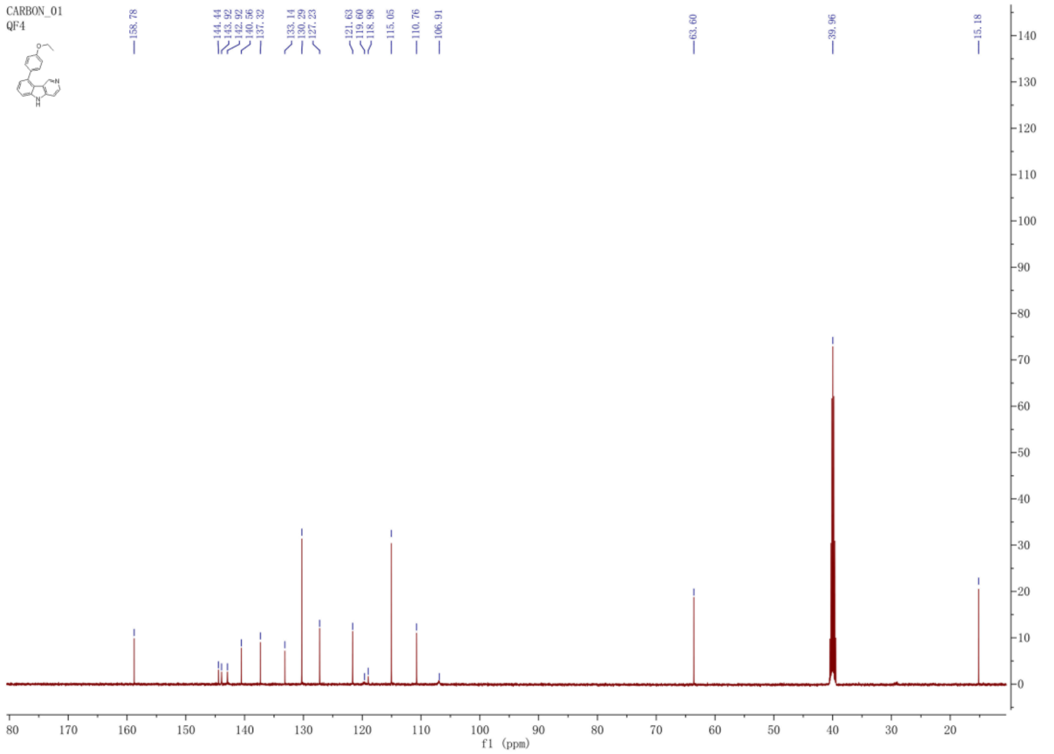
CARBON_01
QF4
158.78
144.44 143.92 142.92 140.56 137.32 133.14 130.29 127.23 121.63 119.60 118.98 115.05 110.76 106.91
63.60
39.96
15.18
f1 (ppm)

*9-(4-Fluorophenyl)-5H-pyrido[4,3-b]indole (**7m**)*

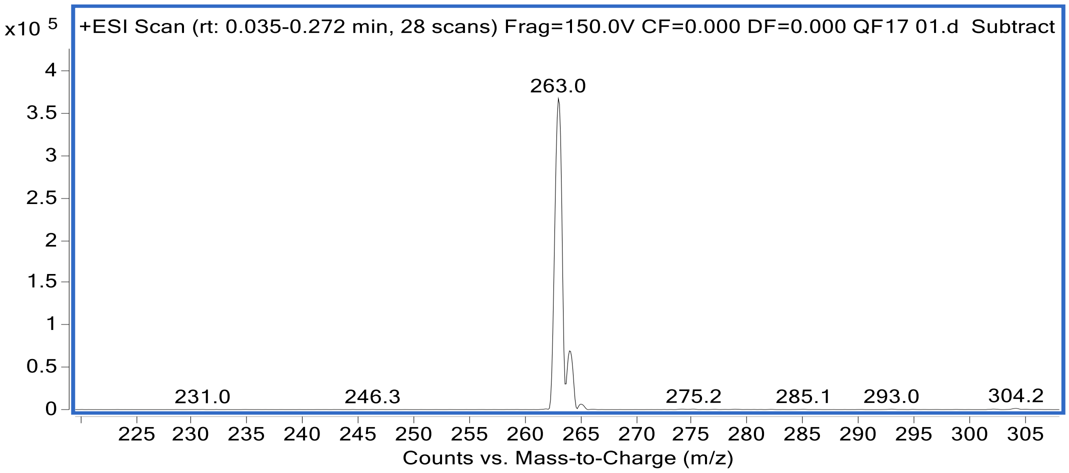

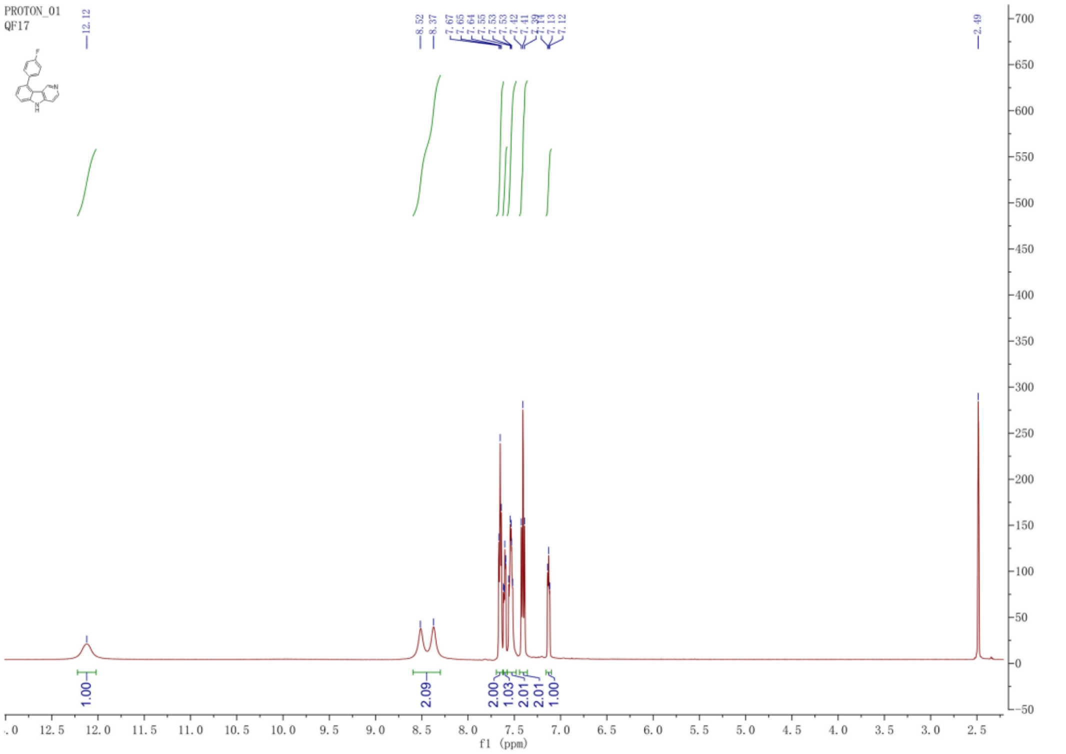

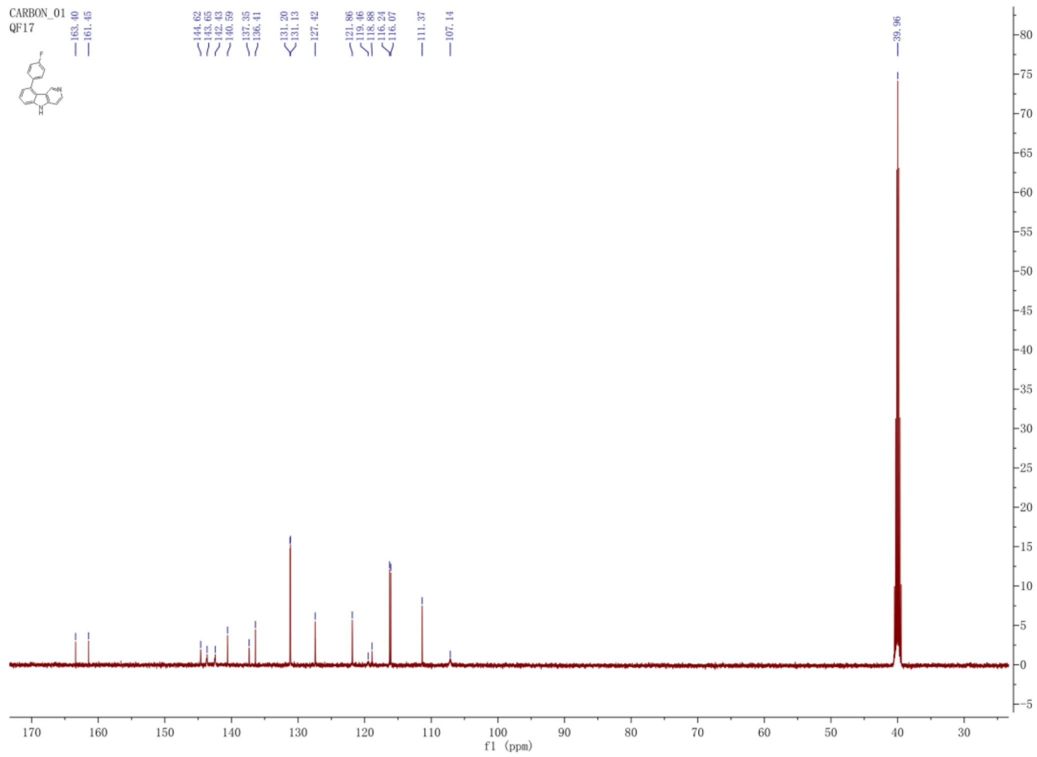

*9-(4-Chlorophenyl)-5H-pyrido[4,3-b]indole (**7n**)*

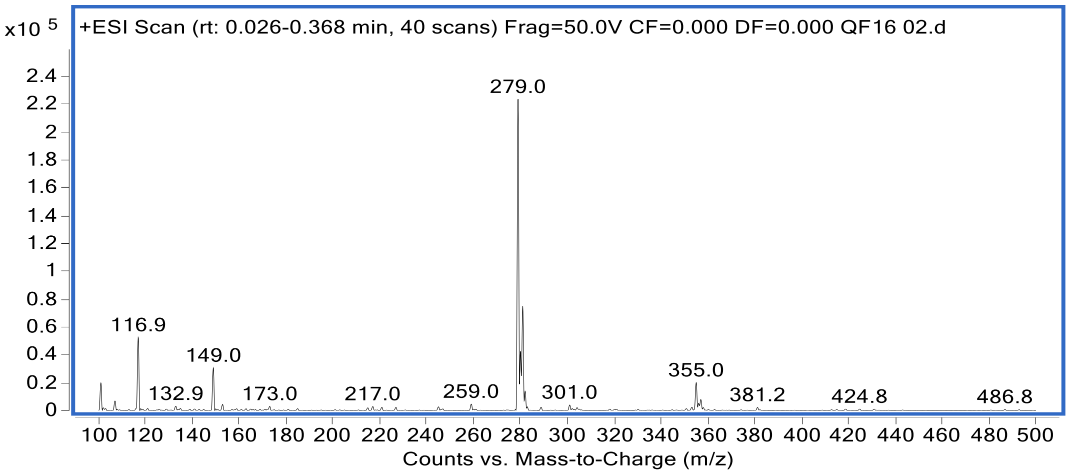

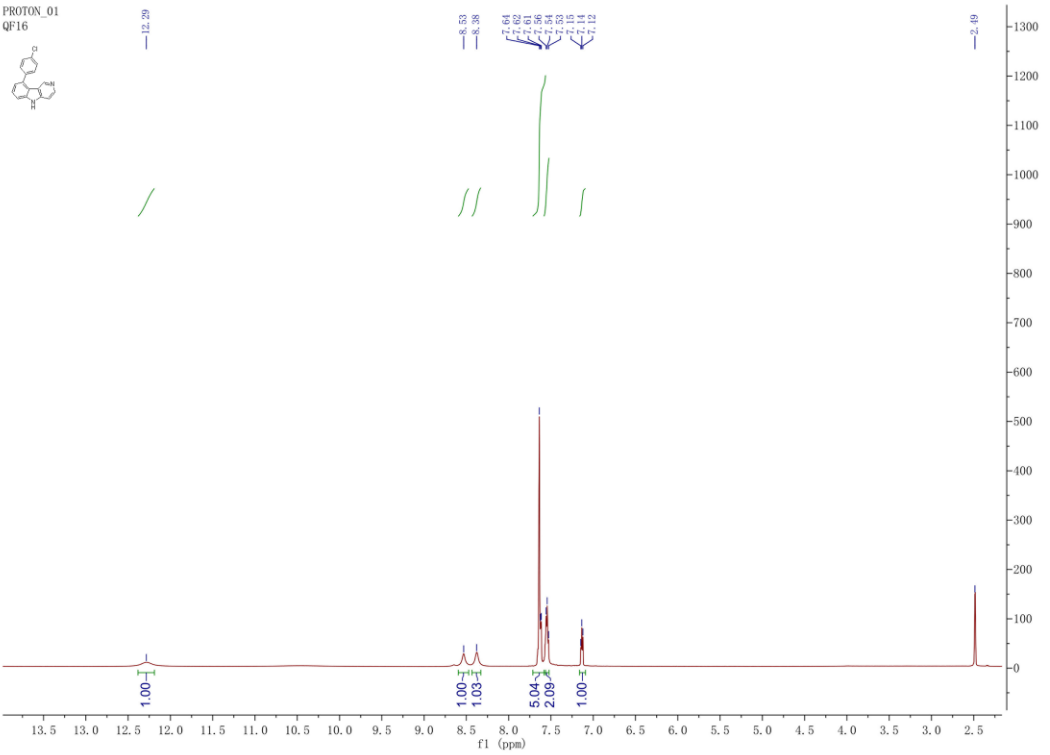
PROTON_01
QF16
12.29
8.53
8.38
7.64
7.62
7.61
7.56
7.54
7.53
7.15
7.14
7.12
2.49
1.00
1.00
1.03
5.04
2.09
1.00
f1 (ppm)

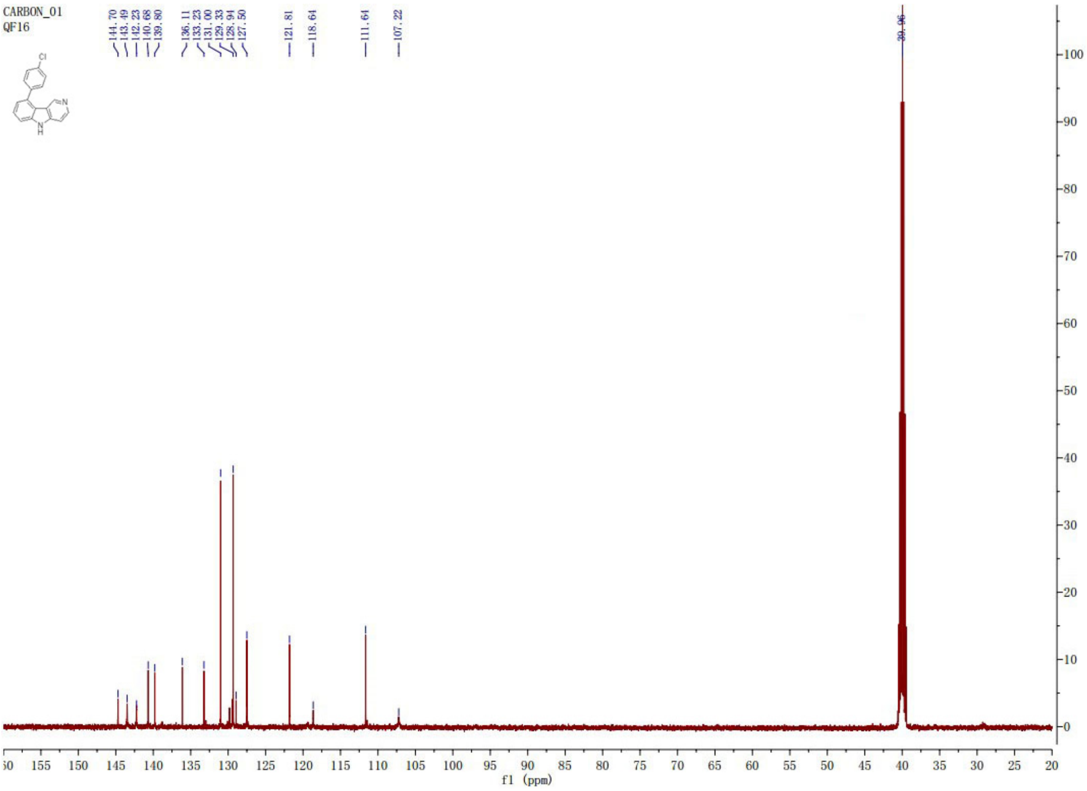
CARBON_01
QF16
144.70
143.49
142.23
140.68
139.80
136.11
133.23
131.00
129.33
128.94
127.50
121.81
118.64
111.64
107.22
39.96
f1 (ppm)

*9-(4-Nitrophenyl)-5H-pyrido[4,3-b]indole (**7o**)*

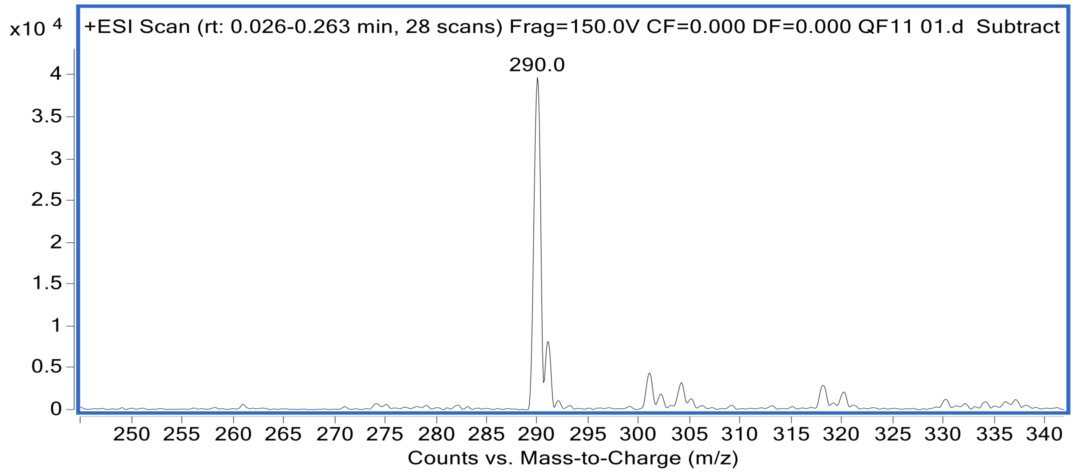

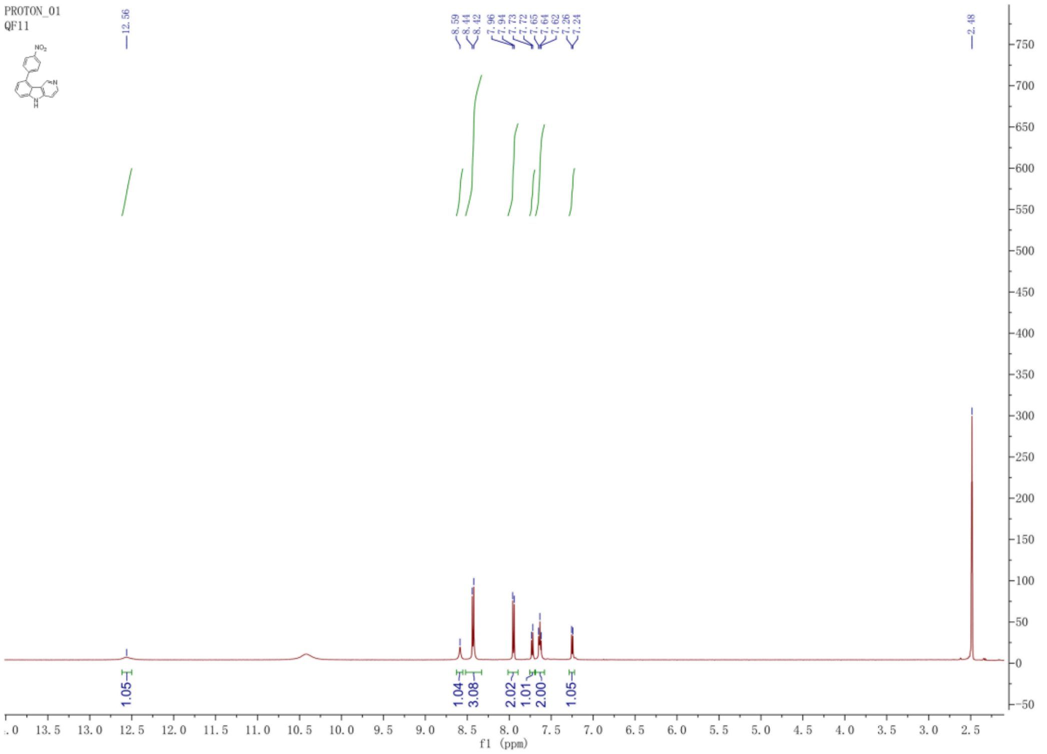

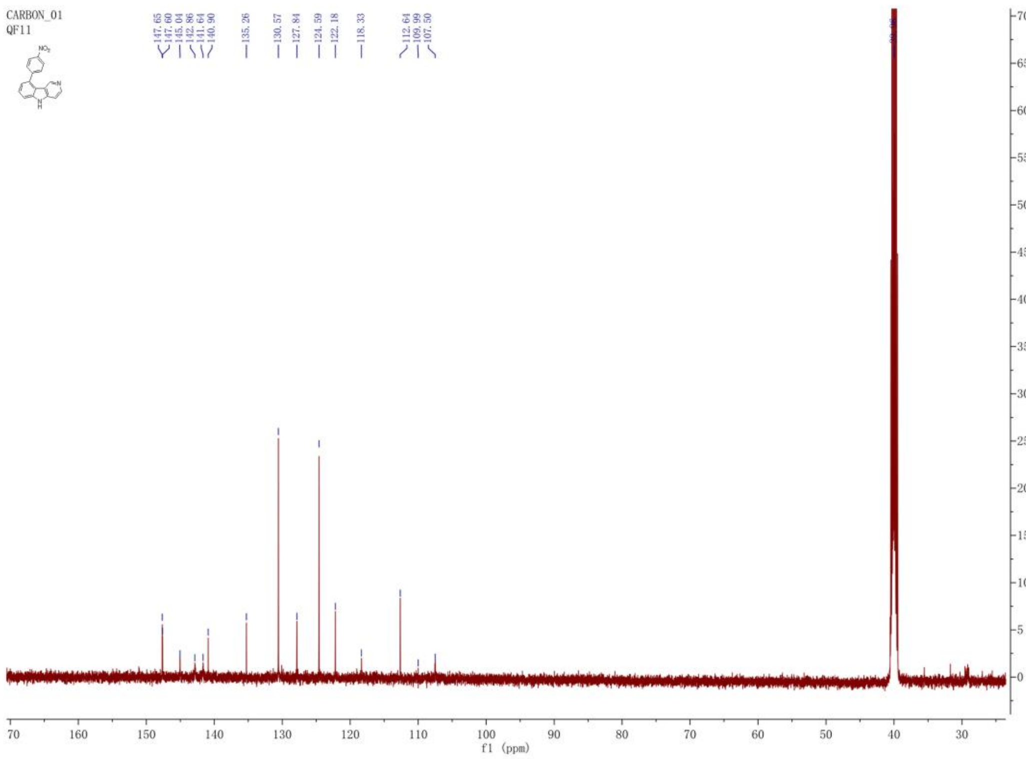

*4-(5H-pyrido[4,3-b]indol-9-yl)benzaldehyde (**7p**)*

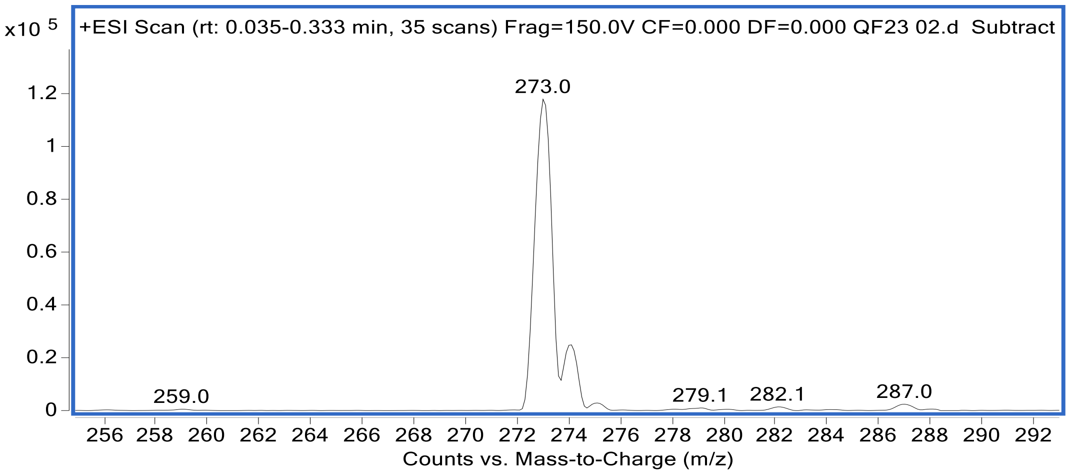

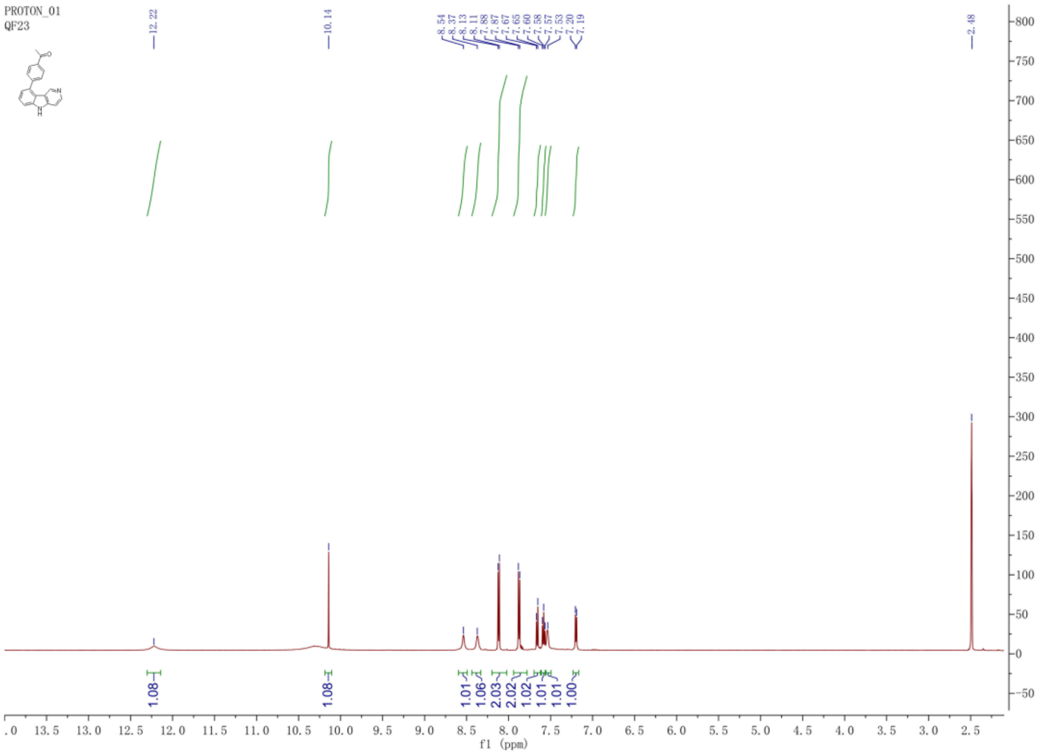
PROTON_01
QF23
12.22
10.14
8.54 8.37 8.13 8.11 7.88 7.87 7.67 7.65 7.60 7.58 7.57 7.53 7.20 7.19
2.48
1.08 1.08 1.01 1.06 2.03 2.02 1.02 1.01 1.01 1.00
f1 (ppm)

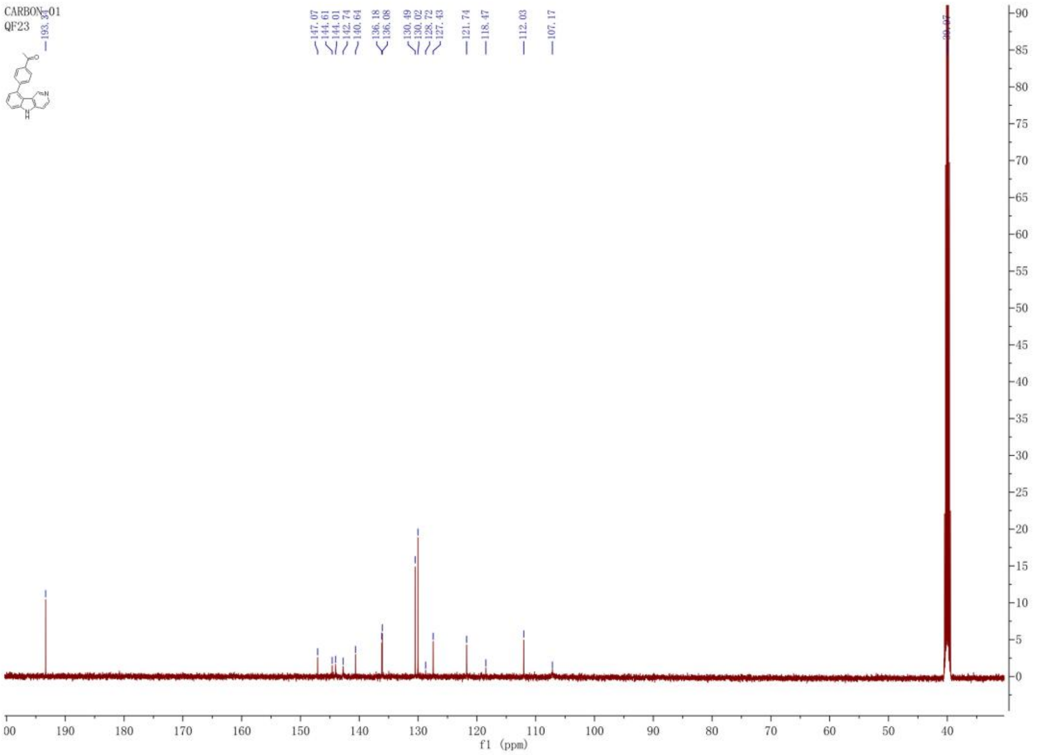
CARBON_01
QF23
193.37
147.07 144.61 144.01 142.74 140.64 136.18 136.08 130.49 130.02 128.72 127.43 121.74 118.47 112.03 107.17
f1 (ppm)

*9-(Naphthalen-2-yl)-5H-pyrido[4,3-b]indole (**7q**)*

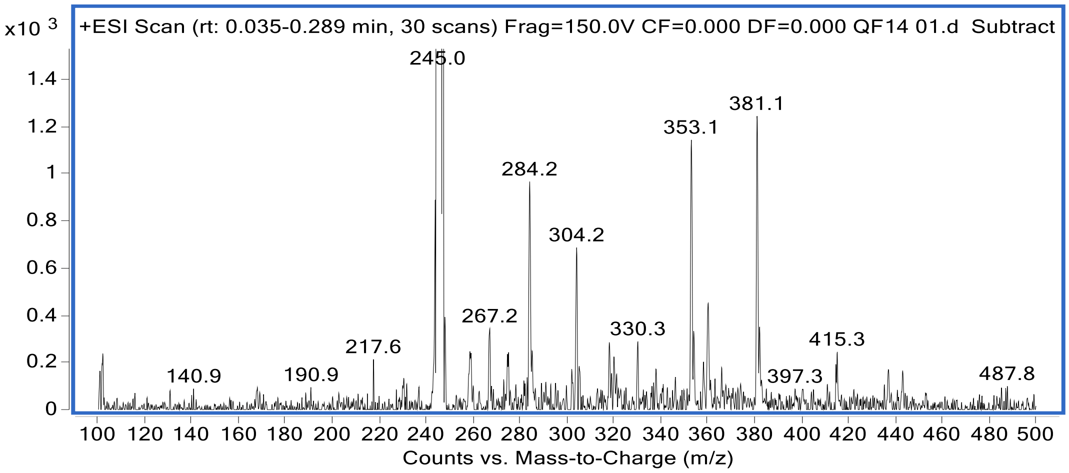

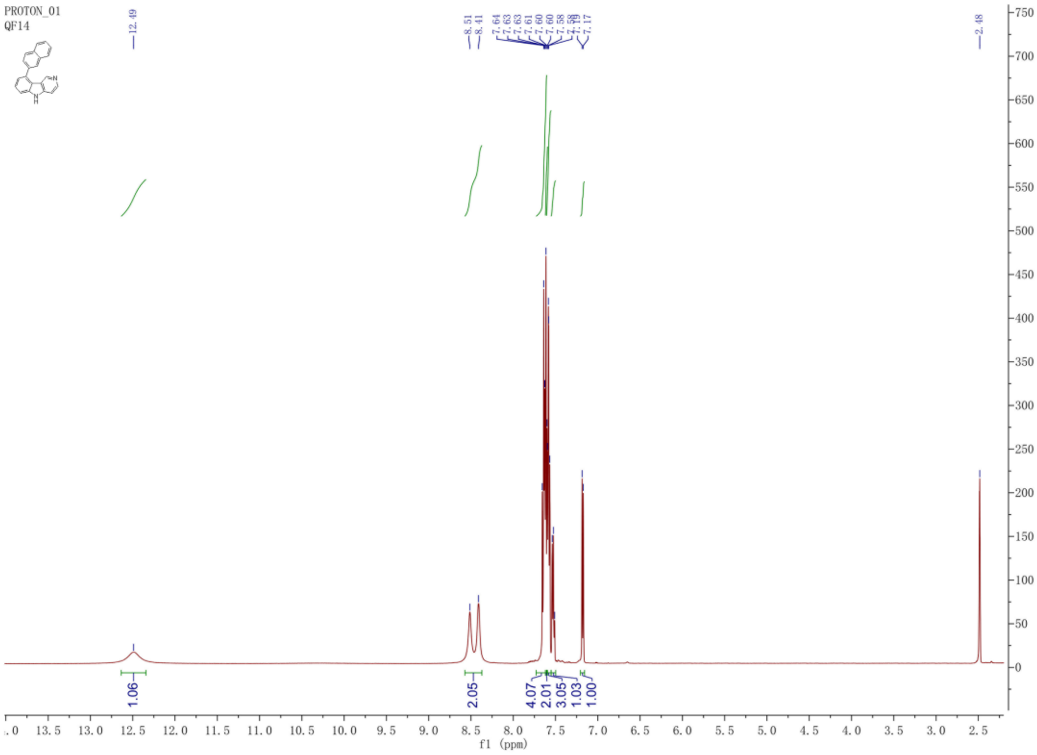

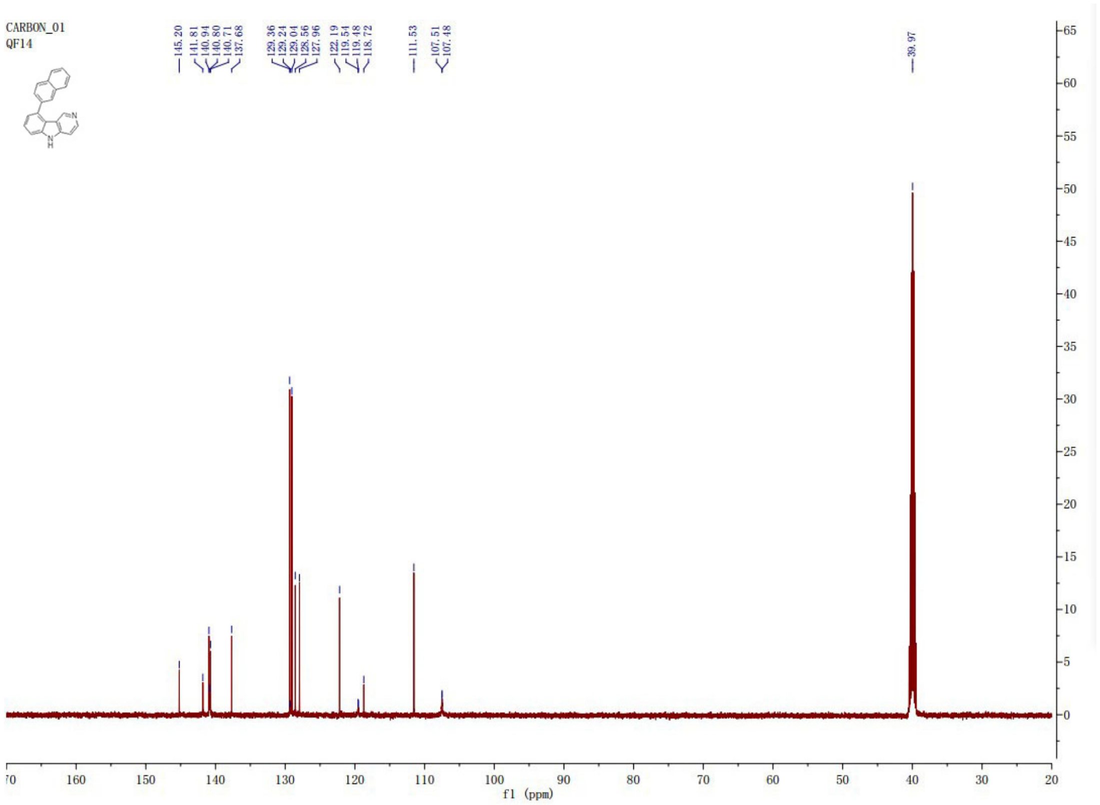

*9-(Thiophen-3-yl)-5H-pyrido[4,3-b]indole (**7r**)*

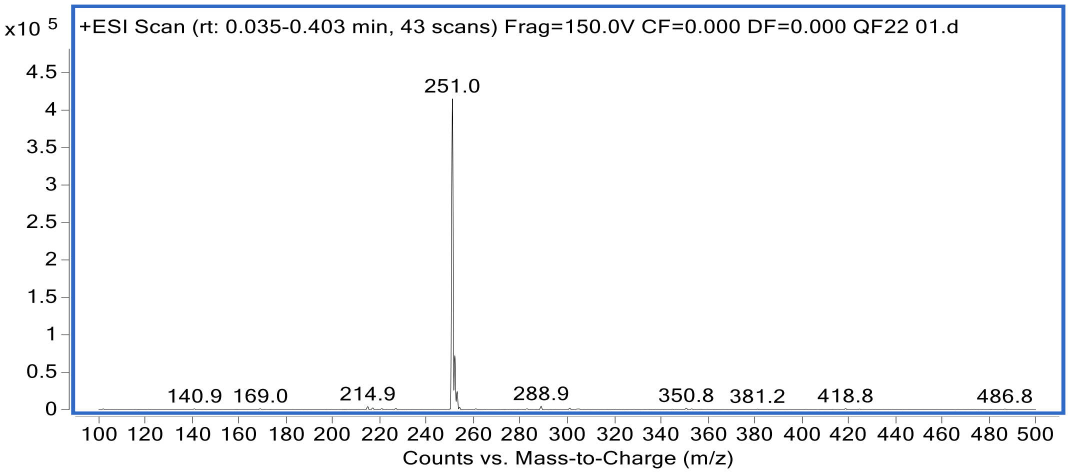

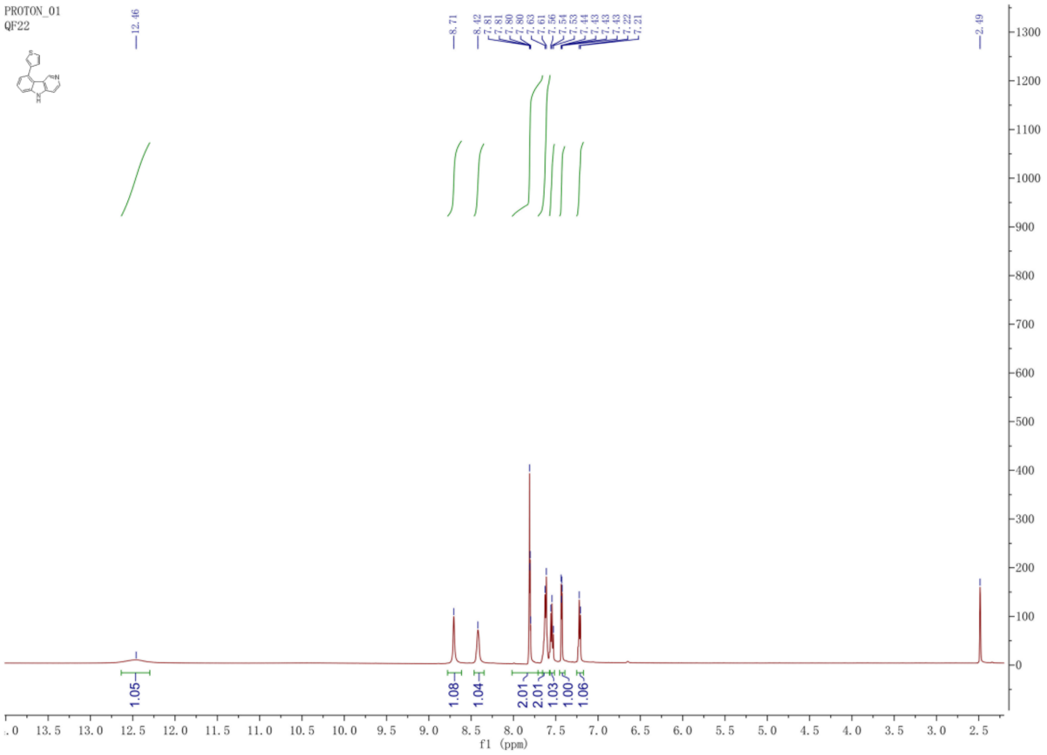

PROTON_01
QF22
12.46
8.71
8.42
7.81
7.81
7.80
7.80
7.63
7.61
7.56
7.54
7.53
7.44
7.43
7.43
7.43
7.22
7.21
2.49
1.05
1.08
1.04
2.01
2.01
1.03
1.00
1.06
f1 (ppm)

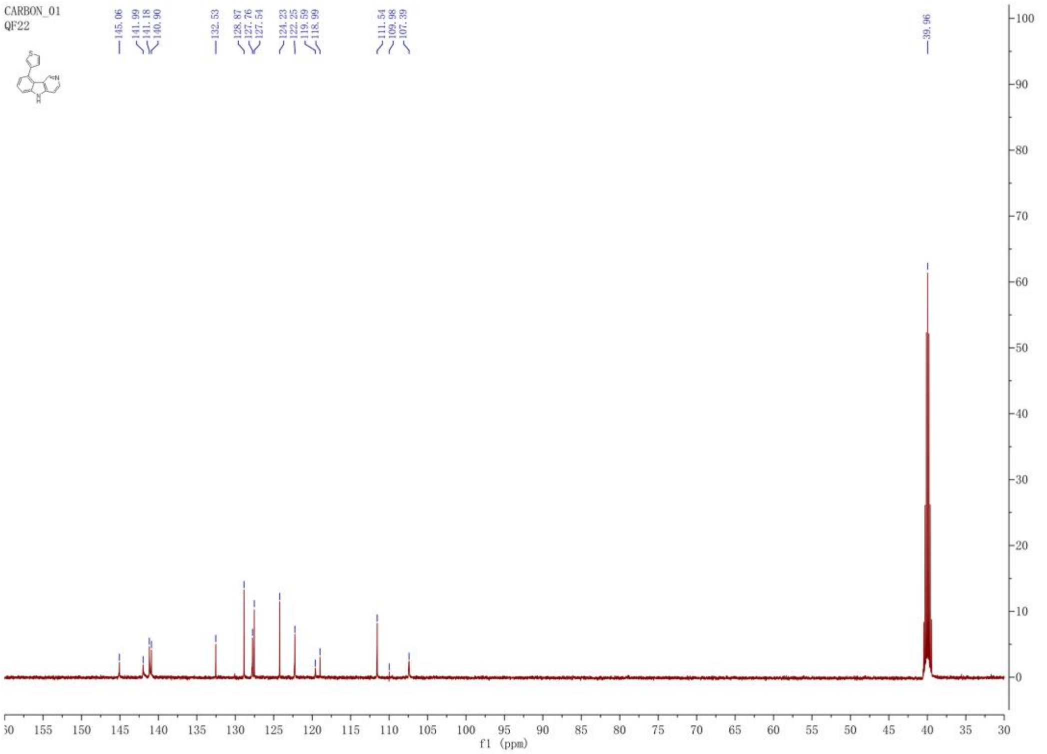

CARBON_01
QF22
145.06
141.99
141.18
140.90
132.53
128.87
127.76
127.54
124.23
122.25
119.59
118.99
111.54
109.98
107.39
39.96
f1 (ppm)

*9-(Pyridin-3-yl)-5H-pyrido[4,3-b]indole (**7s**)*

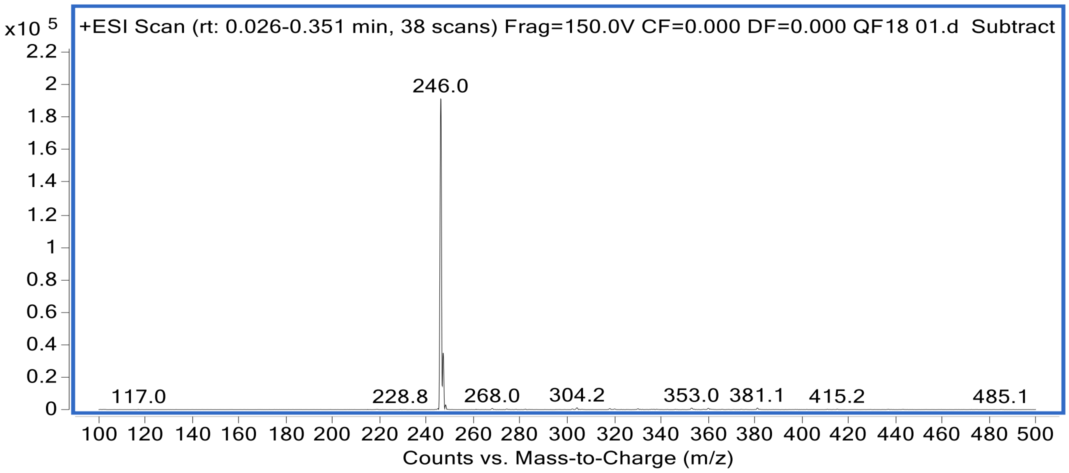

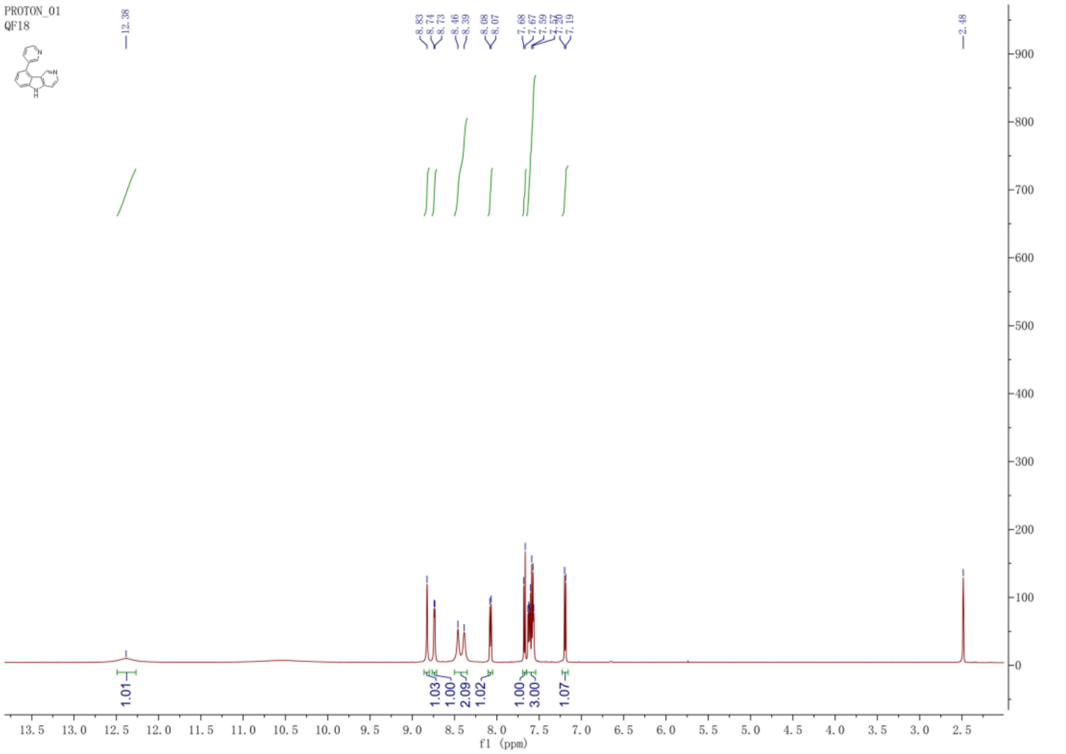

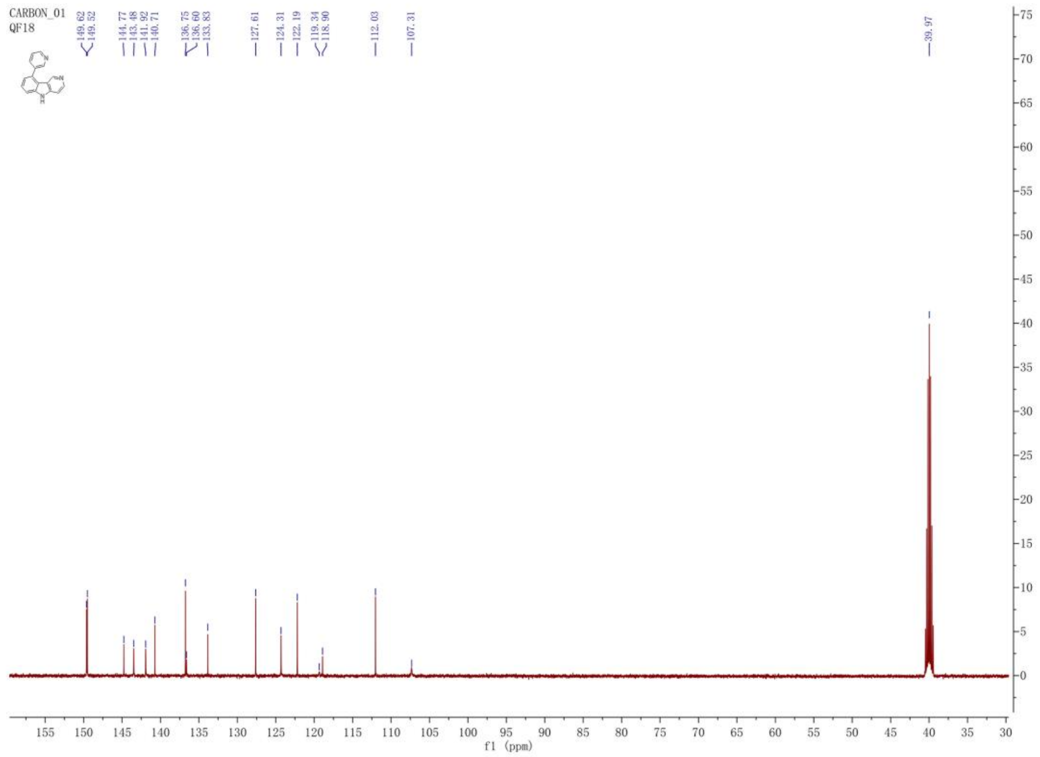

*9-(Pyridin-4-yl)-5H-pyrido[4,3-b]indole (**7t**)*

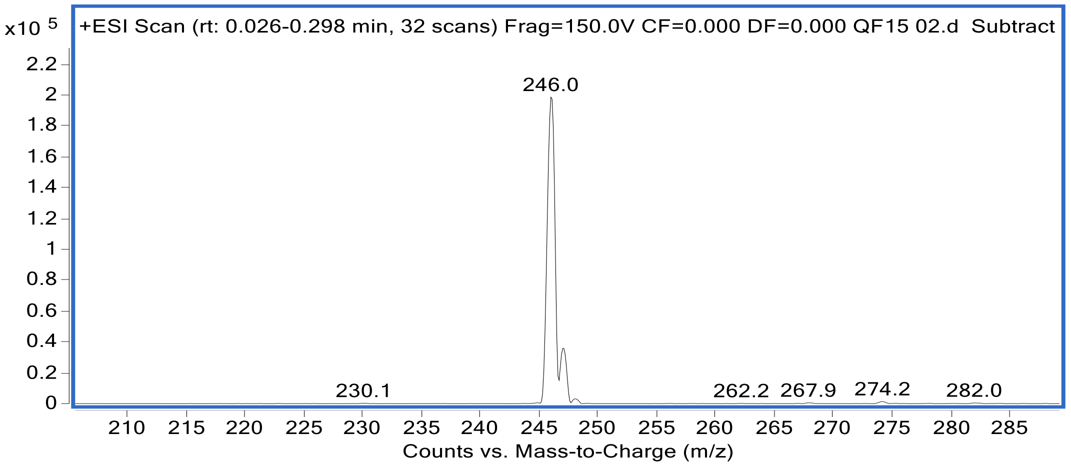

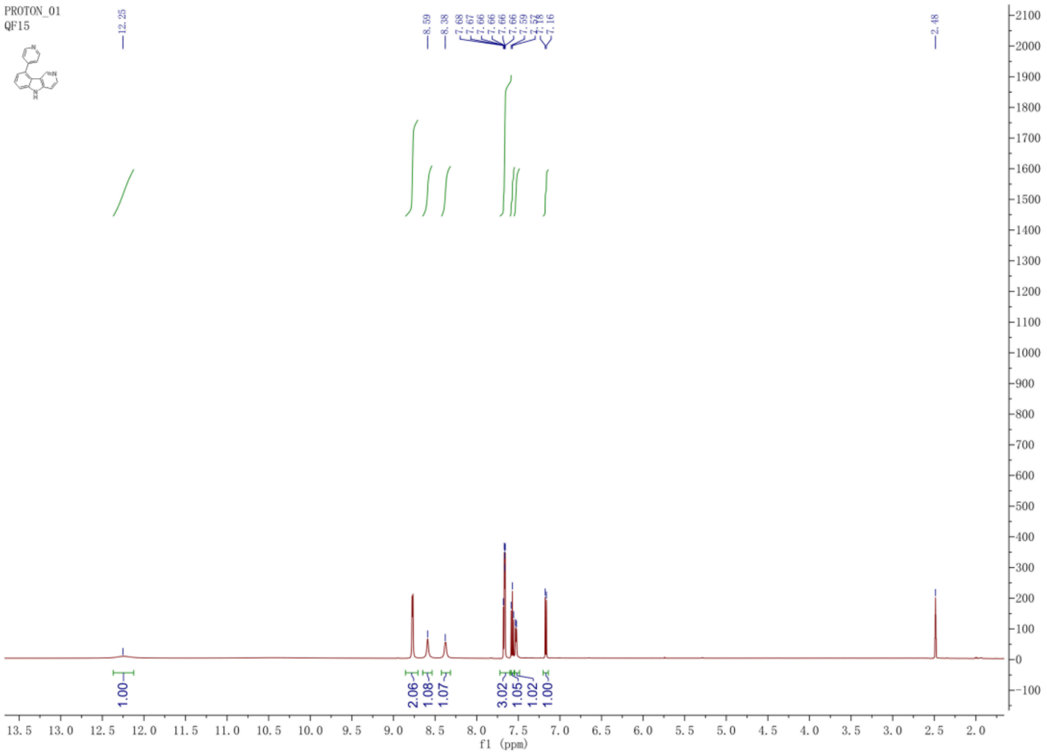
PROTON_01
QF15
12.25
8.59
8.38
7.68
7.67
7.66
7.66
7.66
7.66
7.59
7.57
7.18
7.16
2.48
1.00
2.06
1.08
1.07
3.02
1.05
1.02
1.00
f1 (ppm)

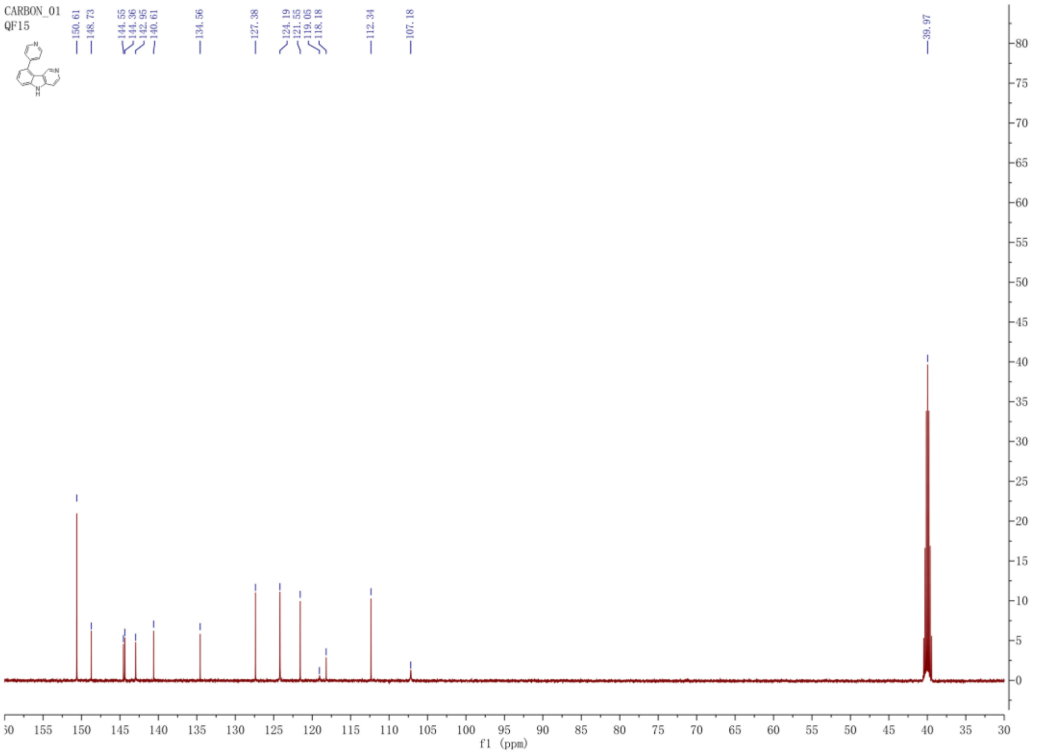
CARBON_01
QF15
150.61
148.73
144.55
144.36
142.85
140.61
134.56
127.38
124.19
121.55
119.05
118.18
112.34
107.18
39.97
f1 (ppm)

*9-(1H-indol-4-yl)-5H-pyrido[4,3-b]indole (**7u**)*

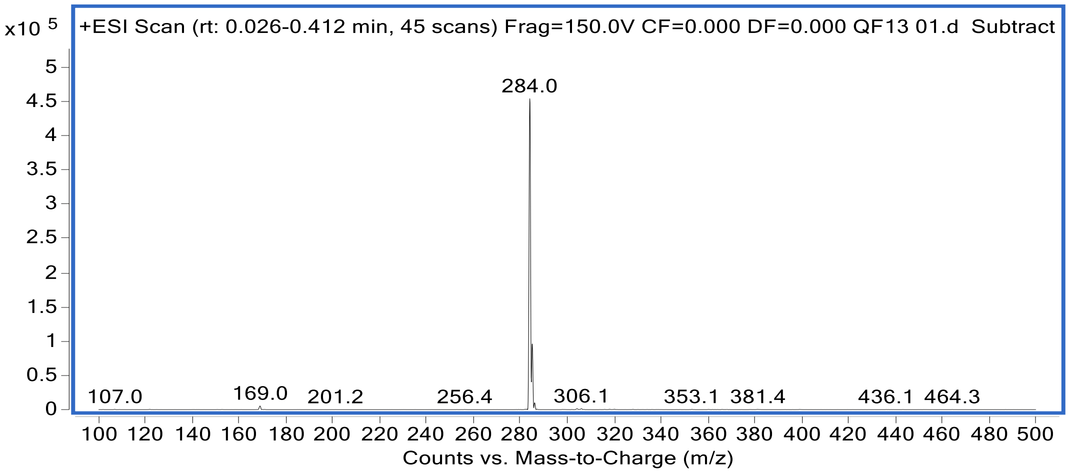

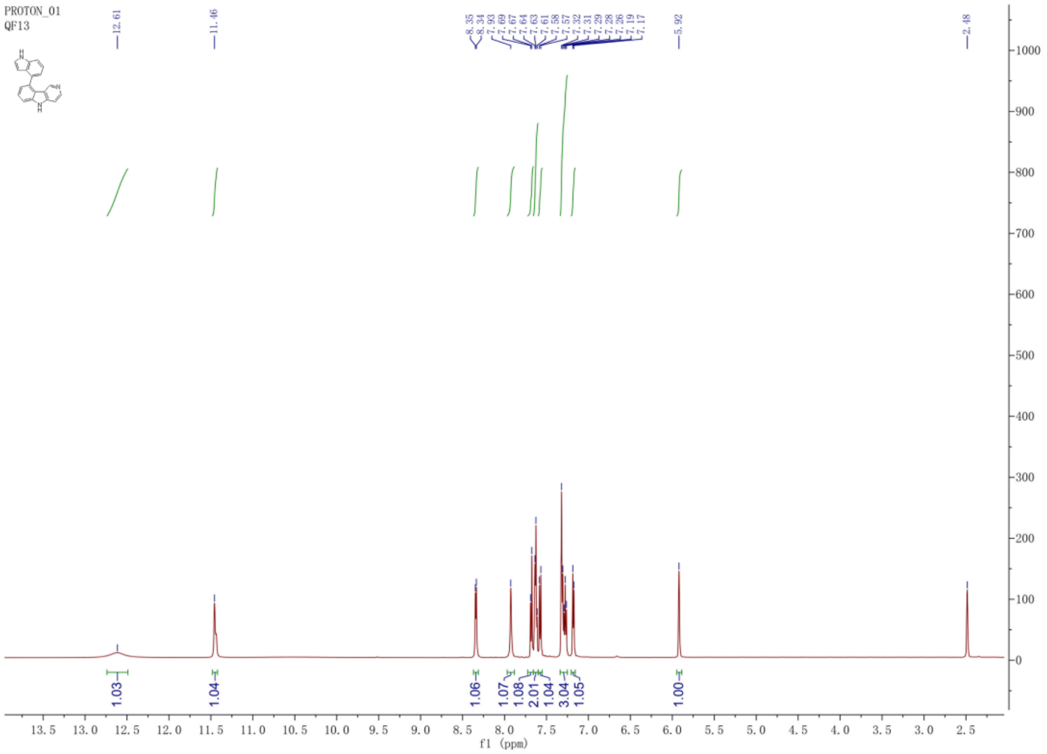

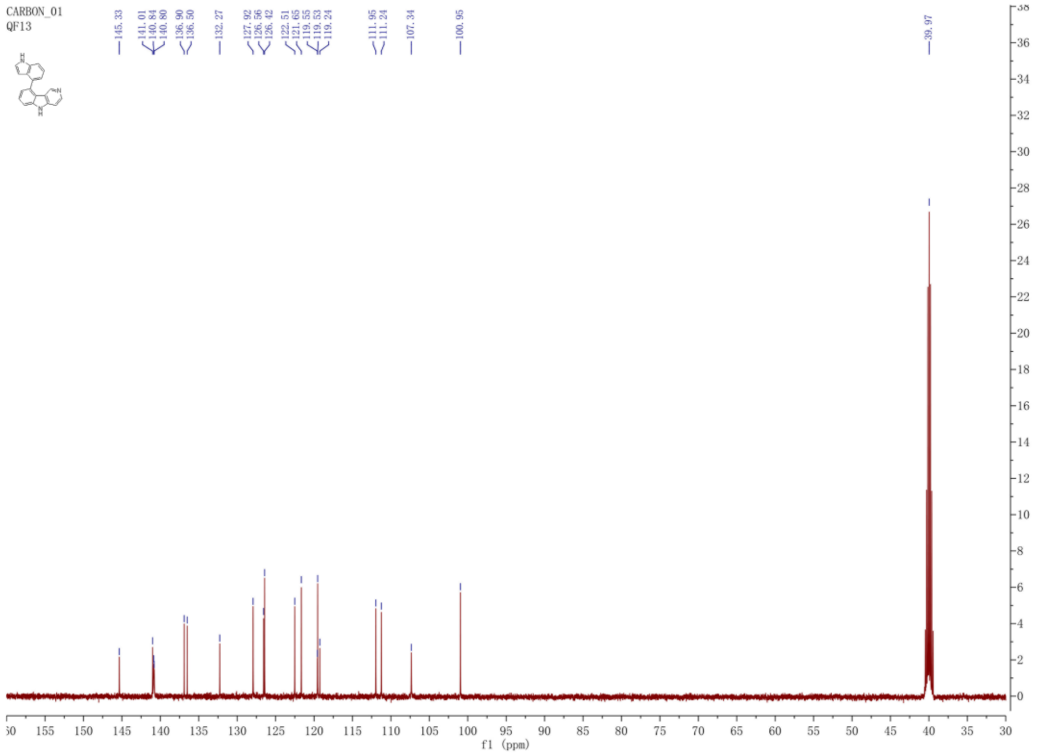

CARBON_01
QF13
145.33
141.01
140.84
140.80
136.90
136.50
132.27
127.92
126.56
126.42
122.51
121.65
119.55
119.53
119.24
111.95
111.24
107.34
100.95
39.97
f1 (ppm)
